# NEDD4L mediates intestinal epithelial cell ferroptosis to restrict inflammatory bowel diseases and colorectal tumorigenesis

**DOI:** 10.1172/JCI173994

**Published:** 2024-12-17

**Authors:** Jingjing Liang, Ning Wang, Yihan Yao, Yingmei Wang, Xiang An, Haofei Wang, Huan Liu, Yu Jiang, Hui Li, Xiaoqing Cheng, Jiaqi Xu, Xiaojing Liang, Jun Lou, Zengfeng Xin, Ting Zhang, Xiaojian Wang, Wenlong Lin

**Affiliations:** 1 The Second Affiliated Hospital, Zhejiang University School of Medicine, Hangzhou, China.; 2 School of Medicine, Hangzhou City University, Hangzhou, China.; 3 State Key Laboratory of Cancer Biology, Department of Pathology, Xijing Hospital and School of Basic Medicine, Fourth Military Medical University, Xi’an, China.; 4 Institute of Basic and Translational Medicine, Xi’an Medical University, Xi’an, China.; 5 Department of Clinical Laboratory Medicine, Second Affiliated Hospital, Zhejiang University School of Medicine, Hangzhou, China.; 6 Department of Medical Oncology, Cancer Hospital of the University of Chinese Academy of Sciences (Zhejiang Cancer Hospital), Zhejiang, China.; 7 Department of Pathology and; 8 Department of Respiratory and Critical Medicine, Sir Run Run Shaw Hospital, Zhejiang University School of Medicine, Hangzhou, China.; 9 Department of Radiation Oncology, The Second Affiliated Hospital, Zhejiang University School of Medicine, Hangzhou, China.

**Keywords:** Cell biology, Inflammation, Epithelial transport of ions and water, Inflammatory bowel disease, Molecular biology

## Abstract

Various factors play key roles in maintaining intestine homeostasis. Disruption of the balance may lead to inflammatory bowel diseases and even colorectal cancer (CRC). Loss or gain of function of many key proteins can result in dysregulated intestinal homeostasis. Our research demonstrated that neural precursor cells expressed developmentally downregulated 4–like protein (NEDD4L, or NEDD4-2), a type of HECT family E3 ubiquitin ligase, played an important role in maintaining intestinal homeostasis. NEDD4L expression was significantly inhibited in intestinal epithelial cells (IECs) of patients with Crohn’s disease, ulcerative colitis, and CRC. Global KO of NEDD4L or its deficiency in IECs exacerbated colitis induced by dextran sulfate sodium (DSS) and 2,4,6-trinitrobenzene sulfonic acid (TNBS) and CRC induced by azoxymethane and DSS. Mechanistically, NEDD4L deficiency in IECs inhibited expression of the key ferroptosis regulator glutathione peroxidase 4 (GPX4) by reducing the protein expression of solute carrier family 3 member 2 (SLC3A2) without affecting its gene expression, ultimately promoting DSS-induced IEC ferroptosis. Importantly, ferroptosis inhibitors reduced the susceptibility of NEDD4L-deficient mice to colitis and colitis-associated CRC. Thus, NEDD4L is an important regulator in IEC ferroptosis, maintaining intestinal homeostasis, making it a potential clinical target for diagnosing and treating IBDs.

## Abstract 2

NEDD4L mediates intestinal epithelial cell ferroptosis to restrict IBDs and tumorigenesis in humans and mice

## Graphical abstract

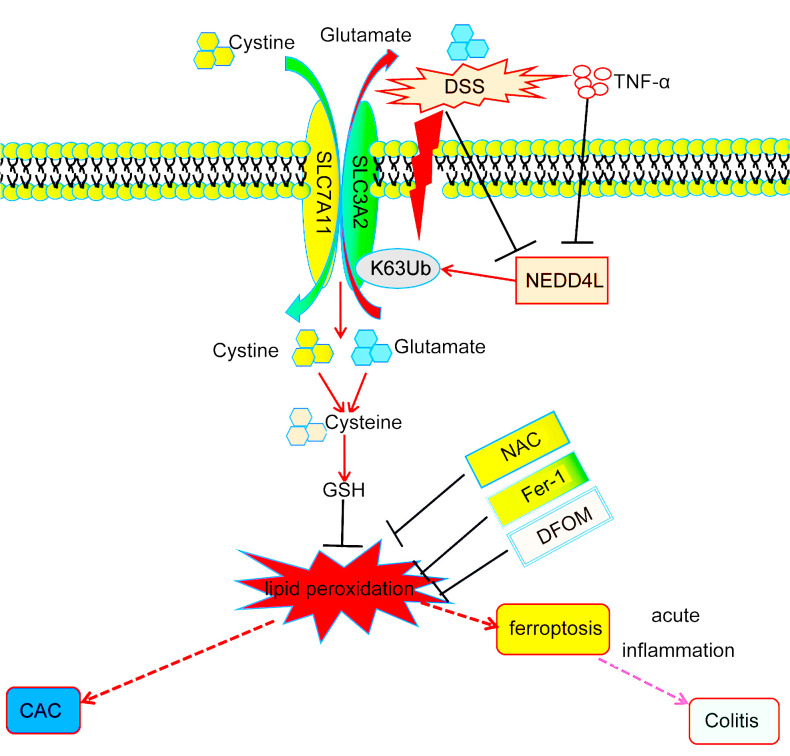

## Introduction

The intestinal mucosa is the largest mucosal surface that communicates with the environment, dietary antigens, and various microorganisms, serving as a critical component of immune regulation (1, 2). The intestinal mucosal barrier, composed of the intestinal epithelial cells (IECs), the immune barrier, and the intestinal flora barrier (3), jointly maintains intestinal homeostasis. Intestinal disorders caused by various factors, such as diet, genetic susceptibility, environmental factors, and mucosal immune disorders, contribute to the development of intestinal diseases, including colitis and colorectal cancer (CRC) (4). Therefore, maintaining intestinal mucosa homeostasis is crucial for controlling inflammation and preventing excessive immunopathology following inflammation.

Inflammatory bowel diseases (IBDs), including Crohn’s disease (CD) and ulcerative colitis (UC), are complicated diseases characterized by abnormal mucosal immune responses triggered by microorganisms, cytokines, and damaged epithelial cells, which can exacerbate the inflammation during the pathogenesis of colitis (4). Ferroptosis, a kind of cell death induced by excessive ferric ion levels and lipid peroxidation, exhibits a distinct morphology from other forms of cell death, such as apoptosis, necroptosis, and pyroptosis. Playing a crucial role in a variety of tissues and cell types, including neuron cells, renal tubular epithelial cells, endothelial cells, and T cells (5, 6), ferroptosis regulates diseases associated with cell death. Proteins like glutathione peroxidase 4 (GPX4), solute carrier family 7 member 11 (SLC7A11), and solute carrier family 3 member 2 (SLC3A2) directly or indirectly participate in the regulation of ferroptosis (5, 7). The ferroptosis of many tumor cells can be modulated by adjustment of the expression levels of GPX4, SLC7A11, and intracellular lipid peroxidation (8). However, only a few studies have reported on ferroptosis in intestine homeostasis (9, 10), and the regulatory function of SLC3A2 in ferroptosis remains largely unclear (11).

Numerous key proteins play important roles in maintaining the homeostasis of IECs (12, 13). E3 ubiquitin ligases, such as TNF-α–induced protein 3 (TNFAIP3; A20), baculoviral IAP repeat containing 2 (BIRC2; cIAP1), baculoviral IAP repeat containing 3 (BIRC3; cIAP2), tripartite motif containing 31 (TRIM31), ring finger protein 186 (RNF186), and membrane-associated ring-CH-type finger 3 (MARCH3), serve as key negative regulators in multiple signal pathways, participating in intestinal homeostasis by regulating immune response, IEC proliferation, apoptosis, or necroptosis (14–20). Neural precursor cells expressed developmentally downregulated 4–like protein (NEDD4L, or NEDD4-2), a member of the E3 ubiquitin ligase HECT family, is essential for maintaining cell homeostasis, as it can bind and regulate a variety of membrane proteins (21). NEDD4L has an amino-terminal Ca^2+^ phospholipid-binding (C2) domain, a protein-protein interaction (WW) domain, and a HECT domain located at the carboxyl terminal (22). The most clearly studied target of NEDD4L is the epithelial sodium channel (ENaC), which is usually expressed in lung and kidney epithelial cells, participating in related diseases (23–25). It also mediates the polyubiquitination and degradation of Smad2/3, thereby limiting the TGF-β signaling pathway (26). However, the regulatory role of NEDD4L in IBDs and colitis-associated colorectal cancer (CAC) remains unclear (27).

Here, we identified that both the gene and protein expression of NEDD4L was significantly inhibited in the IECs of patients with colitis and CRC, and negatively correlated with the disease status of colitis. NEDD4L deficiency in mice promoted colitis induced by dextran sulfate sodium (DSS) and 2,4,6-trinitrobenzene sulfonic acid (TNBS) and CRC induced by azoxymethane and DSS. Mechanistically, NEDD4L deficiency in IECs reduced the protein expression of the soluble amino acid transport protein SLC3A2 without affecting its gene expression. This led to inhibition of the expression of the key ferroptosis regulator GPX4, ultimately promoting DSS-induced IEC ferroptosis. Importantly, ferroptosis inhibitors, such as ferrostatin-1 and deferoxamine mesylate, reversed the colitis and CAC phenotype difference between WT and *Nedd4l* IEC-deficient (*Nedd4l^fl/fl^* *Villin^Cre^*) mice. Collectively, our data demonstrated that NEDD4L acted as an important regulator in IEC ferroptosis, thus maintaining intestinal homeostasis and controlling the development of colitis and CAC, suggesting that NEDD4L might be a potential target for the diagnosis and treatment of these diseases.

## Results

### NEDD4L expression is inhibited in IBDs.

**Figure 1:**
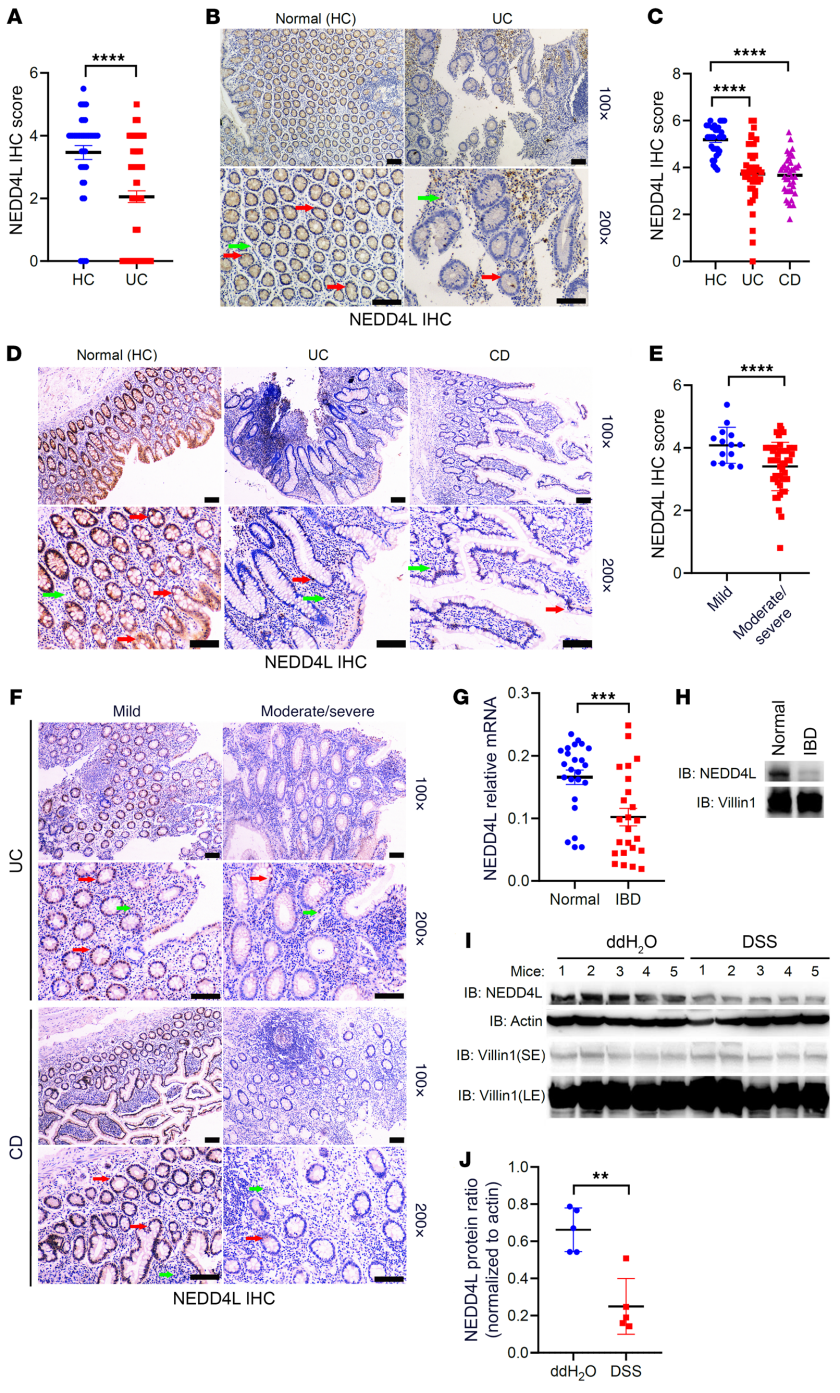
NEDD4L expression is significantly downregulated in IECs of patients with IBDs. (**A** and **B**) Statistical analysis of NEDD4L immunohistochemical (IHC) intensity in the biopsies from Xijing Hospital (cohort 1) (**A**) and representative IHC staining of sections traced with anti-NEDD4L antibody (**B**). Normal control (HC) *n* = 40 and UC *n* = 83. Scale bars: 50 μm. (**C** and **D**) Statistical analysis of NEDD4L IHC intensity in the biopsies from the First Affiliated Hospital of Zhejiang University School of Medicine (cohort 2) (**C**) and representative IHC staining of sections (**D**). Normal control (HC) *n* = 31, UC *n* = 36, and CD *n* = 41. Scale bars: 50 μm. (**E** and **F**) Statistical analysis of NEDD4L IHC intensity in the biopsies with disease status record from cohort 2 (**E**) and representative IHC staining of sections traced with anti-NEDD4L antibody (**F**). Mild *n* = 14 and moderate/severe *n* = 48. Scale bars: 50 μm. (**G** and **H**) Quantitative PCR (qPCR) analysis (**G**) and representative Western blotting (**H**) of NEDD4L in the mucosa from patients with IBDs and their corresponding normal tissues (*n* = 24 per group). (**I** and **J**) Western blotting analysis (**I**) and protein intensity analysis (**J**) according to **I** using ImageJ software (NIH) of NEDD4L from the IECs of the WT mice treated or not treated with DSS for 4 days (*n* = 5 per group). Red arrows indicate NEDD4L expression in IECs, and green arrows indicate NEDD4L expression in non-IECs. Data represent mean ± SEM. Each dot represents an independent sample. **P < 0.01; ***P < 0.001; ****P < 0.0001. Statistical analysis was performed using 1-way ANOVA with multiple comparisons in **C**, and a 2-tailed Student’s *t* test in **A**, **E**, **G**, and **J**.

Our previous data have demonstrated that NEDD4L plays a crucial role in IL-17–, IL-6–, and virus-mediated innate immune responses (28–30). However, its role in intestinal homeostasis remains unclear. To explore the potential function of NEDD4L in intestinal homeostasis, we first analyzed *NEDD4L* gene expression in the public database The Human Protein Atlas (https://www.proteinatlas.org). As shown in Supplemental Figure 1, A and B (supplemental material available online with this article; https://doi.org/10.1172/JCI173994DS1), the *NEDD4L* gene was highly expressed in human neuron, lung, and intestinal systems, most highly in goblet cells, but was lowly expressed in the human immune system, indicating that highly expressed *NEDD4L* in intestinal epithelium might be involved in maintaining intestinal homeostasis. We analyzed the gene expression of *NEDD4L* in patients with IBDs from Gene Expression Omnibus (GEO) datasets. As shown in Supplemental Figure 1, C–E, compared with healthy controls (HCs), *NEDD4L* gene expression in colonic mucosa was restricted in patients with CD and UC. Nevertheless, *NEDD4L* gene expression was significantly increased in PBMCs from patients with CD and UC compared with HCs (Supplemental Figure 1F). Two cohorts of study subjects from Xijing Hospital (cohort 1) and First Affiliated Hospital of Zhejiang University School of Medicine (cohort 2) were recruited to trace the NEDD4L protein expression in the colonic biopsies. As shown in Figure 1, A–D, the NEDD4L protein level in IECs was significantly reduced in patients with UC and CD compared with the normal control subjects (HCs). In the samples from cohort 1, only 4.8% of the biopsies from patients with UC (4/83) exhibited strong NEDD4L immunohistochemistry (IHC) staining, whereas 20% of the healthy control subjects (8/40) showed strong NEDD4L IHC staining (*P* < 0.001; Table 1). Similar results were observed in cohort 2, only 38.8% of the UC patient biopsies (14/36) and 39.0% of the CD patient biopsies (16/41) exhibited strong NEDD4L IHC staining, whereas 96.8% of the healthy control subjects (30/31) showed strong NEDD4L IHC staining (*P* < 0.001; Table 2). Importantly, NEDD4L protein expression was lower in patients with moderate or severe colitis than in those with mild colitis from cohort 2 (Figure 1, E and F), consistent with the GEO data (Supplemental Figure 1G), indicating that NEDD4L expression was negatively correlated with the severity of colitis. Similarly, *NEDD4L* gene expression in colonic mucosa was significantly inhibited in the diseased individual from monozygotic twin pairs discordant for UC compared with the healthy individual (Supplemental Figure 1H), suggesting that the reduced expression of *NEDD4L* was likely to be a consequence of IEC damage or inflammation. To further explore the specific expression profile of *NEDD4L* in IECs, a single-cell RNA analysis was performed. Compared with healthy tissue, the gene expression of *NEDD4L* in inflamed colon tissues from patients with UC was significantly inhibited in enterocytes (including bestrophin 4–positive [Best4^+^] enterocytes and immature enterocytes), goblet cells, transit-amplifying (TA) cells (including TA1, TA2, cycling TA, and secretory TA cells), and stem cells, but not significantly changed in enterocyte progenitors, enteroendocrine cells, immature enterocytes, M cells, and tuft cells (Supplemental Figure 1I). Furthermore, both the gene and protein expression of NEDD4L in patients with IBDs was significantly inhibited in comparison with the normal colon mucosa (Figure 1, G and H). Additionally, upon DSS treatment in mice, both the gene and protein expression of NEDD4L in IECs was significantly inhibited (Figure 1, I and J, and Supplemental Figure 1, J and K). Collectively, these results suggest that the NEDD4L gene and protein were significantly inhibited in humans and mice with colitis, and NEDD4L expression was correlated with severity in patients with IBDs.

### Nedd4l deficiency in mice enhances sensitivity to experimental colitis.

**Figure 2:**
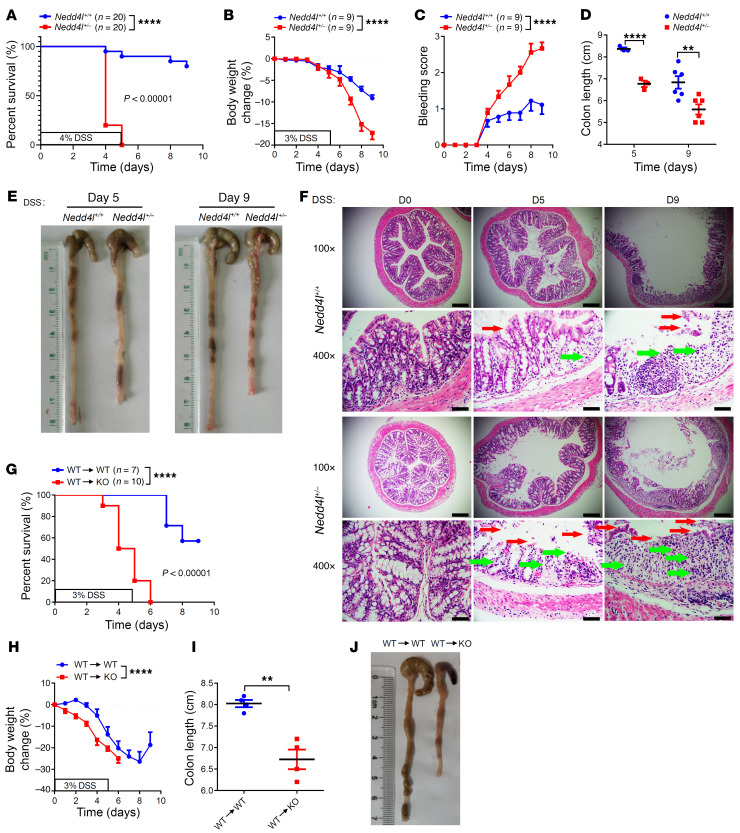
*Nedd4l* deficiency in mice promotes DSS-induced experimental colitis in a non-hematopoietic cell–dependent manner. (**A**) *Nedd4l*-global-deficient mice (*Nedd4l^+/–^*) and control littermates (*Nedd4l^+/+^*) were given 4% DSS for 5 days followed by water to induce acute colitis. Mouse death was monitored until day 9. *n* = 20 per group. (**B**–**F**) *Nedd4l^+/–^* and *Nedd4l^+/+^* mice were given 3% DSS for 5 days followed by water until day 9. *n* = 9 per group. Body weight change (**B**), bleeding scores (**C**), colon length (**D**), gross morphology images (**E**), and H&E staining of colons (**F**) from *Nedd4l^+/+^* and *Nedd4l^+/–^* mice are shown. Red arrows point to epithelial degeneration and green arrows to inflammatory infiltrates. Scale bars: 200 μm or 50 μm (amplified sections). (**G**–**J**) Bone marrow from *Nedd4l^+/+^* (WT) and *Nedd4l^–/–^* (KO) mice was transferred to WT (*n* = 7) and KO (*n* = 10) mice to generate bone marrow reconstitution mice. The bone marrow reconstitution mice were subjected to 3% DSS treatment for 5 days followed by water, and mouse death (**G**) and body weight changes (**H**) were monitored until day 9. (**I** and **J**) From a separate experiment, colon length (**I**) and gross morphology images (**J**) of colons from mice on day 6 after DSS treatment. *n* = 4 per group. Data represent mean ± SEM from at least 2 independent experiments. Each dot represents an independent sample. **P < 0.01; ****P < 0.0001. Statistical analysis was performed using a log-rank test in **A** and **G**, a 2-way ANOVA test in **B**, **C**, and **H**, and a 2-tailed Student’s *t* test in **D** and **I**.

To investigate the role of NEDD4L in colitis, mice with heterogeneous KO of *Nedd4l* (*Nedd4l^+/–^*) and control WT littermates (*Nedd4l^+/+^*) were initially challenged with 4% DSS to induce an acute experimental colitis model. The mortality rate was significantly higher in *Nedd4l^+/–^* mice compared with *Nedd4l^+/+^* mice (Figure 2A). Remarkably, we observed more severe colitis after 3% DSS treatment in *Nedd4l^+/–^* mice compared with *Nedd4l^+/+^* mice, as evidenced by significantly greater body weight loss, higher rectal bleeding score, and shorter colons in DSS-treated *Nedd4l^+/–^* mice (Figure 2, B–F). Furthermore, mice with global deficiency of *Nedd4l* (*Nedd4l^–/–^*, KO) exhibited a more severe colitis phenotype when treated with a very low dosage of DSS (1%); however, it was hard to induce an obvious colitis phenotype in *Nedd4l^+/–^* and *Nedd4l^+/+^* mice, suggesting that *Nedd4l* KO increased the susceptibility of mice to low-dose DSS exposure (Supplemental Figure 2, A–E).

To determine whether *Nedd4l* deficiency in IECs or hematopoietic cells contributes to the more severe colitis phenotype, bone marrow chimera experiments were conducted. Lethally irradiated *Nedd4l^+/+^* (WT) and *Nedd4l^–/–^* (KO) mice were reconstituted with bone marrow cells from WT mice. Mice reconstituted with *Nedd4l* deficiency in non-hematopoietic cells (WT→KO) exhibited a more severe colitis phenotype compared with the *Nedd4l^+/+^* chimeras (WT→WT) following DSS treatment (Figure 2, G–J). Collectively, these data implicate that NEDD4L in non-hematopoietic cells promoted the pathogenesis of DSS-induced colitis.

### Nedd4l deficiency in IECs exacerbates DSS-induced and TNBS-induced experimental colitis.

**Figure 3:**
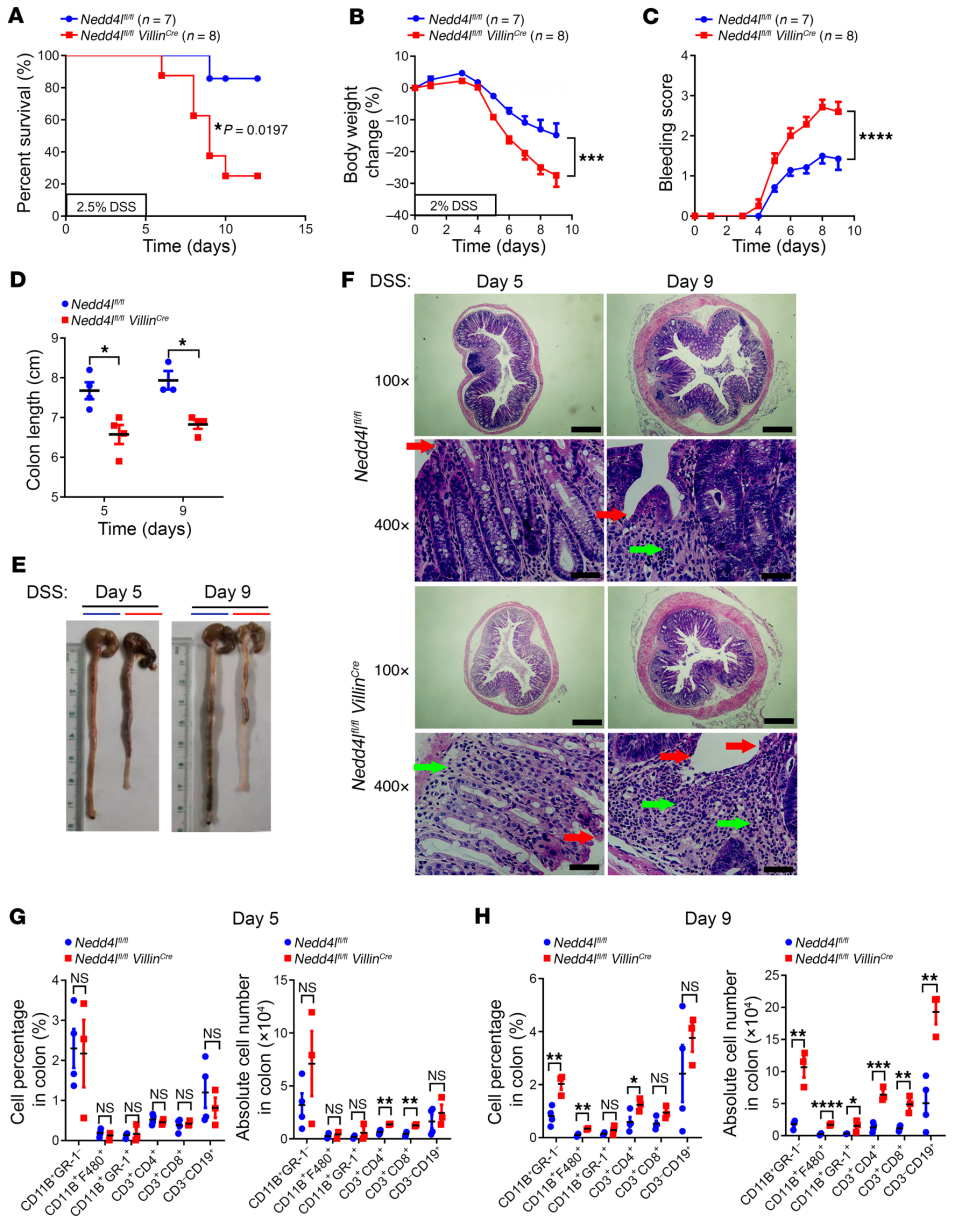
*Nedd4l* deficiency in IECs promotes DSS-induced colitis in mice. (**A**) *Nedd4l* IEC-deficient mice (*Nedd4l^fl/fl^ Villin^Cre^*, *n* = 8) and control littermates (*Nedd4l^fl/fl^*, *n* = 7) were given 2.5% DSS for 5 days followed by water to induce acute colitis. Mouse death was monitored until day 12. (**B**–**F**) In a separate experiment, *Nedd4l^fl/fl^ Villin^Cre^* mice (*n* = 7) and control *Nedd4l^fl/fl^* mice (*n* = 8) were given 2% DSS for 5 days followed by water until day 9 to induce colitis. Body weight change (**B**), bleeding scores (**C**), colon length (**D**), gross morphology images (**E**), and H&E staining of the colons (**F**) from *Nedd4l^fl/fl^ Villin^Cre^* and *Nedd4l^fl/fl^* mice are shown. Red arrows point to epithelial degeneration and green arrows to inflammatory infiltrates. Scale bars: 200 μm or 50 μm (amplified sections). (**G** and **H**) Colon-infiltrated immune cells of *Nedd4l^fl/fl^ Villin^Cre^* and *Nedd4l^fl/fl^* mice from **B** were analyzed by flow cytometry analysis (*n* = 3–4 per group). Data represent mean ± SEM from at least 2 independent experiments. Each dot represents an independent sample. *P < 0.05; **P < 0.01; ***P < 0.001; ****P < 0.0001. Statistical analysis was performed using a log-rank test in **A**, a 2-way ANOVA test in **B** and **C**, and a 2-tailed Student’s *t* test in **D**, **G**, and **H**.

To further explore whether the protective role of NEDD4L in colitis was intrinsic to IECs, we generated mice with IEC-specific KO of *Nedd4l* (*Nedd4l^fl/fl^ Villin^Cre^*) by crossing *Nedd4l*-floxed mice (*Nedd4l^fl/fl^*) with *Villin^Cre^* mice, resulting in constitutive deletion of *Nedd4l* in the IECs. Consistent with previous reports (31), *Nedd4l^fl/fl^ Villin^Cre^* mice displayed normal intestinal histology. The terminally differentiated cells were indistinguishable between WT and *Nedd4l^fl/fl^ Villin^Cre^* mice under steady-state conditions (Supplemental Figure 2, F and G). In addition, assessment of the numbers of goblet cells, Paneth cells, enteroendocrine cells, and enterocytes, identified by periodic acid–Schiff, lysozyme, chromogranin A, and alkaline phosphatase (ALP) staining, respectively, revealed no obvious difference in terms of cell lineage commitment (Supplemental Figure 2, F–I). This observation was further confirmed by quantitative PCR analysis, which showed no significant alterations in the expression of marker genes for the different cell lineages and stem cell populations in intestinal tissue from *Nedd4l^fl/fl^ Villin^Cre^* mice compared with control *Nedd4l^fl/fl^* mice (Supplemental Figure 2, J and K). However, *Nedd4l^fl/fl^ Villin^Cre^* mice showed a significantly higher death rate than control littermates upon 2.5% DSS treatment (Figure 3A). *Nedd4l^fl/fl^ Villin^Cre^* mice exhibited more severe weight loss, rectal bleeding, colon shortening, epithelial damage, and crypt architecture disruption than *Nedd4l^fl/fl^* mice when challenged with 2% DSS (Figure 3, B–F). Additionally, a 5-day DSS treatment induced comparable degrees and absolute cell numbers of mucus-infiltrated monocytes, macrophages, and neutrophils, but increased absolute cell numbers of mucus-infiltrated T cells and B cells, in *Nedd4l^fl/fl^ Villin^Cre^* mice compared with the control littermates (Figure 3G). Moreover, following the development of colitis, particularly on day 9, much more inflammatory immune cell infiltration in mucus was observed in *Nedd4l^fl/fl^ Villin^Cre^* mice compared with *Nedd4l^fl/fl^* mice, including monocytes, macrophages, T cells, and B cells (Figure 3H).

We then investigated whether *Nedd4l* deficiency might exacerbate colitis in an alternative model induced by TNBS. As expected, compared with the control group, TNBS-treated *Nedd4l^fl/fl^ Villin^Cre^* mice phenocopied the aggravated symptoms of colitis as in DSS-treated *Nedd4l^fl/fl^ Villin^Cre^* mice (Supplemental Figure 3, A–F). Collectively, these data support the notion that *Nedd4l* deficiency in IECs contributed to both DSS-induced and TNBS-induced colonic damage and colitis.

### Nedd4l deficiency in IECs promotes IEC ferroptosis and subsequent intestinal barrier integrity damage.

**Figure 4:**
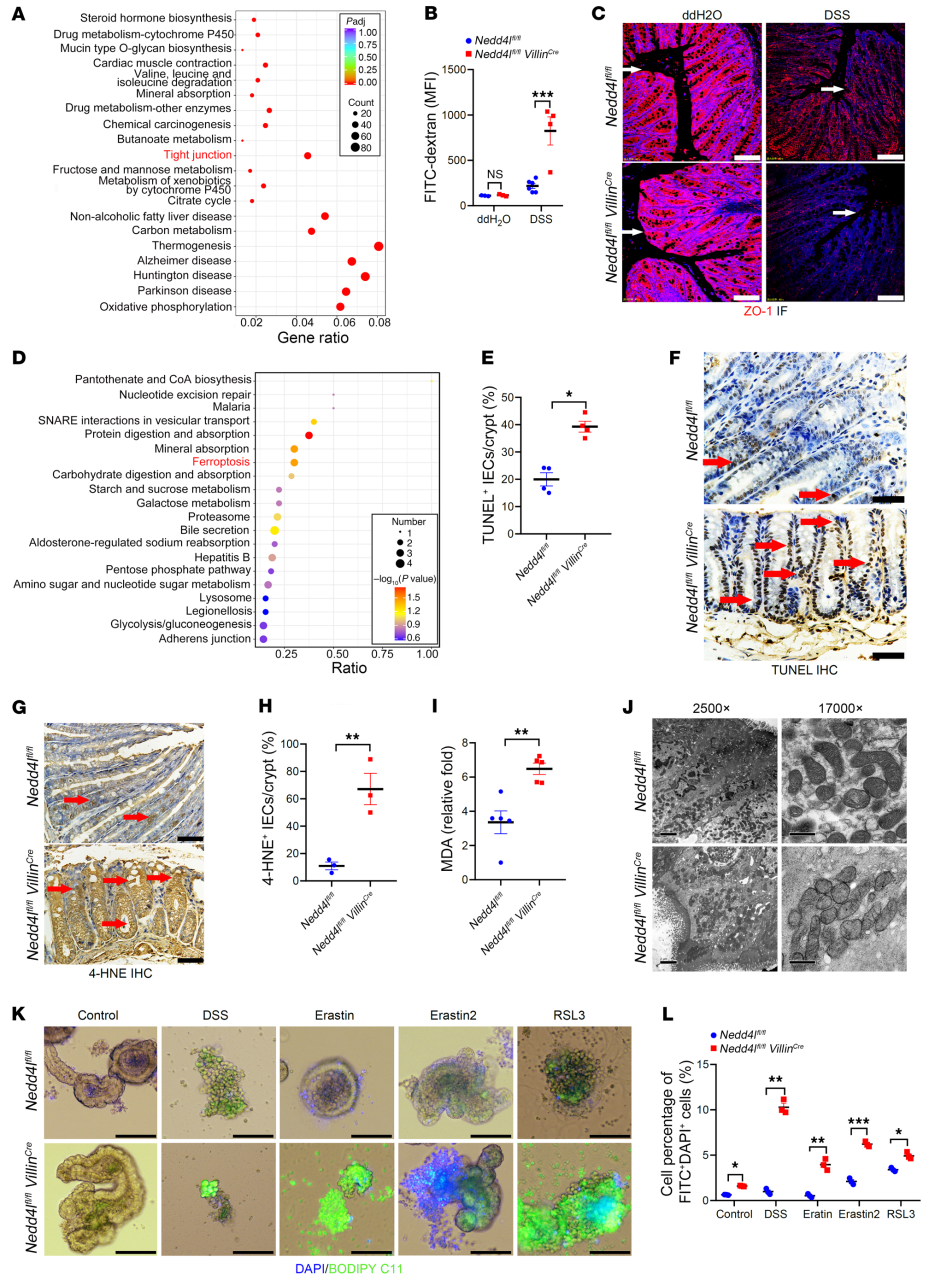
*Nedd4l* deficiency in IECs promotes IEC ferroptosis, resulting in barrier integrity damage. (**A**) KEGG analysis of colonic tissues on the 7th day from *Nedd4l^fl/fl^ Villin^Cre^* and *Nedd4l^fl/fl^* mice given 2% DSS. (**B**) The indicated mice were treated as in **A** and were orally fed with FITC-dextran (500 mg/kg) for 4 hours before sacrifice. The serum levels of FITC-dextran were detected by measurement of the mean fluorescence intensity (MFI) of FITC-dextran. (**C**) In a separate experiment, the indicated mice were treated as in **A**, and colon tissues were further subjected to ZO-1 immunofluorescence (IF) staining. Red IF indicates ZO-1, and blue (DAPI) indicates cell nucleus. Scale bars: 50 μm. (**D**) KEGG analysis of ubiquitylation mass spectrometry (MS) from IECs of the indicated mice treated as in **A**. (**E**–**H**) Colon tissues from DSS-treated *Nedd4l^fl/fl^ Villin^Cre^* and *Nedd4l^fl/fl^* mice were subjected to TUNEL (**E** and **F**) and 4-hydroxynonenal (4-HNE) (**G** and **H**) IHC staining. TUNEL (**F**) and 4-HNE (**H**) IHC staining was scored and analyzed. Scale bars: 50 μm. (**I**) In a separate experiment, IECs from DSS-treated *Nedd4l^fl/fl^ Villin^Cre^* and *Nedd4l^fl/fl^* mice were subjected to MDA analysis. (**J**) Representative transmission electron microscope images from colonic tissue sections of DSS-treated *Nedd4l^fl/fl^ Villin^Cre^* and *Nedd4l^fl/fl^* mice. Scale bars: 2 μm or 0.5 μm (amplified sections). (**K** and **L**) Representative microscope images (**K**) and flow cytometer analysis (**L**) of small-intestinal organoids isolated and cultured from crypts of *Nedd4l^fl/fl^ Villin^Cre^* and *Nedd4l^fl/fl^* mice treated with DMSO (Control), DSS (0.5% wt/vol), erastin (30 μM), erastin 2 (30 μM), and RSL3 (5 μM) for 24 hours, followed by DAPI and BODIPY C11 staining. *n* = 3 per group. Scale bars: 100 μm. Data represent mean ± SEM from at least 2 independent experiments. Each dot represents an independent sample. *P < 0.05; **P < 0.01; ***P < 0.001. Statistical analysis was performed using a 2-tailed Student’s *t* test in **B**, **E**, **H**, **I**, and **L**.

To explore the underlying mechanisms of NEDD4L in regulating colitis, colonic tissues from DSS-treated *Nedd4l^fl/fl^ Villin^Cre^* mice and *Nedd4l^fl/fl^* littermates were subjected to RNA sequencing analysis. As shown in Figure 4A, the tight junction signaling was significantly downregulated in *Nedd4l^fl/fl^ Villin^Cre^* mice compared with *Nedd4l^fl/fl^* littermates. Furthermore, the *Nedd4l^fl/fl^ Villin^Cre^* mice displayed higher serum FITC-dextran concentrations after DSS treatment than *Nedd4l^fl/fl^* mice, while displaying similar epithelial permeability to *Nedd4l^fl/fl^* mice in the absence of DSS treatment (Figure 4B). Additionally, histopathological analysis by tight junction protein 1 (ZO-1) immunofluorescence staining showed that *Nedd4l* deficiency led to a more severely diminished expression of ZO-1 in the mucosal epithelium in response to DSS treatment (Figure 4C).

To further explore the regulation of barrier integrity during the induction of colitis by IEC-derived *Nedd4l*, the IECs from *Nedd4l^fl/fl^ Villin^Cre^* mice and *Nedd4l^fl/fl^* littermates with or without DSS treatment were subjected to quantitative ubiquitination mass spectrometry (MS) analysis. As shown in Supplemental Figure 4A, the Gene Ontology (GO) analysis showed that the markedly changed potential substrates mainly regulated protein localization, transport, and transport activity. The Kyoto Encyclopedia of Genes and Genomes (KEGG) analysis showed that protein digestion and absorption, mineral absorption, and ferroptosis signaling pathways were markedly enriched in IECs from *Nedd4l^fl/fl^ Villin^Cre^* mice compared with *Nedd4l^fl/fl^* mice (Figure 4D and Supplemental Figure 4B). In comparison with WT littermates, the levels of TUNEL^+^ epithelial cells, as well as the lipid peroxidation measured by cells staining positive for 4-hydroxynonenal and by malondialdehyde (MDA) contents, were remarkably enhanced in DSS-treated *Nedd4l^fl/fl^ Villin^Cre^* mice, suggesting that *Nedd4l* deficiency in IECs promoted lipid peroxidation–mediated IEC death after DSS treatment (Figure 4, E–I). IECs from *Nedd4l^fl/fl^ Villin^Cre^* mice exhibited much more severe ferroptosis morphology, characterized by mitochondrial fragmentation, disappearance of internal cristae, and collapse, compared with those from *Nedd4l^fl/fl^* mice (Figure 4J). Consistently, the expression levels of ferroptosis and proinflammatory-related genes, such as *Gpx4*, were significantly restricted in *Nedd4l^fl/fl^ Villin^Cre^* mice relative to *Nedd4l^fl/fl^* mice, while the gene expression levels of transferrin receptor protein 1 (*TfR1*, also known as *Tfrc*), prostaglandin-endoperoxide synthase 2 (*Ptgs2*), and lipocalin 2 (*Lcn2*) were significantly increased in *Nedd4l^fl/fl^ Villin^Cre^* mice (Supplemental Figure 4, C and D). Furthermore, we stimulated the intestine organoids derived from *Nedd4l^fl/fl^ Villin^Cre^* mice and *Nedd4l^fl/fl^* mice with DSS and ferroptosis inducers in vitro, including erastin, erastin 2 (a specific glutamine/cystine transporter inhibitor), and RSL3, to check whether NEDD4L could mediate IEC ferroptosis. As shown in Figure 4, K and L, *Nedd4l* deficiency in IECs promoted lipid peroxidation–mediated IEC death, which was assessed by 4′,6-diamidino-2-phenylindole (DAPI; indicating the dead cell) and FITC–BODIPY C11 staining (indicating intercellular lipid peroxidation production). Our data suggest that NEDD4L maintained intestinal barrier integrity by inhibiting IEC ferroptosis.

We had noticed that the expression of both the NEDD4L gene and protein was inhibited during the induction of colitis by DSS treatment in mice, indicating that DSS-induced IEC ferroptosis may be a potential inducer of the inhibition of NEDD4L expression during colitis. Thus, ferroptosis inducers, including erastin and RSL3, were used to clarify the role of ferroptosis in NEDD4L expression. As shown in Supplemental Figure 4, E and F, erastin and RSL3 significantly inhibited NEDD4L protein expression, suggesting that cell ferroptosis may regulate NEDD4L expression. Furthermore, other classical cell death, epithelial cell pyroptosis induced by TNF-α plus cycloheximide, and staurosporine-induced cell apoptosis inhibited NEDD4L expression, except for insensitive necroptosis in HCT116 cells induced by TNF-α/ SM-164/Z-VAD-FMK (T/S/Z) (32–35) (Supplemental Figure 4, G and H). The key cytokines involved in colitis, such as TNF-α, IL-17A, and IL-1α, were used to test whether DSS-induced downstream cytokines restricted NEDD4L expression. As shown in Supplemental Figure 4, I and J, TNF-α, but not IL-17A or IL-1α, restricted NEDD4L expression in HCT116 cells along with NF-κB p65 subunit phosphorylation, indicating that TNF-α serves as the key mediator for inhibiting NEDD4L expression in IECs. Collectively, our data demonstrate that IEC death induced by DSS, erastin, RSL3, and downstream TNF-α inhibited NEDD4L expression.

Since DSS and ferroptosis inducers directly inhibited NEDD4L expression in HCT116 cells, we tested whether NEDD4L could regulate cell ferroptosis induced by DSS or ferroptosis inducers in vitro. As shown in Supplemental Figure 5, A–E, NEDD4L negatively regulated DSS-induced cell ferroptosis in HCT116 cells in an E3 ligase activity–dependent manner, as assessed by measurement of cell viability, lipid peroxidation, and MDA content. Similar phenotypes were also detected in other cell lines, including SW480 and RKO cells, using a siRNA silencing system (Supplemental Figure 5, F–K). Furthermore, *NEDD4L* deficiency in HCT116, SW480, and RKO cells significantly promoted erastin- or RSL3-induced cell ferroptosis and lipid peroxidation production (Supplemental Figure 5, L–S). Collectively, these data further confirm that NEDD4L negatively regulated cell death and lipid peroxidation production mediated by DSS and ferroptosis inducers in multiple cell lines, in a manner dependent on its E3 ligase activity.

### SLC3A2 is a potential substrate of NEDD4L in DSS-induced colitis.

**Figure 5:**
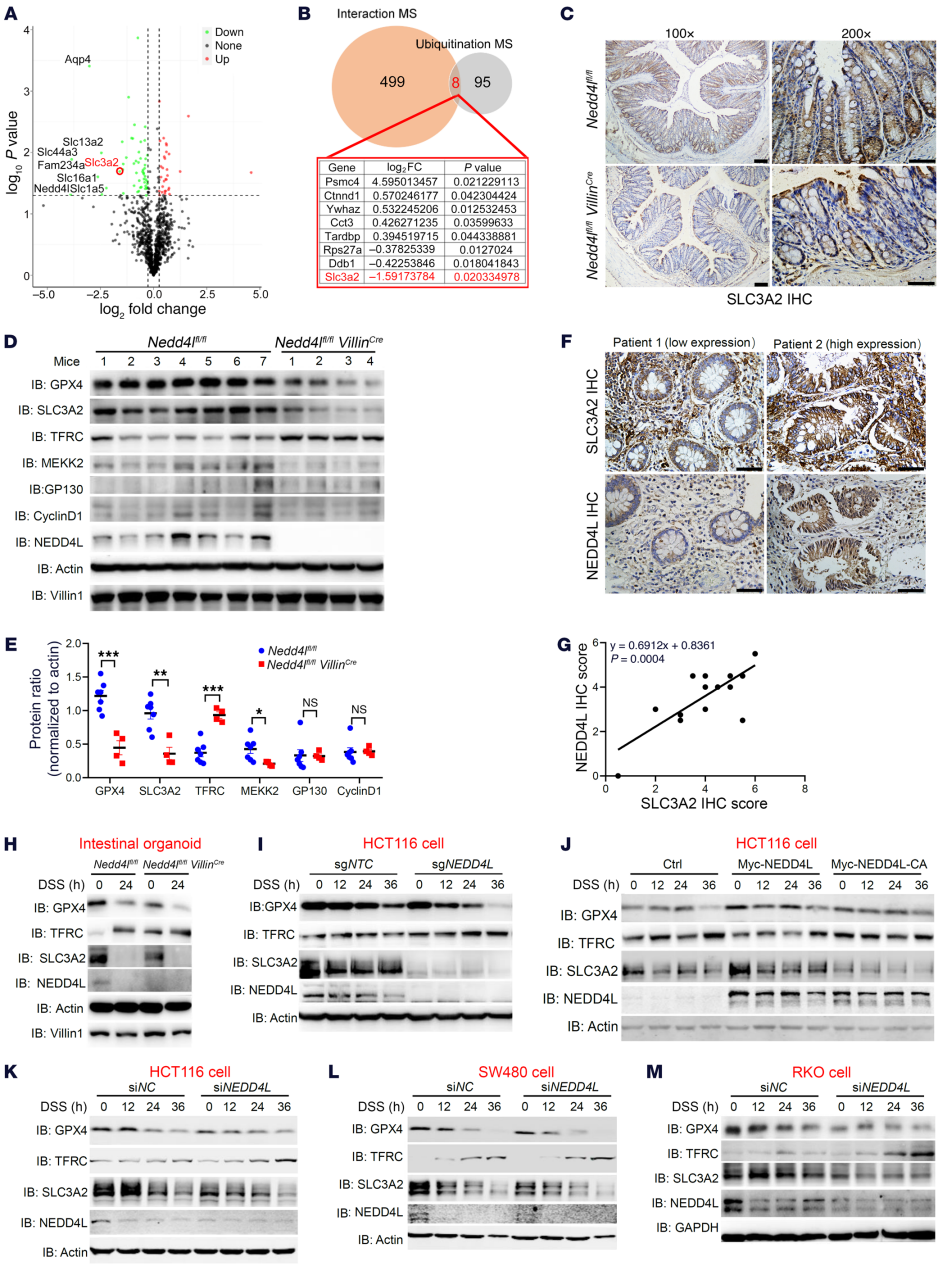
NEDD4L positively regulates SLC3A2 expression. (**A**) Volcano plots of protein abundance fold change based on ubiquitination MS of Figure 4D. (**B**) Venn analysis shows the potential targets of NEDD4L based on interaction MS analysis in HCT116 cells stably expressing FLAG-tagged NEDD4L and ubiquitination MS analysis. The list shows the overlapped targets of NEDD4L in **A** and **B**. (**C**) Representative IHC staining of SLC3A2 from *Nedd4l^fl/fl^ Villin^Cre^* and *Nedd4l^fl/fl^* mice treated with DSS on day 5. Scale bars: 100 μm or 50 μm (amplified sections). (**D** and **E**) Western blotting analysis (**D**) and statistical analysis (**E**) of intensity of the indicated proteins in IECs from *Nedd4l^fl/fl^ Villin* (*n* = 4) and *Nedd4^fl/fl^* (*n* = 7) mice treated as in Figure 3B. (**F** and **G**) Representative IHC staining (**F**) and correlative analysis (**G**) of SLC3A2 and NEDD4L from colonic sections from CD patients (*n* = 13). Scale bars: 50 μm. (**H**) Immunoblot analysis of the indicated proteins in small-intestinal organoids isolated and cultured from crypts of *Nedd4l^fl/fl^ Villin^Cre^* and *Nedd4l^fl/fl^* mice, with 0.5% DSS treatment for the indicated times. (**I** and **J**) *NEDD4L-*KO (sg*NEDD4L*) and negative control (sg*NTC*) HCT116 cell lines, or HCT116 cells transfected with Myc-tagged NEDD4L, Myc-tagged NEDD4L-C942A (Myc-NEDD4L-CA), or Myc-tagged null control plasmids (Ctrl), were treated with 2% DSS for the indicated times and then subjected to immunoblot analysis of the indicated proteins. (**K**–**M**) Immunoblot analysis of the indicated proteins in HCT116 cells (**K**), SW480 cells (**L**), and RKO cells (**M**) transfected with siRNA targeted to NEDD4L (si*NEDD4L*) or negative control (si*NC*) and treated as in **I**. Data represent mean ± SEM from at least 2 independent experiments. Each dot represents an independent sample. *P < 0.05; **P < 0.01; ***P < 0.001. Statistical analysis was performed using a 2-tailed Student’s *t* test in **E**, and a Pearson’s correlation test in **G**.

Based on the quantitative ubiquitination MS analysis, SLC3A2, a transmembrane protein, which forms the key glutamine/cystine transporter with SLC7A11 and consequently participates in ferroptosis, was identified as one of the most remarkably ubiquitinylated substrates and was significantly downregulated in *Nedd4l^fl/fl^ Villin^Cre^* IECs compared with that in *Nedd4l^fl/fl^* IECs after DSS challenge. Nevertheless, the fold change of SLC3A2 analyzed by ubiquitination MS was inhibited as a result of the reduced NEDD4L expression upon DSS treatment in comparison with untreated mice (Figure 5, A and B, and Supplemental Figure 6, A and B). The interaction MS analysis in HCT116 cells stably expressing FLAG-NEDD4L indicated that NEDD4L interacted with SLC3A2 (Figure 5B and Supplemental Figure 6C). Based on the combined analysis of quantitative ubiquitination MS and interaction MS, we hypothesized that NEDD4L might interact with SLC3A2 and regulate its ubiquitination, triggering IEC ferroptosis and aggravating DSS-induced colitis. Consistently, the protein expression of SLC3A2 was significantly downregulated in IECs of *Nedd4l^fl/fl^ Villin^Cre^* mice compared with that in *Nedd4l^fl/fl^* mice (Supplemental Figure 6D), whereas *Nedd4l* deficiency in IECs had no effects on the protein expressions of GP130 and MEKK2, which have been identified to be potential substrates of NEDD4L in other cells (28, 29). Furthermore, upon DSS treatment, the expression of SLC3A2 was also downregulated in IECs of *Nedd4l^fl/fl^ Villin^Cre^* mice compared with that in *Nedd4l^fl/fl^* mice (Figure 5C). Based on the ubiquitination MS analysis, we found that NEDD4L protein abundance was positively correlated with SLC3A2 protein abundance, further indicating the probability of SLC3A2 as the potential substrate of NEDD4L (Supplemental Figure 6E). It has been reported that SLC3A2 regulates the expression of cyclin D1 in IECs to participate in mouse colitis (36). However, we did not observe any difference in the gene expressions of *Cyclind1* and *Slc3a2* in *Nedd4l^fl/fl^ Villin^Cre^* and *Nedd4l^fl/fl^* mice (Supplemental Figure 6F). In addition, we revealed that *Nedd4l* deficiency in IECs restricted SLC3A2 and GPX4 protein expression (Figure 5, C–E). DSS treatment significantly inhibited the protein expression levels of GPX4, SLC3A2, and NEDD4L. Furthermore, the protein expression levels of both NEDD4L and GPX4 were positively correlated with SLC3A2 in IECs upon DSS treatment (Supplemental Figure 6, G–I). Importantly, the protein expression level of NEDD4L in patients with IBDs was positively correlated with SLC3A2 (Figure 5, F and G).

**Figure 6:**
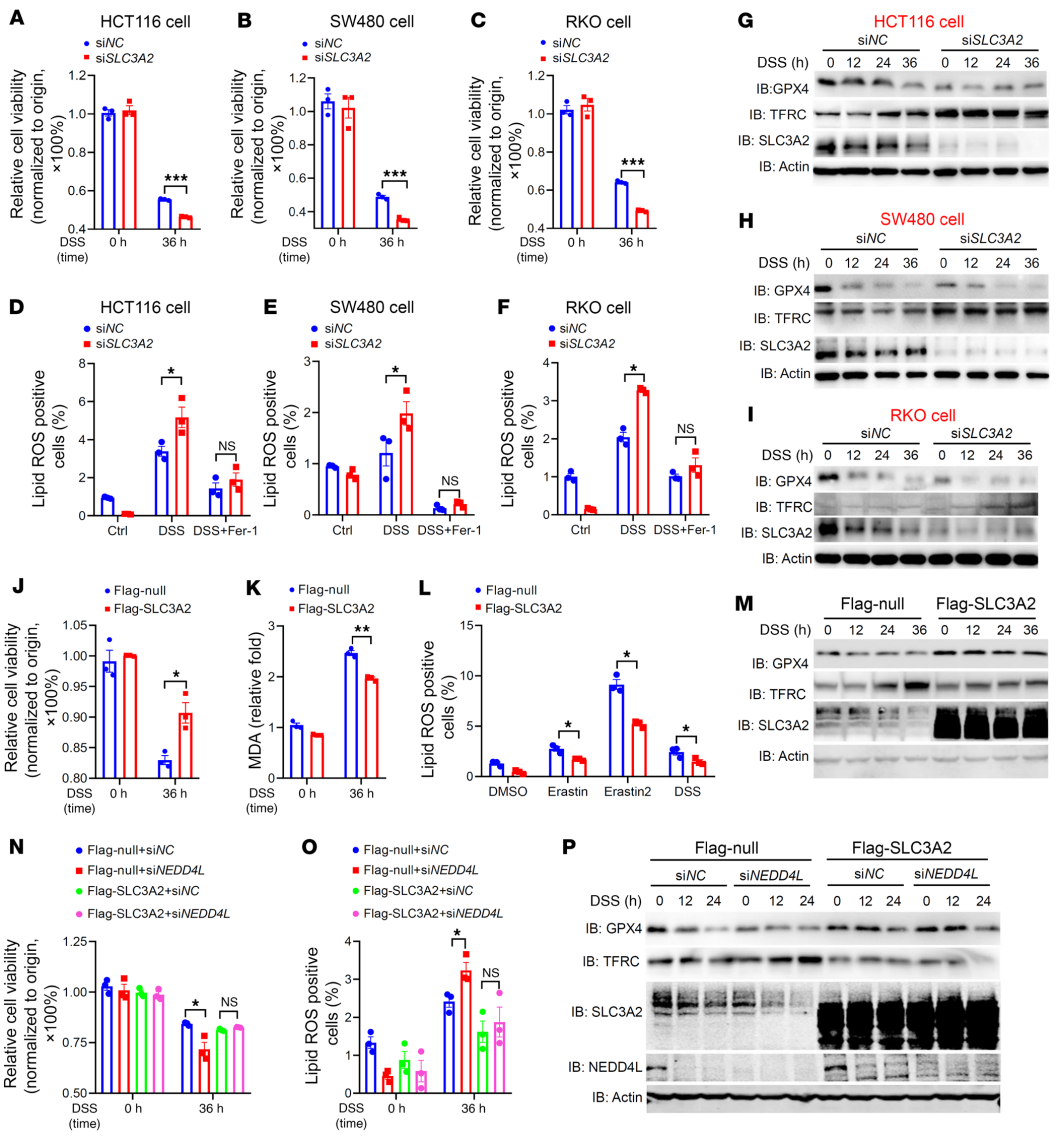
SLC3A2 negatively regulates ferroptosis. (**A**–**C**) The multiple cell lines, including HCT116 cells (**A**), SW480 cells (**B**), and RKO cells (**C**), were transfected with siRNA targeted to *SLC3A2* (si*SLC3A2*) or negative control (si*NC*). The cells were treated with 2% DSS for the indicated times and then subjected to CCK8 assay. (**D**–**F**) The multiple cell lines were treated as in **A**–**C** with or without Fer-1 (2 μM) treatment. The cells were then subjected to flow cytometry analysis of BODIPY C11 staining to measure lipid peroxidation production. (**G**–**I**) The multiple cell lines were treated as in **A**–**C** for the indicated times and then subjected to immunoblot analysis of the indicated proteins. (**J**–**M**) FLAG-tagged SLC3A2 or FLAG-tagged null control plasmids were overexpressed in HCT116 cells. The cells were treated with 2% DSS or indicated inducers for the stated times, and then subjected to CCK8 assay (**J**), MDA assay (**K**), flow cytometry analysis of BODIPY C11 staining (**L**), and immunoblot analysis of the indicated proteins (**M**). (**N**–**P**) HCT116 cells were transfected with oligonucleotides specific for siRNA negative control (si*NC*) or NEDD4L (si*NEDD4L*), and then FLAG-tagged SLC3A2 or FLAG-tagged null control plasmid was overexpressed in the cells. The cells were treated with 2% DSS for the indicated times and then subjected to CCK8 assay (**N**) and lipid peroxidation (**O**). (**P**) Immunoblot analysis of the indicated proteins. Data represent mean ± SEM from at least 2 independent experiments. Each dot represents an independent sample. *P < 0.05; **P < 0.01; ***P < 0.001. Statistical analysis was performed using a 2-tailed Student’s *t* test in **A**–**F**, **J**–**L**, **N**, and **O**.

NEDD4L KO in intestinal organoids and HCT116 cells impaired DSS-induced SLC3A2 and GPX4 expression but increased TFRC expression, enhancing cell ferroptosis (Figure 5, H and I). NEDD4L positively regulated SLC3A2 and GPX4 protein expression in HCT116 cells in an E3 ubiquitin ligase activity–dependent manner (Figure 6J). Similar results were observed in a multiple of DSS-, erastin-, or RSL3-treated intestinal cell lines, such as HCT116, SW480, and RKO cells, using a siRNA silencing system (Figure 5, K–M, and Supplemental Figure 6, J–M).

As a potential substrate of NEDD4L in ferroptosis signaling, SLC3A2 was poorly studied (11). Therefore, we determined whether SLC3A2 could regulate cell ferroptosis and signaling transduction mediated by DSS or ferroptosis inducers. As shown in Figure 6, A–I, and Supplemental Figure 7, A–I, silencing of endogenous *SLC3A2* significantly promoted cell death and lipid peroxidation production induced by DSS and ferroptosis inducers. Additionally, silencing of endogenous *SLC3A2* inhibited GPX4 expression but enhanced TFRC expression after DSS or ferroptosis inducer treatment in comparison with cells transfected with scramble siRNA (si*NC*). Overexpression of exogenous *SLC3A2* in HCT116 cells inhibited DSS-induced cell death and production of lipid peroxidation by upregulating GPX4 expression (Figure 6, J–M), indicating that SLC3A2 negatively regulated cell ferroptosis mediated by DSS and ferroptosis inducers in vitro. Furthermore, overexpression of the exogenous *SLC3A2* eliminated the difference in DSS-induced cell death, production of lipid peroxidation, and protein expression levels of GPX4 and TFRC between *NEDD4L*-silenced and scramble siRNA (si*NC*)–transfected HCT116 cells (Figure 6, N–P). Collectively, these data suggest that NEDD4L regulated DSS-induced cell ferroptosis through the SLC3A2/GPX4 axis.

### NEDD4L mediates SLC3A2 ubiquitination.

**Figure 7:**
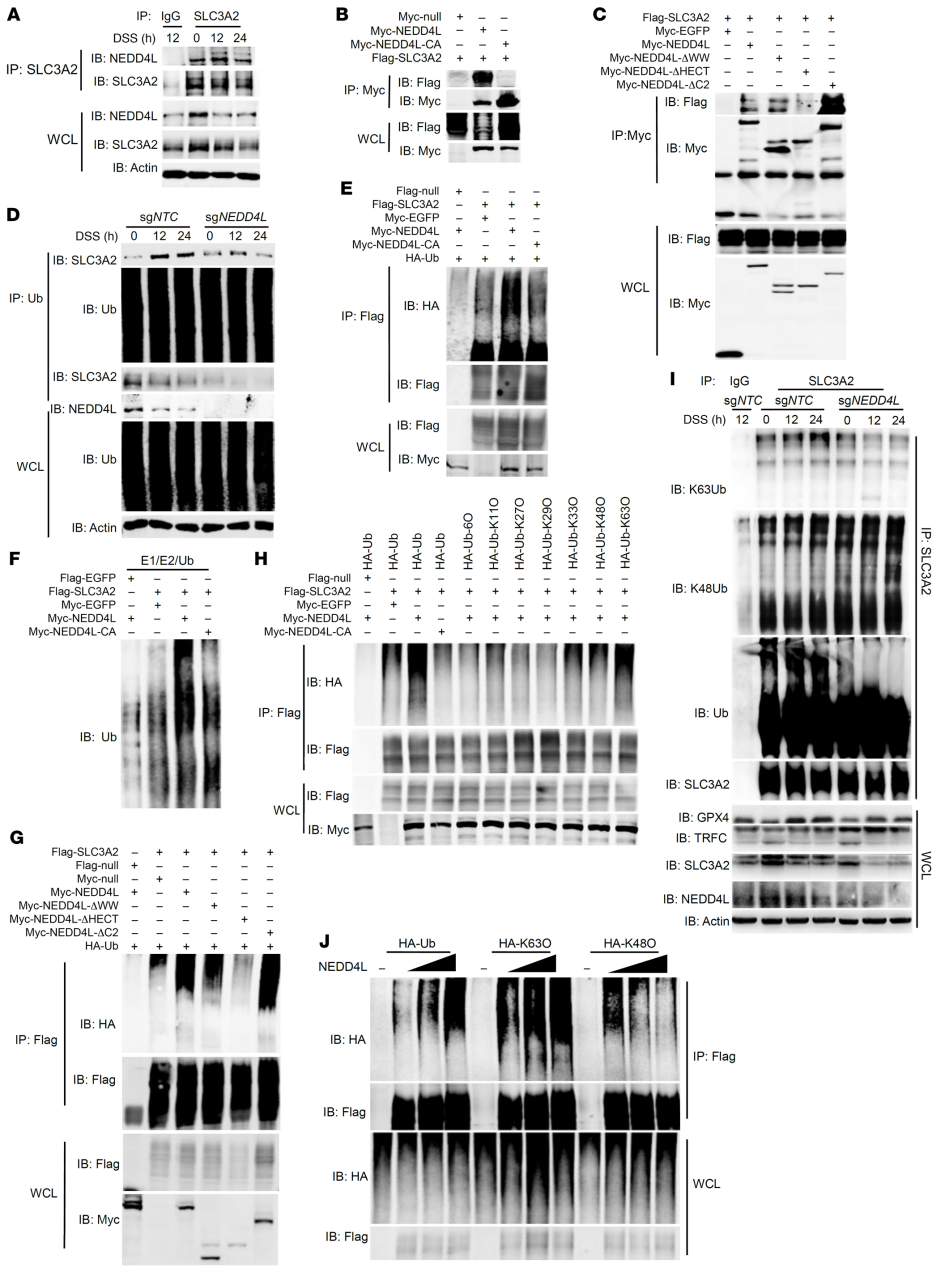
NEDD4L ubiquitinates SLC3A2. (**A**) Immunoblot analysis of NEDD4L and SLC3A2 coimmunoprecipitated with anti-SLC3A2 antibody from lysates of HCT116 cells treated with 2% DSS for the indicated times. (**B** and **C**) Immunoblot analysis of Myc-tagged proteins and FLAG-tagged SLC3A2 coimmunoprecipitated with anti-Myc antibody from lysates of HEK293T cells cotransfected with indicated plasmids. WCL, whole-cell lysate. (**D**) Immunoblot analysis of NEDD4L, SLC3A2, and Ub, which were coimmunoprecipitated with anti-Ub antibody from lysates of NEDD4L (sg*NEDD4L*) or negative control (sg*NTC*) KO HCT116 cells treated with 2% DSS for the indicated times. (**E**) Immunoblot analysis of total ubiquitination of FLAG-tagged SLC3A2 following coimmunoprecipitation with anti-FLAG antibody from lysates of HEK293T cells cotransfected with indicated plasmids. (**F**) Immunoblot analysis of Ub-linked FLAG-tagged EGFP or SLC3A2 incubated with Myc-tagged NEDD4L, Myc-tagged NEDD4L-C942A (CA), or Myc-tagged EGFP recombinant protein in the presence of the full complement of ubiquitination reaction components, including E1, E2, Ub, and ATP, in vitro. (**G** and **H**) Immunoblot analysis of ubiquitination of FLAG-tagged SLC3A2 following coimmunoprecipitation of SLC3A2 with anti-FLAG antibody from lysates of HEK293T cells cotransfected with indicated plasmids. (**I**) Immunoblot analysis of K63Ub, K48Ub, Ub, GPX4, TFRC, SLC3A2, NEDD4L, and actin, which were coimmunoprecipitated with anti-SLC3A2 antibody from lysates of NEDD4L (sg*NEDD4L*) or negative control (sg*NTC*) KO HCT116 cells treated with 2% DSS for the indicated times and pretreated with 20 μM MG132 for 6 hours. (**J**) Immunoblot analysis of total ubiquitination of FLAG-tagged SLC3A2 following coimmunoprecipitation of SLC3A2 with anti-FLAG antibody from lysates of HEK293T cells cotransfected with indicated plasmids.

**Figure 8:**
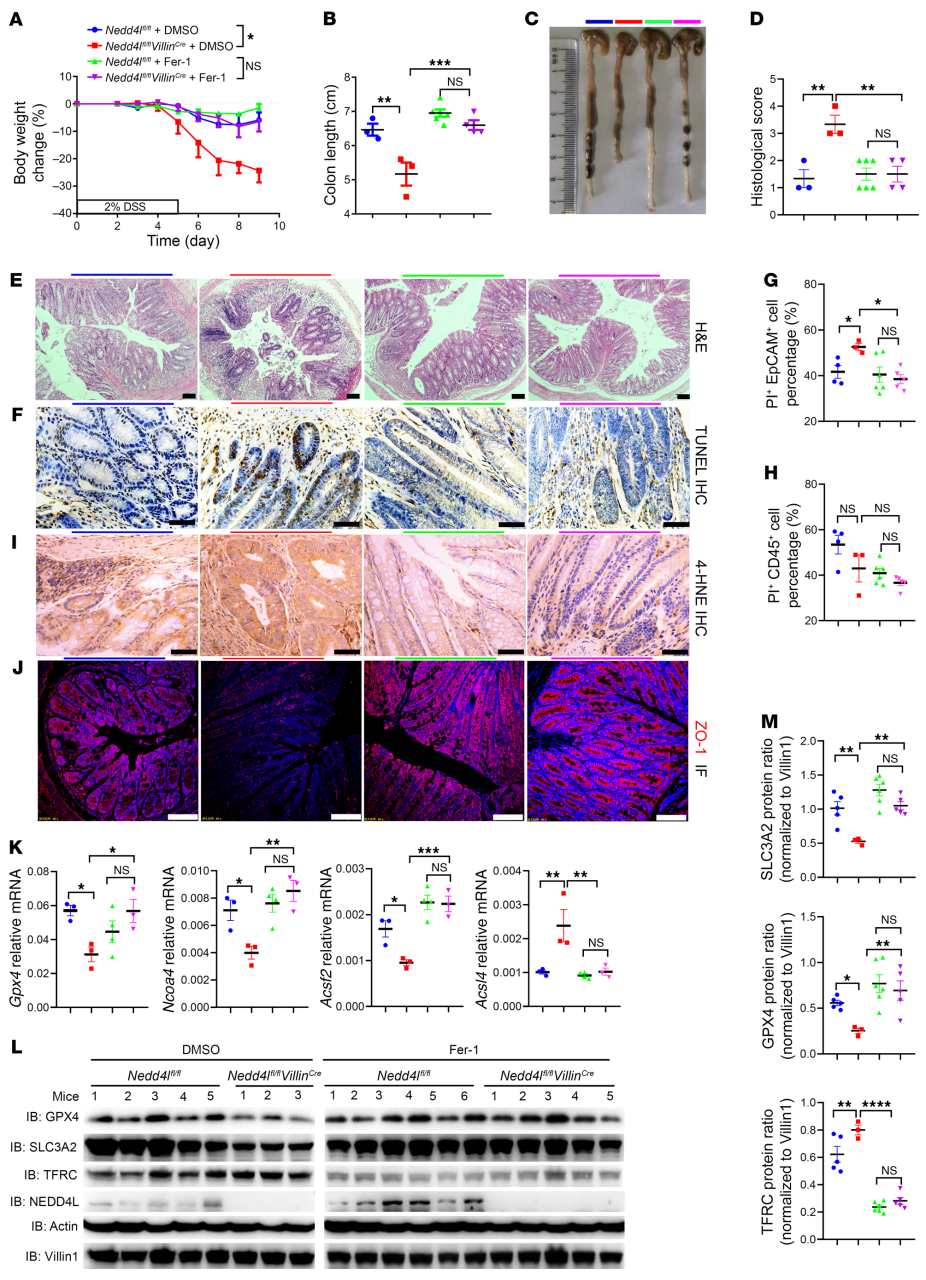
NEDD4L regulates DSS-induced colitis through ferroptosis. *Nedd4l^fl/fl^ Villin^Cre^* and *Nedd4l^fl/fl^* mice pretreated with Fer-1 (5 μM/kg) or DMSO were given 2% DSS for 5 days, and on the 9th day the mice were sacrificed for collection of colonic tissues and IECs. *Nedd4l^fl/fl^* + DMSO *n* = 3, *Nedd4l^fl/fl^ Villin^Cre^* + DMSO *n* = 4, *Nedd4l^fl/fl^* + Fer-1 *n* = 6, *Nedd4l^fl/fl^ Villin^Cre^* + Fer-1 *n* = 4. (**A**–**F**) Body weight change (**A**), colon length (**B**), gross morphology images (**C**), histological score (**D**), representative H&E staining (**E**), and TUNEL staining of the colon sections (**F**) from the indicated mice. (**G**–**J**) In a separate experiment, IECs and colon tissues from mice treated as in **A** were subjected to flow cytometry analysis of EpCAM, CD45, and propidium iodide (PI) staining (**G** and **H**), 4-HNE IHC staining (**I**), and ZO-1 IF staining (**J**). (**K**–**M**) qPCR analysis (**K**), Western blotting analysis (**L**), and protein intensity analysis (**M**) of the indicated proteins of IECs treated as in **A**. *Nedd4l^fl/fl^* + DMSO *n* = 3–5, *Nedd4l^fl/fl^ Villin^Cre^* + DMSO *n* = 3, *Nedd4l^fl/fl^* + Fer-1 *n* = 4–6, *Nedd4l^fl/fl^ Villin^Cre^* + Fer-1 *n* = 3–5, as indicated in the figure. Scale bars: 50 μm. Data represent mean ± SEM from at least 2 independent experiments. Each dot represents an independent sample. *P < 0.05; **P < 0.01; ***P < 0.001; ****P < 0.0001. Statistical analysis was performed using a 2-way ANOVA test in **A**, and 1-way ANOVA with multiple comparisons **B**, **D**, **G**, **H**, **K**, and **M**.

To determine the mechanism through which NEDD4L orchestrates SLC3A2 protein expression, we investigated the interaction between NEDD4L and SLC3A2 in HCT116 and HEK293T cells. As shown in Figure 7, A and B, NEDD4L interacted dynamically with SLC3A2 upon DSS treatment, peaking at 12 hours. The E3 ligase activity mutant of NEDD4L (NEDD4L-C942A or NEDD4L-CA) abolished this interaction. To map the domains required for NEDD4L to interact with SLC3A2, we constructed a series of plasmids expressing WT NEDD4L or mutant NEDD4L, in which the C2, WW, or HECT domain was deleted (ΔC2, ΔWW, or ΔHECT, respectively). As shown in Figure 7C, the deletion of the HECT domain, but not of the C2 and WW domain, disrupted the interaction between NEDD4L and SLC3A2, demonstrating that the HECT domain was necessary for NEDD4L to bind SLC3A2. As an E3 ubiquitin ligase, NEDD4L might regulate the stability of the SLC3A2 protein by mediating its ubiquitination. Firstly, we used the ubiquitin (Ub) antibody to immunoprecipitate endogenous Ub to compare the amount of poly-Ub-linked SLC3A2 in WT (sg*NTC*) and NEDD4L-KO (sg*NEDD4L*) HCT116 cells. As shown in Figure 7D, NEDD4L KO in HCT116 cells impaired the poly-Ub-linked SLC3A2 upon DSS treatment, consistent with the phenotype observed in our ubiquitination MS in IECs. Then, we performed ubiquitination assays in HEK293T cells. As shown in Figure 7E and Supplemental Figure 8A, NEDD4L positively regulated the polyubiquitination of SLC3A2. Furthermore, in vitro cell-free ubiquitination assays demonstrated that it was the WT NEDD4L protein, but not the NEDD4L-C942A protein, that directly promoted the polyubiquitination of SLC3A2 (Figure 7F). Following MG132 treatment, but not bafilomycin A1 treatment, the expression of SLC3A2 in WT NEDD4L–transfected cells was reduced to a level comparable to that in control or NEDD4L-CA mutant–transfected HCT116 cells, suggesting that NEDD4L regulated the stability of SLC3A2 protein by mediating SLC3A2 ubiquitination in a proteasome-dependent manner (Supplemental Figure 8B). Notably, NEDD4L overexpression in HCT116 cells markedly enhanced the protein stability of SLC3A2 compared with that in NEDD4L-C942A– or control-transfected cells (Supplemental Figure 8C). NEDD4L-ΔHECT completely lost the ability to mediate SLC3A2 ubiquitination (Figure 7G), suggesting that the HECT domain of NEDD4L was critical for its interaction with and ubiquitination of SLC3A2. Furthermore, NEDD4L mainly promoted Lys-63 (K63O)–linked polyubiquitination of SLC3A2 (Figure 7H), which is consistent with the well-established notion that the C-terminal amino acids determine the ubiquitin chain specificity of the HECT-type E3 ligases and NEDD4 family ligases, including NEDD4L, which exhibit strict specificity toward K63 linkages (37). NEDD4L KO markedly impaired DSS-induced K63-linked polyubiquitination of SLC3A2, but enhanced K48-linked polyubiquitination of SLC3A2, resulting in reduced SLC3A2 protein expression in comparison with sg*NTC* HCT116 cells (Figure 7I). Furthermore, NEDD4L promoted K63-linked polyubiquitination of SLC3A2 in a dosage-dependent manner and inhibited the K48-linked polyubiquitination of SLC3A2 in HEK293T cells (Figure 8J). We also found that SLC3A2 interacted with GPX4. However, NEDD4L neither interacted with nor ubiquitylated GPX4 (Supplemental Figure 8, D and E). These data suggest that NEDD4L mediated the K63-linked polyubiquitination of SLC3A2, but not of GPX4.

### Nedd4l deficiency promotes colitis pathogenesis via ferroptosis in mice.

To further determine whether NEDD4L regulates colitis through the ferroptosis pathway, colonic tissues from *Nedd4l^fl/fl^ Villin^Cre^* and *Nedd4l^fl/fl^* mice treated with DSS were subjected to RNA sequencing to explore the underlying mechanisms. KEGG analysis revealed that cytokine–cytokine receptor interaction and IL-17 signaling pathway were the top 2 pathways upregulated in colonic tissues from *Nedd4l^fl/fl^ Villin^Cre^* mice compared with *Nedd4l^fl/fl^* mice (Supplemental Figure 9A). GO analysis showed that cell-intrinsic apoptotic signaling and regulation of the hydrogen peroxide metabolic process were significantly upregulated in colonic tissues from *Nedd4l^fl/fl^ Villin^Cre^* mice compared with *Nedd4l^fl/fl^* mice (Supplemental Figure 9B), suggesting that cell death and peroxidation may be involved in NEDD4L-mediated colitis. Previous studies have shown that NEDD4L regulated IL-17–induced inflammatory response through MEKK2 (28). Since IL-17R signaling can affect IEC homeostasis, differentiation, and tumor development (38–40), we tested whether NEDD4L regulates DSS-induced colitis through IL-17R signaling by using an IL-17 neutralizing antibody. As shown in Supplemental Figure 9, C–F, the IL-17 neutralizing antibody treatment successfully inhibited DSS-mediated colitis in WT mice but did not eliminate the colitis phenotype difference induced by *Nedd4l* deficiency. Although Syk is known to be a target for NEDD4L in mast cells (41), continual intraperitoneal (i.p.) injection of a Syk-specific inhibitor, BAY 61-3606, during colitis induction did not eliminate the colitis phenotype difference between *Nedd4l^fl/fl^ Villin^Cre^* and *Nedd4l^fl/fl^* mice (Supplemental Figure 9, G–J). However, treatment with a lipid peroxidation scavenger, *N*-acetylcysteine (NAC), significantly attenuated the development of colitis in *Nedd4l^fl/fl^* *Villin^Cre^* mice. More importantly, NAC treatment rescued the colitis phenotype in *Nedd4l^fl/fl^ Villin^Cre^* mice to a level comparable to that in *Nedd4l^fl/fl^* mice (Supplemental Figure 9, K–N).

To further explore whether NEDD4L regulates colitis via ferroptosis, a ferroptosis-specific inhibitor, ferrostatin-1 (Fer-1), was continually injected i.p. during DSS-induced colitis in *Nedd4l^fl/fl^ Villin^Cre^* and *Nedd4l^fl/fl^* mice. As shown in Figure 8, A–J, and Supplemental Figure 10, A and B, Fer-1 markedly rescued the colitis phenotype in DSS-induced *Nedd4l^fl/fl^ Villin^Cre^* mice to levels comparable to those in Fer-1–treated *Nedd4l^fl/fl^* mice, as characterized by reduced diarrhea and rectal bleeding, decreased colon shortening, less epithelial damage, decreased crypt architecture disruption, decreased epithelial cell death, reduced lipid peroxidation production, and decreased inflammatory cytokines, but increased tight junctions. Furthermore, continual i.p. injection of Fer-1 during the induction of colitis eliminated the difference in colitis phenotype between *Nedd4l^fl/fl^ Villin^Cre^* and *Nedd4l^fl/fl^* mice. The difference in the expression of ferroptosis-related genes (including *Gpx4*, nuclear receptor coactivator 4 [*Ncoa4*], acyl-CoA synthetase family member 2 [*Acsf2*], and acyl-CoA synthetase long chain family member 4 [*Acsl4*]) and proteins (including GPX4, SLC3A2, and TFRC) between *Nedd4l^fl/fl^ Villin^Cre^* and *Nedd4l^fl/fl^* mice was eliminated by the treatment with Fer-1 (Figure 8, K–M). Additionally, treatment with another ferroptosis inhibitor, deferoxamine mesylate (DFOM; a ferric ion depletion reagent), during the DSS administration eliminated the colitis phenotype difference in mice (Supplemental Figure 10, C–K). These data suggest that *Nedd4l* deficiency in IECs promoted the pathogenesis of colitis in a ferroptosis-dependent manner.

### Gut microbiota is involved in NEDD4L-regulated colitis.

The gut microbiota is critical for maintaining gut homeostasis. To further evaluate whether the exacerbated colitis in *Nedd4l*-deficient mice compared with control littermates is microbiota dependent, we cohoused the *Nedd4l*-deficient mice with control littermates for 2 weeks before DSS administration. As shown in Supplemental Figure 11, A–F, cohousing eliminated the development of more severe DSS-induced colitis in *Nedd4l-*deficient mice compared with cohoused control littermates, indicating that NEDD4L protects against colitis in a manner dependent on the gut microbiota. To demonstrate how the microbiota regulates DSS-induced colitis in mice, feces from *Nedd4l^fl/fl^ Villin^Cre^* mice and control littermates, treated or not treated with DSS, were collected and then subjected to 16S rDNA sequencing. As shown in Supplemental Figure 11G, the abundance of *Akkermansia* was markedly increased, while the abundances of *Bifidobacterium* and *Lactobacillus* were markedly diminished, in *Nedd4l^fl/fl^ Villin^Cre^* mice compared with *Nedd4l^fl/fl^* mice after administration of DSS, with similar abundances in untreated mice. As important commensal intestinal bacteria, *Akkermansia*, *Bifidobacterium*, and *Lactobacillus* play pivotal roles in maintaining intestinal homeostasis (2). However, an abnormally increased abundance of *Akkermansia* could promote the degradation of intestinal mucin, thus exacerbating colitis in mice (42), which is consistent with our phenotype that *Nedd4l^fl/fl^ Villin^Cre^* mice exhibited less intestinal mucin production after DSS treatment visualized by AB-PAS staining of the colon sections (Supplemental Figure 11H). To further investigate the involvement of gut microbiota in NEDD4L-regulated colitis, antimicrobial peptides of the small intestine were detected in untreated and DSS-treated *Nedd4l^fl/fl^ Villin^Cre^* mice and *Nedd4l^fl/fl^* mice. As shown in Supplemental Figure 11, I and J, *Nedd4l* deficiency in mice initially had no effect on antimicrobial peptide expression without DSS treatment, such as angiogenin, ribonuclease A family, member 4 (*Ang4*); defensin, alpha, 29 (*Defa-rs1*); and defensin, alpha, 20 (*Defa20*). DSS treatment resulted in IEC damage along with decreased antimicrobial peptide gene expression patterns. Furthermore, *Nedd4l* deficiency in IECs significantly impaired antimicrobial peptide expression in *Nedd4l^fl/fl^ Villin^Cre^* mice compared with *Nedd4l^fl/fl^* mice, suggesting that a much stronger impact, such as IEC death, plays a critical role during DSS-induced colitis. Thus, single-housed *Nedd4l^fl/fl^ Villin^Cre^* and *Nedd4l^fl/fl^* mice were gavaged with *Bifidobacterium* and *Lactobacillus* (*Bif&Lac*; 1 × 10^8^ CFU per mouse daily) during the induction of colitis. Interestingly, as shown in Supplemental Figure 11, K–N, oral administration of *Bifidobacterium* and *Lactobacillus* significantly restricted colitis development in both *Nedd4l^fl/fl^ Villin^Cre^* mice and *Nedd4l^fl/fl^* mice, characterized by a lower degree of the inflammatory syndrome and stronger mucus secretion ability compared with DSS-treated single-housed *Nedd4l^fl/fl^ Villin^Cre^* mice without bacteria gavage, indicating that the intestinal microbiota was involved in NEDD4L-regulated colitis, particularly *Bifidobacterium* and *Lactobacillus*. The IEC samples isolated from the mice with bacteria gavage revealed that the administration of microbiota significantly promoted GPX4 and SLC3A2 expression but impaired TFRC expression, thus eliminating the signaling difference between *Nedd4l^fl/fl^ Villin^Cre^* and *Nedd4l^fl/fl^* mice (Supplemental Figure 11, O and P), indicating a protective role of gut microbiota in inhibiting ferroptosis through GPX4 (43).

### Nedd4l deficiency promotes the pathogenesis of CAC in mice.

**Figure 9:**
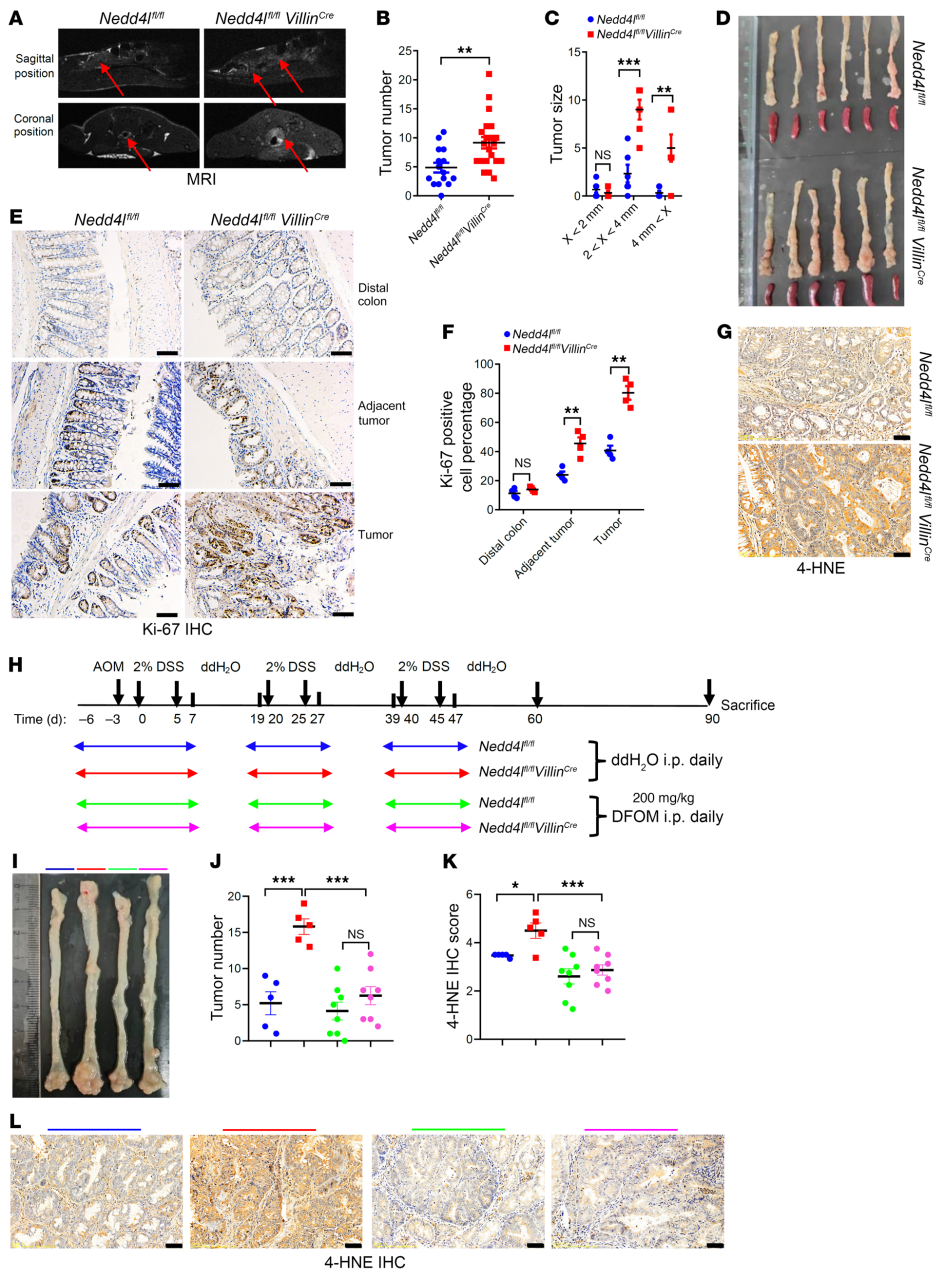
*Nedd4l* deficiency in IECs promotes AOM/DSS–induced CRC in mice. (**A**) MRI images of *Nedd4l^fl/fl^ Villin^Cre^* and *Nedd4l^fl/fl^* mice treated with AOM/DSS for 90 days. (**B**–**D**) Tumor numbers (*Nedd4l^fl/fl^ n* = 15, *Nedd4l^fl/fl^ Villin^Cre^ n* = 21) (**B**), tumor size (*n* = 6 per group) (**C**), and representative morphology images of colons (**D**) from the AOM/DSS–treated mice on day 90. (**E**–**G**) Representative IHC staining of sections from the tumor, adjacent tumor, and distal normal tissues of AOM/DSS–treated *Nedd4l^fl/fl^ Villin^Cre^* and *Nedd4l^fl/fl^* mice with anti-Ki67 antibody (**E**) and anti–4-HNE antibody (**G**), and statistical analysis of Ki67^+^ cells (**F**) according to **E** (*n* = 4 per group). (**H**) Schematic diagram of the treatment plan for AOM/DSS–treated *Nedd4l^fl/fl^ Villin^Cre^* and *Nedd4l^fl/fl^* mice with ddH_2_O or DFOM. (**I**–**L**) Representative morphology images of colons (**I**), tumor numbers (**J**), statistical analysis of 4-HNE IHC staining score (**K**), and representative images of 4-HNE IHC staining (**L**) from the treated mice as in **I**. *Nedd4l^fl/fl^* + ddH_2_O *n* = 5, *Nedd4l^fl/fl^ Villin^Cre^* + ddH_2_O *n* = 5, *Nedd4l^fl/fl^* + DFOM *n* = 8, *Nedd4l^fl/fl^ Villin^Cre^* + DFOM *n* = 8. Scale bars: 50 μm. Data represent mean ± SEM from at least 2 independent experiments. Each dot represents an independent sample. *P < 0.05; **P < 0.01; ***P < 0.001. Statistical analysis was performed using a 2-tailed Student’s *t* test in **B**, **C**, and **F**, and 1-way ANOVA with multiple comparisons in **J** and **K**.

The azoxymethane (AOM)/DSS–induced colitis-associated colorectal cancer (CAC) model in mice has been widely used for research on inflammation-related cancer in mice, as mice with more severe inflammation are more likely to develop CRC (44, 45). Therefore, we further explored the regulatory role of NEDD4L in CAC using mice with global *Nedd4l* deficiency and *Nedd4l^fl/fl^ Villin^Cre^* mice. In vivo, magnetic resonance imaging (MRI) analysis revealed a marked increase in colon distension in *Nedd4l^fl/fl^ Villin^Cre^* mice in both axial and coronal images, and a higher number of tumors in the colons of *Nedd4l^fl/fl^ Villin^Cre^* mice compared with WT mice on day 90 (Figure 9A). As shown in Figure 9, A–D, and Supplemental Figure 12, A–C, *Nedd4l*-deficient mice were more susceptible to cancer. Compared with their WT littermates, we found higher levels of Ki67^+^ cells per crypt in the adjacent tumor and tumor tissues from *Nedd4l^+/–^* and *Nedd4l^fl/fl^ Villin^Cre^* mice following AOM/DSS treatment (Figure 9, E and F, and Supplemental Figure 12, D and E), as well as increased lipid peroxidation production in tumor tissues of *Nedd4l^fl/fl^ Villin^Cre^* mice (Figure 9G). Since NEDD4L regulates IEC inflammation through ferroptosis signaling, we hypothesized that NEDD4L may regulate CAC through ferroptosis signaling. To test this hypothesis, a ferroptosis inhibitor, DFOM, was injected i.p. during DSS treatment as indicated in Figure 9H, to inhibit the inflammatory response. As shown in Figure 9, I–L, DFOM treatment significantly inhibited AOM/DSS–induced tumor formation and lipid peroxidation in *Nedd4l^fl/fl^ Villin^Cre^* mice compared with the ddH_2_O-treated control mice, and further eliminated the phenotype difference between *Nedd4l^fl/fl^ Villin^Cre^* mice and *Nedd4l^fl/fl^* mice, suggesting that NEDD4L regulated CAC through ferroptosis signaling.

**Figure 10:**
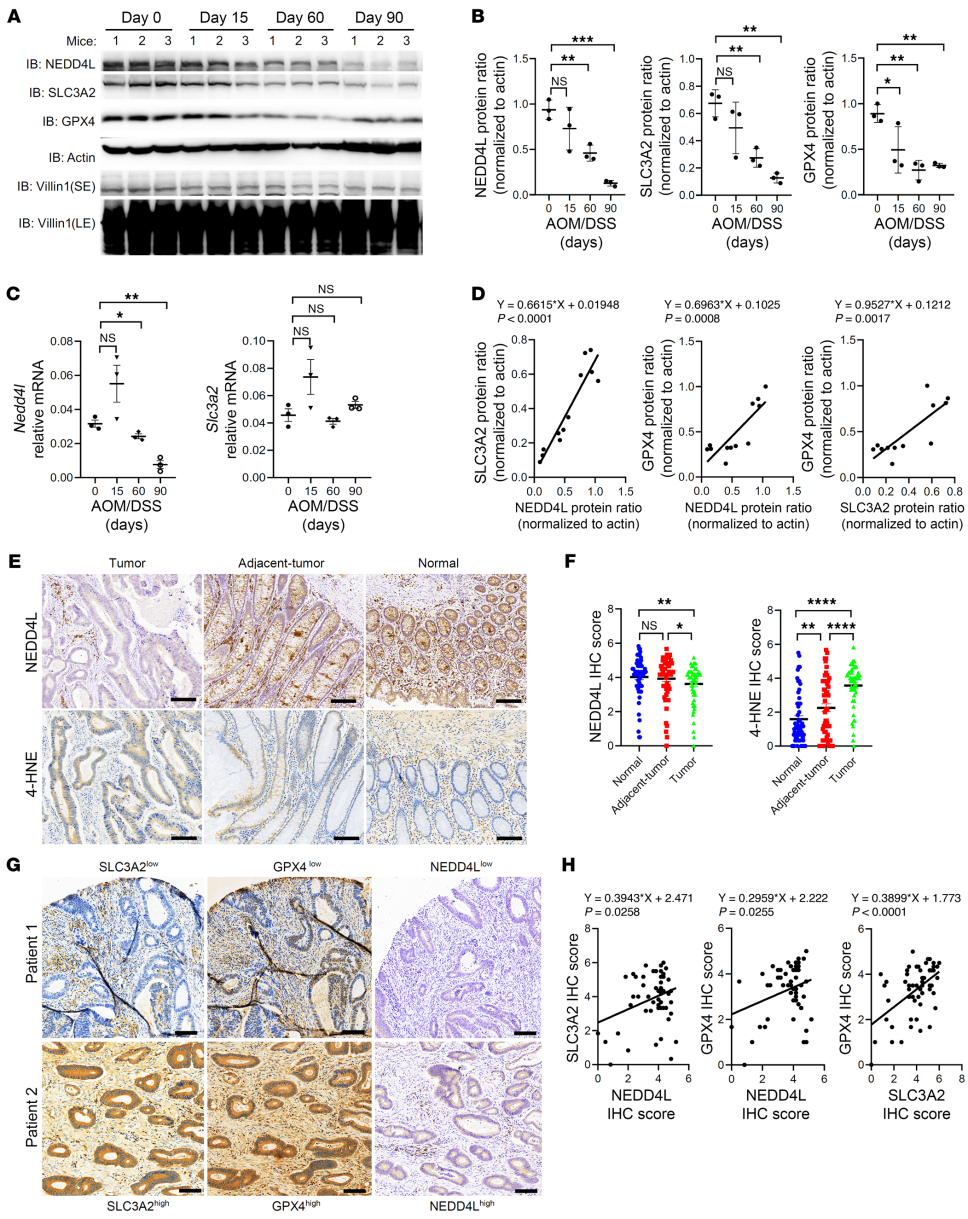
Expression of NEDD4L is significantly downregulated in IECs of patients and mice with CRC. (**A**–**D**) WT mice were treated with AOM/DSS, and IECs (on day 0, day 15, and day 60) and tumor nodes (on day 90) were collected for immunoblot analysis (**A**), protein intensity analysis (**B**), qPCR analysis (**C**), and correlative analysis of the indicated proteins (**D**). *n* = 3 per group. (**E** and **F**) Representative NEDD4L and 4-HNE IHC staining of sections from the tumor, adjacent tumor, and distal normal tissues of patients with CRC (**E**), and statistical analysis of NEDD4L and 4-HNE IHC staining intensity (**F**) according to **E**. *n* = 55. (**G** and **H**) Representative SLC3A2, GPX4, and NEDD4L IHC staining sections from the tumor tissues of patients with CRC (**G**), and correlative analysis between SLC3A2, GPX4, and NEDD4L IHC staining intensity score (*n* = 55) (**H**). Scale bars: 50 μm. Data represent mean ± SEM from at least 2 independent experiments. Each dot represents an independent sample. *P < 0.05; **P < 0.01; ***P < 0.001; ****P < 0.0001. Statistical analysis was performed using 1-way ANOVA with multiple comparisons in **B**, **C**, and **F**, and a Pearson’s correlation test in **D** and **H**.

Lipid peroxidation during colitis promotes the pathogenesis of CAC, making colitis a risk factor for CRC (46–48). Next, we aimed to explore the changes in the NEDD4L gene or protein during CAC. According to The Cancer Genome Atlas and GEO data, the *NEDD4L* gene was significantly downregulated in tumor tissues of patients with CRC and in tumor tissues from CAC mice compared with their normal tissues (Supplemental Figure 13, A and B). The expression of NEDD4L dynamically changed during the AOM/DSS induction. NEDD4L gene and protein showed no significant changes on the 15th day after the AOM/DSS induction but were slightly downregulated on the 60th day when the mice had minor epithelial hyperplasia/dysplasia. Moreover, the gene and protein levels of NEDD4L were significantly downregulated on the 90th day after the AOM/DSS induction, when the mice had obvious neoplasia formation (Figure 10, A–C, and Supplemental Figure 13, C and D). The protein expression of NEDD4L was significantly correlated with both SLC3A2 and GPX4 during the induction of mouse CAC (Figure 10D). NEDD4L expression was significantly inhibited in IECs of adjacent tumor and tumor tissues from CAC mice compared with the distal normal colon (Supplemental Figure 13, E–G). This suggested that the inhibition of NEDD4L expression was a consequence of dysregulated intestinal homeostasis, including inflammation damage and tumor formation. Furthermore, NEDD4L expression was negatively correlated with the survival outcomes, and was significantly reduced in advanced tumor stages (Supplemental Figure 13, H–J). Using tissue microarray–based IHC of colon sections from patients with CRC, we found that protein expression of NEDD4L was significantly inhibited in IECs of colonic tumor tissues compared with normal tissues. Meanwhile, lipid peroxidation was significantly enhanced in IECs from tumor and adjacent-tumor tissues compared with the normal tissues (Figure 10, E and F), consistent with the notion that 4-hydroxynonenal (4-HNE) promotes the development of CRC (46). Moreover, the protein expression of NEDD4L was positively correlated with SLC3A2 and GPX4 in the IECs of patients with CRC (Figure 10, G and H). Consistently, we found that the gene expression of SLC3A2 was significantly correlated with that of GPX4, but not with NEDD4L, in the GEPIA2 database, suggesting a posttranslational modification of NEDD4L on SLC3A2 (Supplemental Figure 13, K and L). Collectively, our data support the notion that the protective role of NEDD4L in the pathogenesis of AOM/DSS–induced CRC in mice was dependent on its controlling SLC3A2/GPX4 axis.

## Discussion

NEDD4L is a conserved HECT E3 ligase highly expressed in human neurons, the lung, and intestinal systems. It is known to regulate the ubiquitination of membrane proteins (21). Herein, we demonstrated that both the gene and protein levels of NEDD4L were significantly downregulated in IECs from patients with IBDs and colorectal cancer. The expression level of NEDD4L was negatively correlated with the disease status of colitis. Additionally, *Nedd4l* deficiency in mice significantly promoted the pathogenesis of colitis and AOM/DSS–induced tumorigenesis.

IEC death is thought to be the main pathological mechanism of dysregulated intestinal homeostasis (13). It has been widely recognized that IEC death induced by apoptosis, necroptosis, and pyroptosis is the first step leading to the destruction of intestinal barrier integrity, thus initiating intestinal mucosa inflammation and resulting in IBDs (1, 3). Therefore, exploring functional proteins involved in maintaining intestinal barrier integrity is of great significance for the early diagnosis and treatment of IBDs. Ferroptosis is a recently defined form of cell death involving lipid peroxidation and iron (Fe). There are some clues that ferroptosis occurs in DSS-induced colitis and IBD and may contribute to their pathogenesis (10, 49, 50). In our study, *Nedd4l* global deficiency in mice exacerbated DSS-induced colitis in comparison with the WT mice. Further bone marrow chimera experiments demonstrated that *Nedd4l* deficiency in non–bone marrow cells aggravated DSS-induced colitis, suggesting an important role of NEDD4L in non–bone marrow cells. Goblet cells are the most abundant cells in the intestine, and NEDD4L is highly expressed in goblet cells but downregulated in IECs of patients with IBDs; thus we employed *Nedd4l* IEC-KO mice to investigate the function of NEDD4L in IECs in colitis. Consistently, *Nedd4l* deficiency in IECs strongly exacerbated DSS/TNBS–induced colitis and AOM/DSS–induced CAC. Further mechanism studies revealed that *Nedd4l* deficiency in IECs induced more severe IEC death and damage of the intestinal barrier by promoting IEC ferroptosis in comparison with WT mice upon DSS treatment, suggesting that the damaged intestinal barrier integrity served as the initiation factor for NEDD4L to modulate DSS-induced colitis. The intestine is a complex organ composed of many cells, including non–bone marrow–derived cells, such as IECs, mesenchymal cells, and endothelial cells, as well as bone marrow–derived cells, including macrophages, monocytes, dendritic cells, lymphocytes, and even innate lymphoid cells, maintaining the intestinal homeostasis through a complex regulatory network. According to scRNA-Seq data, the *NEDD4L* gene was lowly expressed in bone marrow–derived and non–bone marrow–derived cells, indicating a potentially limited regulatory function for NEDD4L in these cells.

NEDD4L expression was reported to be downregulated in many tumors and psoriasis, suggesting a potential biomarker for diseases (29, 51, 52). In our study, we demonstrated that both the NEDD4L gene and protein were downregulated in IECs of patients with colitis or CAC, and this downregulation was correlated with the disease status of colitis and survival outcomes of CRC. Our in vitro cellular data indicated that NEDD4L expression was affected by many pathways ending in cell death and TNF-α. However, given the lack of clinical IBD biopsies from patients with infectious colitis or diverticulitis, we cannot conclude that NEDD4L expression would be inhibited in any inflammatory setting. As colitis develops, intestinal lamina propria infiltrates immune cells, causing secretion of cytokines, particularly TNF-α, a pivotal mediator of inflammation and cell death and also a key therapeutic target in IBD treatment. As predicted based on our in vitro cell line data, TNF-α may impair the expression of NEDD4L in IECs, further amplifying the inflammatory signaling and enhancing cell death in vivo, resulting in aggravated inflammation and epithelial barrier integrity damage, ultimately leading to IBDs. Thus, NEDD4L may act as a general homeostatic regulator of epithelial barrier integrity that could be at a common point in many TNF-α–related pathways that converge to mediate cell injury and death. Accumulating evidence suggests that epigenetic modifications, such as chromatin remodeling or DNA methylation, which occur in response to pathological environmental stimuli, contribute to tissue-specific and disease-associated effects mediated by TNF-α (53). Our previous data have demonstrated that NEDD4L expression could be modulated by the IMQ-induced EZH2/H3K27me3 axis in keratinocytes (29). However, it remains to be determined whether the transcriptional regulation of NEDD4L during intestinal injury or cell death is induced by TNF-α–mediated histone methylation, which could be further explored.

The ubiquitin-proteasome system is a highly finely modulated protein regulation system, which is important for cell proliferation, apoptosis, immunity, and development (54–56), thus regulating inflammatory diseases, tumors, and cardiovascular diseases (54). Based on our unbiased ubiquitination MS sequencing, the ferroptosis signaling pathway was substantially enriched in IECs of DSS-treated *Nedd4l*-deficient mice. Our further biochemistry experiment demonstrated that NEDD4L bound to SLC3A2 and promoted K63-linked ubiquitinoylation while inhibiting K48-linked ubiquitinoylation of SLC3A2, positively regulating the protein stability of SLC3A2, thus inhibiting IEC ferroptosis. Domain mapping data identified that the HECT domain of NEDD4L was required for interaction with and ubiquitinoylation of SLC3A2. Our data suggested that SLC3A2 could be the potential target of NEDD4L in IECs, which seems inconsistent with the reported notion that SLC3A2 (CD98) positively regulates intestinal homeostasis by modulating mouse β_1_ integrin signaling in IECs (36). However, our in vivo and in vitro data demonstrated that SLC3A2 interacted with GPX4, and its protein expression was positively correlated with that of GPX4, but not with cyclin D1, partly consistent with reported data that SLC3A2 is positively correlated with GPX4 (57, 58). Furthermore, ferroptosis-specific inhibitors, Fer-1 and DFOM, or a lipid peroxidation scavenger, NAC, eliminated the phenotypic difference in DSS-induced colitis between *Nedd4l* IEC-deficient mice and WT mice. In contrast, other inhibitors related to signaling of potential targets of NEDD4L, such as BAY 61-3606 and anti–IL-17 neutralizing antibody, could not eliminate the phenotypic difference in DSS-induced colitis. Collectively, our in vitro and in vivo data suggest that NEDD4L modulated SLC3A2 ubiquitinoylation to regulate DSS-induced colitis. Further mechanisms need to be explored to clarify the complicated functions of SLC3A2 in both mouse β_1_ integrin signaling and ferroptosis signaling.

Our study revealed a positive correlation between NEDD4L protein expression and SLC3A2 in humans with IBDs and CRC, demonstrating that the NEDD4L/SLC3A2/GPX4 axis played an important role in colitis and CAC. IL-17R signaling can affect IEC homeostasis, differentiation, and tumor development (38–40). However, our data demonstrated that NEDD4L regulated DSS-induced colitis in an IL-17R signaling–independent manner. Colitis is a risk factor for colon cancer and AOM/DSS model mice have more severe inflammation, which would drive more serious cancer regardless of any cell-intrinsic effect (44, 45), suggesting that blocking IL-17R signaling may have no influence on CAC mediated by *Nedd4l* IEC deficiency. It has been demonstrated that NEDD4 and NEDD4L KO in IECs regulated Lgr5 degradation to mediate Wnt signaling and cancer development in APC^min^ mice (27, 59). In addition, a prior study has implicated NEDD4 in mediating Nrf2 to regulate HO-1– and DSS-induced colitis (60, 61). In epithelial cells, E-cadherin suppresses ferroptosis by activating the intracellular NF2 (also known as Merlin) and Hippo signaling pathway (62). Merlin/NF2, a key activator of the Hippo pathway in growth control and regarded as a key tumor suppressor, is regulated by phosphorylation. However, Merlin ubiquitination is mediated by the E3 ubiquitin ligase NEDD4L, which requires a scaffold protein, AMOTL1, to interact with Merlin (63). Thus, these data suggest a potential role of NEDD4 or NEDD4L in epithelial cell inflammation– and cell proliferation–related colitis or CRC. However, our unbiased ubiquitination MS sequencing data and in vivo experiments support that SLC3A2/GPX4–mediated lipid peroxidation production signaling played a dominant role in controlling colitis and CAC. Whether NEDD4L regulates Lgr5/Wnt signaling or NF2/Yap signaling to control CAC remains to be studied further using their specific inhibitors or genetic KO mice for the CAC model.

The gut microbiota is a key factor in colitis that may directly affect its pathogenesis (2, 64). In our study, cohoused breeding of *Nedd4l*-deficient and WT mice developed comparable severities of DSS-induced colitis, suggesting that gut microbiota plays a pivotal role in NEDD4L-regulated colitis. Further analysis, including 16S rDNA sequencing of feces and in vivo supplementation of commensal intestinal bacteria, revealed that *Lactobacillus* and *Bifidobacterium* were critical for NEDD4L-regulated colitis. Our signaling study demonstrated that supplementation of *Lactobacillus* and *Bifidobacterium* blocked GPX4-mediated ferroptosis signaling, suggesting an important role of these gut microbiota in ferroptosis-mediated colitis.

In conclusion, our study demonstrated a significant reduction in the expression of the E3 ubiquitin ligase NEDD4L in IECs of patients with IBDs and CRC. Additionally, *Nedd4l* KO in mice significantly enhanced DSS/TNBS–induced colitis and AOM/DSS–induced CAC by triggering SLC3A2-mediated ferroptosis. This study provides a potential diagnostic biomarker and clinical treatment target for inflammatory bowel diseases and CAC.

## Methods

Additional methods are provided in Supplemental Methods.

### Sex as a biological variable.

Our study used biopsies from both male and female humans and mice, as sex was not considered as a biological variable.

### Animals.

Heterozygous *Nedd4l* mice (on a BALB/cByJ background) were purchased from The Jackson Laboratory. *NEDD4L^fl/fl^* mice (on a C57BL/6J background) were purchased from Cyagen Bioscience. KO mice and WT littermate control mice were generated by crossing of *Nedd4l*-heterozygous mice. *Nedd4l* IEC-KO mice were generated by crossing of *Nedd4l^fl/fl^* mice with *Villin^Cre^* mice (on a C57BL/6J background). All mice were maintained under the specific pathogen–free condition in the Laboratory Animal Center of Zhejiang University. Eight- to ten-week-old mice were studied using TNBS- or DSS-induced colitis models as described previously (65). For inhibition experiments in vivo, the *Nedd4l^fl/fl^* *Villin^Cre^* and corresponding control mice were treated daily with Fer-1 (5 μmol/kg), DFOM (200 mg/kg), NCA (300 mg/kg), BAY 61-3066 (5 mg/kg), anti–IL-17A antibody (100 μg/mouse), or corresponding control vehicle, respectively, 3 days before 2% DSS administration until to the end of experiments.

### Statistics.

The statistical analysis was performed using a log-rank test for 2-curve survival analysis, a 2-way ANOVA test for 2-curve analysis, a Pearson’s correlation test for correlation analysis, or a 2-tailed unpaired Student’s *t* test for 2-group analysis. When appropriate, the statistical significance of differences among multiple groups was analyzed using 1-way ANOVA with the Bonferroni’s correction. Differences were considered significant at *P* less than 0.05.

### Study approval.

Written patient consent was provided, and ethics approval for human samples was granted by the Medical Ethics Committee of Zhejiang University School of Medicine (ethics approval 2021-005, 20210125-30, IIT20240689B-R1) for harvesting human tissues. All animal research was performed under a protocol approved by the Medical Experimental Animal Care Commission of Zhejiang University (ethics approval 202118445, ZJU20240729).

### Data availability.

Raw data of protein sequencing were deposited in iProX (https://www.iprox.cn/page/home.html) under accession numbers PXD057172 and PXD057173. Raw data of RNA sequencing were deposited in the NCBI’s Gene Expression Omnibus (GEO) database under accession numbers GSE282883 and GSE282497. The values for all data points in the graphs are reported in the Supporting Data Values file.

## Author contributions

J Liang, WL, NW, YY, HW, XA, H Li, H Liu, YJ, and YW performed experiments. J Liang, WL, and YJ performed the statistical analysis. XC and JX provided single-cell analysis. XL, J Lou, and ZX provided some reagents. TZ, XW, and WL designed the study. J Liang and WL drafted the manuscript.

## Supplementary Material

Supplemental data

Unedited blot and gel images

Supporting data values

## Figures and Tables

**Table 2:**
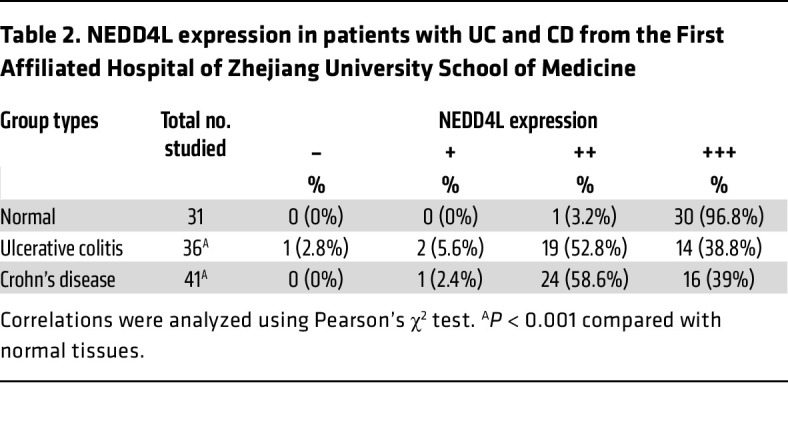
NEDD4L expression in patients with UC and CD from the First Affiliated Hospital of Zhejiang University School of Medicine

**Table 1:**
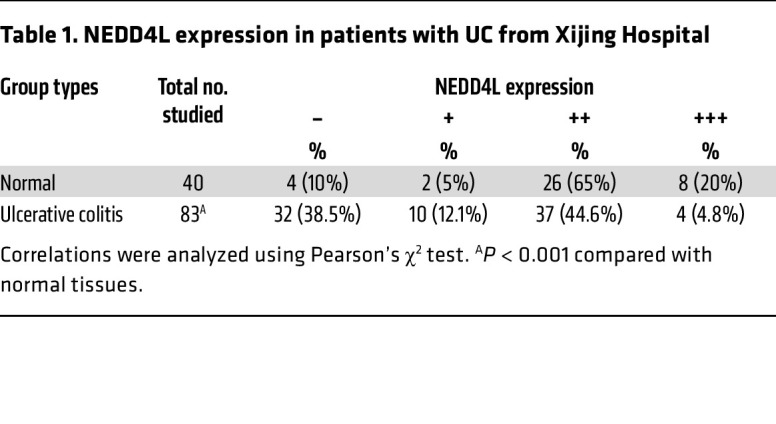
NEDD4L expression in patients with UC from Xijing Hospital

## Version 1 12/17/2024

In-Press Preview

## Version 2 02/03/2025

Electronic publication
