# Supplementary material for: NEDD4L mediates intestinal epithelial cell ferroptosis to restrict inflammatory bowel diseases and colorectal tumorigenesis
Source: J Clin Invest. 2024 Dec 17;135(3):e173994. doi: 10.1172/JCI173994 (PMC11785928; doi:10.1172/JCI173994)

NEDD4L mediates intestinal epithelial cell ferroptosis to restrict inflammatory bowel diseases and colorectal tumorigenesis

Jingjing Liang[1,2#], Ning Wang[1,2#], Yihan Yao[1,2#], Yingmei Wang[3], Xiang An[1,2], Haofei Wang[1,2], Huan Liu[4], Yu Jiang[5], Hui Li[6], Xiaoqing Cheng[7], Jiaqi Xu[7], Xiaojing Liang[7], Jun Lou[8], Zengfeng Xin[1,2], Ting Zhang[9*], Xiaojian Wang[1,2*], Wenlong Lin[1,2*]

[1]The Second Affiliated Hospital, Zhejiang University School of Medicine, Hangzhou, China

[2]School of Medicine, Hangzhou City University, Hangzhou, China

[3]State Key Laboratory of Cancer Biology, Department of Pathology, Xijing Hospital and School of Basic Medicine, the Fourth Military Medical University, Xi'an, China

[4]Institute of Basic and Translational Medicine, Xi'an Medical University, Xi'an, China,

[5]Department of Clinical Laboratory Medicine, Second Affiliated Hospital, School of Medicine, Zhejiang University, Hangzhou, China

[6]Department of Medical Oncology, the Cancer Hospital of the University of Chinese Academy of Sciences (Zhejiang Cancer Hospital), Hangzhou, China

[7]Department of Pathology, Sir Run Run Shaw Hospital, Zhejiang University School of Medicine, Hangzhou, China

[8]Department of Respiratory and Critical Medicine, Sir Run Run Shaw Hospital, Zhejiang University, School of Medicine, Hangzhou, China

[9]Department of Radiation Oncology, The Second Affiliated Hospital, Zhejiang University School of Medicine, Hangzhou, China

#These authors contributed equally.

*Address correspondence to: Wenlong Lin, Zhejiang University, 866 Yuhang Tang Road, Hangzhou, Zhejiang, 310058, P. R. China. Phone: 86.0571.88981662; Email: lwl210@foxmail.com. Or to: Xiaojian Wang, Zhejiang University, 866 Yuhang Tang Road, Hangzhou, Zhejiang, P. R. China; Phone: 86.0571.88201220; Email: wangxiaojian@cad.zju.edu.cn. Or to: Ting Zhang, The Second Affiliated Hospital, Zhejiang University School of Medicine, No. 88 JieFang Road, Hangzhou, Zhejiang,310009， P. R. China; Phone: 86.0571.87783521; Email: zezht@zju.edu.cn.

Supplemental Methods

Reagents

Antibodies for NEDD4L (#4013), human SLC3A2 (#47213), K63-linkage Specific Polyubiquitin (#5621), K48-linkage Specific Polyubiquitin (#8081), Ki-67(#12202), and CyclinD1(#55506) were purchased from Cell Signaling Technology (Cell Signaling Technology, Beverly, MA) for western blotting. Antibody for NEDD4L (HPA024618 for IHC staining of cohort1), TNBS (P2297), M2 (anti-Flag, M8823) beads, AOM (A5486), and FITC-labeled dextran (M.W. =4000, 46944) were purchased from Sigma Aldrich. Antibodies for NEDD4L (ab46521 for IHC staining of cohort2 and ab124643 for mouse IHC staining), 4-HNE (ab108508), TFRC (ab214039), GP130(Ab202850), Lysozyme (Lyz, ab108508), Chromogranin A (ChagA, ab254557), and lipid peroxidation (MDA) assay kit (ab118970) were purchased from Abcam. Antibodies for mouse SLC3A2 (A23839), Ubiquitin (A19686), and GPX4 (A11243) were purchased from ABclonal (ABclonal Technology Co., Ltd). Antibodies for human SLC3A2 (15193-1-AP for immunoprecipitation and IHC staining), ZO-1 (21773-1-AP for IF staining), MEKK2(55106-1-AP), Myc (16286-1-AP), HA (51064-2-AP), and Actin (66009-1-Ig) were purchased from Proteintech (Proteintech Group, Inc). BODIPY™ 581/591 C11(D3861) was purchased from Thermo Fisher Scientific Inc. Ferrostatin-1 (Fer-1, HY-100579), deferoxamine mesylate (DFOM, HY-B0988), and acetylcysteine (NAC, HY-B0215) were purchased from MedChemExpress. DSS was purchased from MP Biomedicals (M.W. =36,000-50,000, 160110). Commercial capsules containing *Lactobacillus* and *Bifidobacterium* (1045220) were purchased from Swisse Wellness PTY LTD. The anti-IL-17A antibody (cat. BE0173, 100 μg) and anti-

isotype (cat.BE0083) antibody were purchased from BioxCell. PEI was purchased from Polyscience (24765), and INTERFERin@ (101000028) was purchased from Polyplus Transfection.

Human samples

Human paraffin-embedded colon sections from patients with IBDs or normal control colon sections were obtained from the Department of Pathology, Xijing Hospital and School of Basic Medicine, the Fourth Military Medical University (also called Xijing Hospital, cohort1) and the Department of Pathology, Frist Affiliated Hospital, Zhejiang University (cohort2, cohort3). cDNA and protein samples of patients with IBDs and their corresponding normal individuals were kind gifts of D.r Weidong Han (Sir Run Run Shaw Hospital, Zhejiang University School of Medicine, Hangzhou, China). Normal control colon sections consisted of healthy tissue from the resection edges of tumor biopsies that appeared healthy at the histological level. The basic information for all the patients, including age, sex, and colitis location, is summarized in the supplemental Table 1-3.

Plasmids

cDNA encoding NEDD4L or SLC3A2 was amplified by PCR using the cDNA of the human HCT116 cell as a template and cloned in the pCMV-Entry vector or pcDNA3.1-Flag vector, respectively. The vectors for NEDD4L mutants were subsequently generated by PCR amplification. Myc-tagged NEDD4L WT or its variants, including NEDD4L-ΔC2, NEDD4L-ΔWW, NEDD4L-ΔHECT, and NEDD4L-C942A, were generated by PCR and subcloned into pcDNA3.1-EGFP-Myc-His. HA-Ub-K6R, K11R, K27R, K29R, K33R, K48R, and K63R or HA-Ub-K6-only(O), K11O, K27O, K29O, K33O, K48O, and K63O plasmids were

generated by point mutation method using HA-Ub plasmid.

The severity of UC or CD disease determinations

The severity of UC disease was determined by the physician according to Truelove and Witt's protocol including defecation (times/d), bleeding, pulse (times/min), temperature (°C), hemoglobin, ESR (mm/1 h), etc. The severity of CD disease was determined according to the Best CDAI calculator.

IHC staining and score

Human colonic specimens from patients with IBDs, colorectal cancer, and normal control colon sections were immunohistochemically stained with anti-NEDD4L, GPX4, SLC3A2, or 4-HNE antibodies and scored using Constantine's protocol. Briefly, integrated staining intensity and positive cell percentage were semi-quantitatively scored under high magnification. Staining intensity was scored as follows: 0 = no color; 1 = yellow; 2 = brown-yellow; and 3 = brown. The proportion of positive cells was graded as follows: 0=positive cells <10%; 1 = positive cells between 10% and 40%; 2 = positive cells between 40% and 70%; and 3 = positive cells≥70%. The staining intensity score and proportion of positive cells score were added up: 0 = negative staining, marked-; 0-2 = weak expression, marked +; 2-4 = moderate expression, marked++; and 4-6 = strong expression, marked+++. All the IHC staining was scored by professional pathologists in a double-blinded manner.

Single-cell RNA-Seq analysis.

Single-cell data used in this study were acquired from the Single Cell Portal (SCP259)(1). A total of 366,650 cells from the colon mucosa of 18 UC patients and 12 healthy individuals were downloaded as raw data for epithelial and immune cells. Data processing, including

batch correction, doublet removal, gene annotation, and cell clustering, was performed as previously described. After that, the Seurat R package (version 2.3.2) was used to normalize expression values for total unique molecular identifier counts per cell. The statistical significance was assessed using Kruskal-Wallis test.

Determination of Bleeding scores

Briefly, on day 0, the normal phenotype was registered as the baseline clinical score. Mice were scored blindly during the colitis experiment. Bleeding scores were determined as follows: 0 = no blood as examined by Hemoccult (Beckman Coulter) analysis; 1 = positive hemoccult; 2 = visible blood traces in stool; 3 = visible blood traces that adhered to the anus; and 4 = gross bleeding.

Histological score

For histological analysis, paraffin-embedded sections (4 mm thick) were subjected to H&E staining. Histological scores were determined blindly based on the previously described(2) criteria with some modifications (5): 0 = normal; 1 = moderate mucosal inflammation without erosion or ulcer; 2 = severe mucosal inflammation with erosion; 3 = severe mucosal inflammation with ulcer (<1 mm); and 4 = severe mucosal inflammation with ulcer (>1 mm).

Bone Marrow Chimeras

Bone marrow chimeras were performed as reported before(2). Briefly, recipient mice underwent a sub-lethal dose of γ-ray irradiation (8.5Gly) to kill the bone marrow cell and six hours post-irradiation, WT and KO recipients received 100 ul fresh WT bone marrow cells with the concentration of 1×10^{8}/ml, respectively, which are WT→WT and WT→KO groups. 8 weeks after bone marrow transplantation, the mice’s blood was collected and

determined with a NEDD4L genotyping analysis to exclude failure mice, then fed with 3% DSS for the indicated time to induce colitis.

Isolation of intestinal lamina propria cells

The murine intestinal lamina propria cells (IELs) were isolated as previously described with little modification(2). Isolated IELs were stained with anti-mouse immune cell markers, CD45(Biolegend, 103126, 103116), CD3(Biolegend, 100204), CD4 (Biolegend, 100414), CD8(Biolegend, 100708), CD19(Biolegend, 152410), F4/80(Biolegend, 123115), CD11b (Biolegend, 101208), CD11c (Biolegend, 117318), Gr-1(Biolegend, 108406), and Fixable Viability (423114, 423102) for flow cytometer analysis using the Novocytes FACS system.

Isolation of IECs

Dissected mouse colons were cut open longitudinally and incubated in 50-ml tubes with 20 ml Solution 1 (Ca^{2+}- and Mg^{2+}-free Hank's Buffered Salt Solution (CMF-HBSS; Invitrogen) containing 10 mM dithiothreitol and 1.5 mM EDTA) at 4°C for 30 mins and then 20 ml Solution 2 (Ca^{2+}- and Mg^{2+}-free Hank's Buffered Salt Solution containing 10 mM 1.5 mM EDTA) at 4°C for another 30 mins. After incubation, the tube was vigorously shaken by hand (at speed =10g) for 30 seconds to dislodge IECs. The cell suspensions were passed through a 100-μm cell strainer (BD Biosciences) and were centrifuged at 4°C at 200 g for 5 minutes. The purity and viability for IEC were approximately 90% analyzed by flow cytometer using a staining solution containing antibodies to mouse CD45(Biolegend, 157214), EpCAM (Biolegend, 118214), and propidium Iodide (PI, Biolegend ,421301). The cell pellet was resuspended and lysed for MS, western blotting, qPCR, MDA, or flow cytometer analysis.

Crypt isolation and organoid culture

The intestinal organoids were derived from the small intestines as reported(3), with slight modifications. In brief, 10 cm small intestines were dissected and opened longitudinally to remove luminal contents. The intestine was cut into 5 mm pieces and incubated with 4 mM EDTA in PBS for 30 minutes at 4°C without shaking. Crypts were dissociated from villi by pipetting and filtered through a 70 μm strainer (BD Biosciences), followed by centrifugation (4°C, 200g, for 5 minutes) and washing. The purified crypts were resuspended in Matrigel (Corning, 356231), seeded onto a glass-bottom dish, and then cultured in IntestiCult Organoid Growth Medium (StemCell Technologies, 06005). Organoid growth medium was refreshed every 1–2 days. For the BODIPY C11 plus DAPI-traced organoid cell ferroptosis assay, DMSO(Control), DSS (0.5% w/v), Erastin (30μM, Selleck Chemicals, S7242), Erastin2 (30μM, Selleck Chemicals, E1874), or RSL3 (5μM, Selleck Chemicals, S8155) were added into organoid growth medium for 24 hours on day 3. Then organoids were stained with 0.1 μg/mL DAPI and 5μM BODIPY C11 in HBSS at 37°C for 30min, and washed with PBS twice. The images were captured using a confocal microscope (FV1000, Olympus). After that, the organoids were digested and resuspended in a cell staining buffer with 2% FBS for the flow cytometer analysis.

FITC-dextran permeability assay

Intestinal permeability was assessed through the oral administration of FITC-labeled dextran as previously described(2).

Immunohistochemical staining for Lyz, ChagA, ALP, Ki67, and TUNEL assay

Immunohistochemical staining for Lyz, ChagA, ALP, Ki67, and the TUNEL assay (one-step

TUNEL apoptosis assay kit, Roche, 12156792910) was performed for mouse colonic sections by the Histomorphology Platform, Zhejiang University, with the standard protocol performed according to the manufacturer's instructions. Immunohistochemical and TUNEL slides were examined with an Olympus microscope. Lyz, ChagA, Ki67, or TUNEL-positive cells in the entire section were counted under a microscope (n=3-4/group).

Cell culture, plasmid transfection, and siRNA silencing

HCT116 (CCL-247), SW480 (CCL-228), RKO (CRL-2577), and HEK293T (CRL-11268) cells were obtained from American Type Culture Collection (ATCC), and grown in Dulbecco's modified Eagle's medium (DMEM) supplemented with 10% fetal bovine serum (FBS, ExCELL, FSP500). The HCT116 and HEK293T cells were transfected with PEI according to the manufacturer's protocol. Scramble siRNA and NEDD4L targeted siRNA were transfected in HCT116, SW480, and RKO cells using INTERFERin@ according to the manufacturer's protocol. The following siRNA oligonucleotide sequences were used: NEDD4L siRNA (5′-GAGUCCUAUCGGAGAAUUATT-3′), SLC3A2 siRNA (5′-CCGAGAAGAAUGGUCUGGUGAAGAU-3′), Scramble siRNA (Negative control) (5′-UUCUCCGAACGUGUCACGUTT-3′).

NEDD4L KO HCT116 cell line generation

The cell was generated as in our reported paper(4). Small guide RNAs (gRNA) target NEDD4L for knockout were designed and subcloned into a PEP-KO (PEP-330X) vector. After transfection, HCT116 cells were further screened using 1μg/ml puromycin. The surviving cell was further transferred into the 96-well plate to form monoclonal cell lines. The KO cells were detected by real-time PCR and WB analysis. The sequences for

NEDD4L gRNAs were listed in Supplemental Table 4.

For cell stimulation

The cells were treated as reported(3, 5), with slight modifications. In brief, The HCT116, SW480, and RKO cells were plated on 12-well plates at 5 × 10^4 cells per well overnight and then stimulated with DMSO(Control), DSS (2% w/v), Erastin(30μM), Erastin2 (30μM), or RSL3 (5μM) for 36hr. For multiply cell death, the HCT116 cells were stimulated with TNF T/S/Z mix-TNF-α (50 ng/mL, Abclonal, RP00993); SM-164 (50 nM, Selleck Chemicals, S7089); Z-VAD-FMK (50 μM ,Selleck Chemicals, S7023) to induced cell necroptosis, TNF-α (50 ng/M) plus CHX (50 μg/ ml, Sigma Aldrich, 239764) to induce cell pryotosis, staurosporine (2nM) to induce cell apoptosis, IL-17(100ng/ml, Abclonal, RP02414B), IL-1α (50ng/ml, Abclonal, RP00098), or DSS (2%) as positive control for the indicated time. Cell lysates and culture supernatant were further analyzed by western blotting.

Measurement of cell death, cell viability, lipid peroxidation and malondialdehyde

To induce ferroptosis, the HCT116, SW480, and RKO cells were seeded in 12-well plates at 2 × 10^4 cells per well overnight and then pretreated with or without Fer-1 for 1 hr. Cell viability was determined using the CCK8 or MTT assay according to the manufacturer's instructions. Viability was calculated by normalizing treated OD levels to untreated OD with a normal medium. To analyze lipid peroxidation, cells were stained 5 μM BODIPY-C11 for 30 min at 37°C in HBSS followed by flow cytometric analysis. Lipid ROS-positive cells are defined as cells with FITC fluorescence greater than 99% of the unstained sample. Malondialdehyde (MDA) contents of the colonic epithelial cells (IECs) were detected using a lipid peroxidation assay kit (Abcam) following standard instructions. The absorbance of

the resulting mixture was measured at 535 nm with a spectrophotometer.

Immunoprecipitation and western-blot analysis

SDS-PAGE and western blots were performed as described previously(2). Co-transfected HEK293T cell lysates were immunoprecipitated using anti-Flag or anti-Myc antibodies plus protein A/G agarose. The proteins were then separated using SDS-PAGE and subjected to western blot analysis with indicated antibodies.

Ubiquitination assay

Ubiquitination assays were performed as described previously(2, 4), with slight modifications. For endogenous SLC3A2 ubiquitination assay, sg*NTC* or sg*NEDD4L* HCT116 cells were lysed and boiled for 10 min in lysis buffer supplemented with 1% SDS. After 5 min cooling in ice, the cell lysate was diluted ten times with lysis buffer, and then centrifugation for 10 min under 12,000 g at 4°C, the supernatants were collected and subjected to overnight incubation with anti-SLC3A2 antibody and protein A/G magnetic beads (Bio-Rad). After incubation, the beads were washed three times with TBS (50 mM Tris-HCl, 150 mM NaCl, pH 7.4) and then eluted by loading buffer and subsequently, for SDS–PAGE separation. For exogenous SLC3A2 ubiquitination assay, HEK293T cells were transfected with Flag-tagged SLC3A2, Myc-tagged NEDD4L, and HA-tagged Ubiquitin or its mutants, 36 hr later, 20 μM (Final concentration) of MG-132 were pre0treated for 6hr, and then the cells were harvested and processed as described in endogenous ubiquitination assay. The Protein A/G magnetic beats were substituted by ANTI-FLAG M2 beads. In vitro ubiquitination assay: In brief, 150 ng E1, 300 ng E2 (UbcH5α), 500 μg/ml Ubiquitin, 1 μg Flag-tagged SLC3A2, 1 μg Myc-tagged NEDD4L, or

1 µg Myc-tagged NEDD4L-C942A/Myc-tagged EGFP were reacted in ubiquitination buffer which contains 25 mM Tris–HCl, PH 7.6, 5 mM MgCl2, 100 mM NaCl, 0.2 µM DTT, and 2 mM ATP for 2 hr under 30°C. Loading buffer was added to stop the reaction and subsequently subjected to SDS-PAGE separation and WB detection. Proteins E1, E2 (UbcH5α), and ubiquitin were kindly provided by Professor ZongPing Xia (Zhengzhou University, Henan, China) for in vitro ubiquitination assay.

AOM/DSS model of colorectal tumorigenesis

Male and female mice were used at the age of 6-10 weeks, and then were injected intraperitoneally with AOM (10 mg/kg, A5486, Sigma). Three days later, 2 % DSS was given in the drinking water for 5 days followed by regular drinking water for 2 weeks. This cycle was repeated twice, and mice were sacrificed on day 90. According to the diameter of the tumors in mice colon on day 90 of AOM/DSS model, we divided them into three group: small tumors, <1 mm; medium tumors, 1 mm ⩽ and ⩽ 2 mm; large tumors, >2 mm. Tumor load was calculated according to the following formula: tumor load = (number of small tumors) × 1 + (number of medium tumors) × 2 + (number of large tumors) × 3.

Colonic MRI

Colonic MRI was performed as reported before(6). All the mice were placed in the supine position at the center of the mouse coil. The mice were anesthetized by intraperitoneal injection of 4% chloral hydrate (400 mg/kg). A cleansing enema with water was administered 20 min after the liquid enema (Gd-FITC-SLNs), and imaging session was subsequently undertaken after distending the colorectum by 1 mL of room air through a 1-mL syringe and a 24-gauge cannula (Xindeyi Medical Instrument Co. Ltd., Hangzhou,

China). Leakage from the rectum was prevented through a small rubber seal placed into the anus of each mouse.

RNA-sequencing, ubiquitylation mass spectrometry, and 16S rDNA sequencing

The colonic tissues, IECs, and feces collected on the 7th day from the *Nedd4l*$^{f/f}$*Villin*^{Cre} and *Nedd4l*$^{f/f}$ mice administered with 2% DSS were subjected to RNA-sequencing and ubiquitylation mass spectrometry analysis by Novogene Co., Ltd or to 16S rDNA sequencing analysis by Magigene Co., Ltd, respectively.

Realtime Quantitative PCR

Total RNA was isolated using TRIzol and cDNA was synthesized with a reverse-transcription kit (TAKARA, Ostushiga, JAPAN). The expression of genes was detected by a LightCycler 480 system with SYBR Premix Ex Tap. The data was calculated by a standard curve method and normalized to the expression of the gene encoding 18s RNA. The specific primers for individual genes are in Supplemental Table 5.

References

1. Smillie CS, Biton M, Ordovas-Montanes J, Sullivan KM, Burgin G, Graham DB, et al. Intra- and Inter-cellular Rewiring of the Human Colon during Ulcerative Colitis. *Cell.* 2019;178(3):714-30 e22.
2. Lin W, Ma C, Su F, Jiang Y, Lai R, Zhang T, et al. Raf kinase inhibitor protein mediates intestinal epithelial cell apoptosis and promotes IBDs in humans and mice. *Gut.* 2017;66(4):597-610.
3. Xu J, Li S, Jin W, Zhou H, Zhong T, Cheng X, et al. Epithelial Gab1 calibrates RIPK3-dependent necroptosis to prevent intestinal inflammation. *JCI Insight.* 2023;8(6).
4. Liu H, Lin W, Liu Z, Song Y, Cheng H, An H, et al. E3 ubiquitin ligase NEDD4L negatively regulates keratinocyte hyperplasia by promoting GP130 degradation. *EMBO Rep.* 2021;22(5):e52063.
5. Wu J, Minikes AM, Gao M, Bian H, Li Y, Stockwell BR, et al. Intercellular interaction dictates cancer cell ferroptosis via NF2-YAP signalling. *Nature.* 2019;572(7769):402-6.
6. Tian M, Wang X, Sun J, Lin W, Chen L, Liu S, et al. IRF3 prevents colorectal tumorigenesis via inhibiting the nuclear translocation of beta-catenin. *Nat Commun.* 2020;11(1):5762.

Supplemental Figure Legends

Supplemental Figure 1. Expression of NEDD4L in the public database.

(A) Single-cell sequencing data of *NEDD4L* gene expression in multiple of cells from The Human Protein Atlas. **(B)** *NEDD4L* gene expression in human colonic cells according to the published single-cell RNA-seq database (SCP259). **(C-D)** Dot-plots of *NEDD4L* gene expression levels in colon biopsies from healthy controls (HC), and patients with CD, or UC (using Gene Expression Omnibus (GEO) accession number GSE75214; normal colon (HC) n=11, activate-CD n=8, activate-UC n=74, inactivate-UC n=23, normal ileum n=11, activate-CD-ileum n=51, inactivate-CD-ileum n=16) **(C)**; or in UC-uninflamed mucosa and UC-inflamed mucosa (using dataset GSE11223; HC n=72, UC-uninflamed n=66, UC-inflamed n=66) **(D)**. **(E)** Dot-plots of *NEDD4L* gene expression levels in colonic mucosa from healthy controls (non-inflamed) or patients with UC-inflamed, or UC-uninflamed (using dataset GSE9452; HC n=5, UC-inflamed n=8, UC-uninflamed n=13). **(F)** Dot-plots of *NEDD4L* gene expression levels in PBMCs from healthy controls (HC), patients with CD or UC (using dataset GSE3365; HC n=42, CD n=56, UC n=26). **(G)** Dot-plots of *NEDD4L* gene levels in colonic mucosa from patients with UC-non-involved, UC-active-involved, and UC-remission-involved (using dataset GSE38713; HC n=13, UC-non-involved n=7, UC-active-involved n=15, and UC-remission-involved n=8). **(H)** Dot-plots of *NEDD4L* gene expression levels in colonic mucosa from twins with ulcerative colitis (diseased individual) or healthy control of the twins (healthy individual) (using dataset GSE22619; twins pair n=10). **(I)** Box plots of single-cell of colonic crypts from 18 UC patients with UC and 12 healthy individuals by analyzing a single-cell sequencing database (Single Cell Portal (SCP259)). Box plots show the interquartile range (box), median (line), and minimum and maximum (whiskers). **(J)** Representative NEDD4L IHC staining of colon sections from mice treated with DSS for 0,2, 4 days. **(K)**qPCR analysis of *Nedd4l* gene expression in IECs from **(J)**. Scale bar, 50 μm.

Data represent mean ± SEM. Each dot means independent samples. ns, no significant difference. ****, P<0.0001; ***, P<0.001; **, P<0.01. Statistical analysis was performed using a 1-way ANOVA multiple comparisons test in **C-G**, and **K**, a 2-tailed Student’s t-test

in **H**, and a Kruskal-Wallis test in **I**.

Supplemental Figure 2. *Nedd4l* knockout in mice enhances sensitivity to experimental colitis and deficiency in intestinal epithelial cells (IECs) is dispensable for the differentiation of IECs under steady state.

(A-E) *Nedd4l* global-knockout mice (*Nedd4l*$^{-/-}$), global-deficient mice (*Nedd4l*$^{+/-}$), and control littermates (*Nedd4l*$^{+/+}$) were administered with 1 % DSS for 7 days followed by water to induce colitis. Mouse death was monitored until day 9. **(A)**Body weight change (*Nedd4l*$^{+/+}$ n=10, *Nedd4l*$^{+/-}$ n=6, *Nedd4*$^{-/-}$ n=6), **(B)** colon length, **(C)** gross morphology images, **(D)** histological score, and **(E)** representative H&E staining of the colon sections from mice on day 9. Scale bar, 100 μm. **(F, G)** H&E, PAS, ALP, immunohistochemical staining of the lysosome (Lyz), and Chromogranin A (ChgA) of small intestine **(G)** or colon **(G)** sections as indicated. The images in the black boxes in the upper left corner were enlarged positive staining targets. Scale bar,100 μm. **(H, I)** Statistical analysis of the Lyz and ChgA IHC staining positive cells per crypt of the small intestine **(H)** or colon **(I)** sections as indicated. **(J, K)** qPCR analysis of the gene expressions in the small intestine **(J)** or colon **(K)** of *Nedd4l*$^{f/f}$*Villin*^{Cre} and *Nedd4l*$^{f/f}$ mice as indicated. n=4/group.

Data represent mean ± SEM. Each dot means independent samples. ns, no significant difference. **, P<0.01; *, P<0.05. Statistical analysis was performed using a log-rank test in **A**, and a 2-tailed Student's t-test in **H**, **I**, **J**, and **K.**

Supplemental Figure 3. *Nedd4l* deficiency in IECs promotes TNBS-induced colitis in mice.

Nedd4l IEC-deficient mice (*Nedd4l*$^{f/f}$*Villin*^{Cre}) and control littermates (*Nedd4l*$^{f/f}$) were challenged intrarectally with 50%TNBS (150 mg/kg) dissolved in ethanol and then monitored until day 5. **(A)** Death rate (*Nedd4l*$^{f/f}$ n=14, *Nedd4l*$^{f/f}$*Villin*^{Cre} n=10), **(B)** body weight change (*Nedd4l*$^{f/f}$ n=11, *Nedd4l*$^{f/f}$*Villin*^{Cre} n=13), **(C)** colon length, **(D)** gross morphology images, and **(E)** H&E staining of colon sections from *Nedd4l*$^{f/f}$*Villin*^{Cre} and *Nedd4l*$^{f/f}$ mice were measured on day 5. **(F)** Colonic lamina propria cells staining of **(B)** with anti-mouse immune cell markers were analyzed by flow cytometer (n=3–4/group). Red arrows point to epithelial degeneration and green arrows to inflammatory infiltrates. Scale

bar, 200 μm or 50 μm (amplified sections).

Data represent mean ± SEM from at least two independent experiments. Each dot means independent samples. ns, no significant difference. **, P<0.01; *, P<0.05. Statistical analysis was performed using a log-rank test in **A,** a two-way ANOVA test in **B**, and a 2-tailed Student's t-test in **C** and **F.**

Supplemental Figure 4. *Nedd4l* deficiency in IECs promotes IEC ferroptosis resulting in barrier integrity damage.

(A, B) *Nedd4l*$^{f/f}$*Villin*^{Cre} and *Nedd4l*$^{f/f}$ mice were administered without or with 2 % DSS for 5 days to induce colitis, and on the 7th day, the mice were sacrificed for collecting IECs which were subjected to ubiquitylation mass spectrometry analysis. **(A)** GO analysis of untreated- or DSS-treated IECs, and **(B)** KEGG analysis of untreated IECs. **(C, D)** *Nedd4l*$^{f/f}$*Villin*^{Cre} and *Nedd4l*$^{f/f}$ mice were administered 2 % DSS for 5 days to induce colitis. On the 9th day, mice were sacrificed for collecting IECs subjected to qPCR analysis for ferroptosis-related genes (*Gpx4* and *Tfrc*) and inflammation-related genes (*Ptgs2* and *Lcn2*). **(E, F)** Western blotting analysis of HCT116 cells treated with DSS (2%), Erastin (30μM), or RSL3(5μM) for the indicated time **(E)**, and **(F)** protein intensity analysis of NEDD4L according to **(E)** was shown. **(G, H)** Western blotting analysis of HCT116 cells treated with T/S/Z mix-TNF-α (50 ng/M), SM-164 (50 nM), and Z-VAD-FMK (50 μM) to induced cell necroptosis, TNF-α (50 ng/M) plus CHX(50 μg/ ml) to induce cell pyroptosis, staurosporine (2nM) to induce cell apoptosis, and DSS (2%) as positive control for the indicated time **(G)**, and protein intensity analysis of NEDD4L **(H)** according to **(G)**. n=3/group. **(I, J)** Western blotting analysis of HCT116 cells treated with TNF-α (20ng/ml), IL-17(100ng/ml), or IL-1α (50ng/ml) for the indicated time **(I)**, and protein intensity analysis of NEDD4L **(J)** according to **(I)**.

Data represent mean ± SEM from at least two independent experiments. Each dot means independent samples. ns, no significant difference. ***, P<0.001; **, P<0.01; *, P<0.05. Statistical analysis was performed using a 2-tailed Student's t-test in **C** and **D**, and 1-way ANOVA multiple comparisons in **F**, **H**, and **J**.

Supplemental Figure 5. NEDD4L negatively regulates ferroptosis.

(A, B) HCT116 cells were knocked out using the CRSIP Cas9 system targeted to NEDD4L (sg*NEDD4L*) or negative control (sg*NTC*). The cells were treated with 2% DSS for the indicated time and then subjected to CCK8 assay to measure cell viability **(A)** and flow cytometer analysis after being stained with BODIOPY C11 in HBSS to measure lipid peroxidation production**(B)**. **(C-E)** HCT116 cells were overexpressed by transfecting with Myc-tagged NEDD4L, its E3 ligase activity mutant Myc-tagged NEDD4L-C942A(Myc-NEDD4L-CA), or Myc-tagged null control plasmids (Myc-null). The cells were treated with 2% DSS for the indicated time and then subjected to CCK8 assay to measure cell viability **(C)**, flow cytometer analysis after being stained with BODIOPY C11 in HBSS to measure lipid peroxidation production**(D),** and MDA assay **(E)**. **(F-K)** The multitype cell lines, including HCT116 cells **(A)**, SW480 cells **(B)**, and RKO cells **(C)** were knockdown using the siRNA silencing system targeted to *NEDD4L* (si*NEDD4L*) or negative control (si*NC*). The cells were treated with 2% DSS for the indicated time and then subjected to CCK8 assay to measure cell viability **(F, G, H)** and flow cytometer analysis after being stained with BODIOPY C11 in HBSS to measure lipid peroxidation production **(I, J, K)**. **(L-S)** HCT116 cells were knockout using the CRSIP Cas9 system targeted to NEDD4L (sg*NEDD4L*) or negative control (sg*NTC*) **(L, P)** or knockdown using the siRNA silencing system targeted to *NEDD4L* (si*NEDD4L*) or negative control (si*NC*) **(M-O, Q-S)**.The cells were treated with DSS (2%), Erastin (30μM), or RSL3(5μM) for the indicated time and then subjected to CCK8 assay to measure cell viability **(L-O)** and flow cytometer analysis after being stained with BODIOPY C11 in HBSS to measure lipid peroxidation production**(P-S)**.n=3/group.

Data represent mean ± SEM from at least two independent experiments. Each dot means independent samples. ns, no significant difference. ***, P<0.001; **, P<0.01; *, P<0.05. Statistical analysis was performed using a 2-tailed Student's t-test.

Supplemental Figure 6. SLC3A2 is a potential target in the NEDD4L-mediated mice colitis model.

(A) *Nedd4l*$^{f/f}$*Villin*^{Cre} and *Nedd4l*$^{f/f}$ mice were sacrificed for collecting IECs and then subjected to ubiquitylation mass spectrometry analysis. Volcano plots of protein

abundance fold change analysis based on the data from ubiquitylation mass spectrometry were shown. **(B)** Flag-tagged NEDD4L and Flag-tagged control plasmids overexpressed HCT116 cells were immunoprecipitated with anti-Flag antibody for further interaction mass spectrometry (MS) analysis. The list showed the overlapped targets of NEDD4L in **(A)** and interaction MS without treatment, and the $\log_2$ FC and P values indicate the enrichment values of the *Nedd4l*$^{f/f}$*Villin*^{Cre} group normalized to the *Nedd4l*$^{f/f}$ group. **(C)** The list showed the score and rank of the overlapped targets of NEDD4L in MS data. **(D)** *Nedd4l*$^{f/f}$*Villin*^{Cre} and *Nedd4l*$^{f/f}$ mice were sacrificed for collecting IECs which were subjected to western blotting analysis of GPX4, SLC3A2, MEKK2, GP130, NEDD4L, and actin. **(E)** Correlation analysis of protein abundance of NEDD4L with SLC3A2 based on the two ubiquitylation mass spectrometry analysis data. **(F)** Statistical analysis of *Slc3a2* and *Cyclind1* mRNA from DSS-treated *Nedd4l*$^{f/f}$*Villin*^{Cre} and *Nedd4l*$^{f/f}$ mice. **(G-I)** *Nedd4l*$^{f/f}$ mice were administered 2 % DSS for 5 days to induce colitis. On day 0, day 2, day 4, and day 6, the mice were sacrificed for collecting IECs respectively, and then subjected to western blotting analysis**(G)**, **(H)** statistical analysis, and **(I)** correlation analysis of indicated proteins, including NEDD4L, SLC3A2, and GPX4 protein intensity from the samples of **(G).** n=3/group. **(J, K)** HCT116 cells were knocked out using the CRSIP Cas9 system targeted to NEDD4L (sg*NEDD4L*) or negative control (sg*NTC*). The cells were treated with Erastin (30μM), or RSL3(5μM) for the indicated time and then subjected to immunoblot analysis of GPX4, SLC3A2, TFRC, NEDD4L, and actin. **(L, M)** The HCT116 cells were knocked down using a siRNA silencing system targeted to NEDD4L (si*NEDD4L*) or negative control (si*NC*). The cells were treated Erastin (30μM), or RSL3(5μM) for the indicated time and then subjected to immunoblot analysis of GPX4, SLC3A2, TFRC, NEDD4L, and actin.

Data represent mean ± SEM from at least two independent experiments. Each dot means independent samples. ns, no significant difference. ****, $P<0.0001$; ***, $P<0.001$; **, $P<0.01$; *, $P<0.05$. Statistical analysis was performed using a 2-tailed Student's t-test in **F** and **H,** and a Pearson correlation test in **E** and **I.**

Supplemental Figure 7. SLC3A2 negatively regulates ferroptosis.

(A-F) The multitype cell lines, including HCT116 cells **(A)**, SW480 cells **(B)**, and RKO cells

(C) were knocked down using a siRNA silencing system targeted to *SLC3A2* (si*SLC3A2*) or negative control (si*NC*). The cells were stimulated with DMSO(CTRL), Erastin (30μM), Erastin2 (30μM), or RSL3(5μM) for the indicated time, and then subjected to CCK8 assay to measure cell viability**(A-C)** and flow cytometer analysis after being stained with BODIOPY C11 in HBSS to measure lipid peroxidation production**(D-F)**. n=3/group. **(G-H)** The HCT116 cells were knocked down using a siRNA silencing system targeted to SLC3A2 (si*SLC3A2*) or negative control (si*NC*). The cells were treated with Erastin (30μM), Erastin 2 (30μM), or RSL3(5μM) for the indicated time and then subjected to immunoblot analysis of GPX4, SLC3A2, TFRC, and actin.

Data represent mean ± SEM from at least two independent experiments. Each dot means independent samples. ns, no significant difference. ****, P<0.0001; ***, P<0.001; **, P<0.01; *, P<0.05. Statistical analysis was performed using a 2-tailed Student's t-test in **A**-**F**.

Supplemental Figure 8. NEDD4L ubiquitinates SLC3A2.

(A) Immunoblot analysis of Flag-tagged SLC3A2 co-immunoprecipitated with anti-Myc antibody from lysates of HEK293T cells co-transfected with a siRNA-specific to *NEDD4L* (si*NEDD4L*) or scramble siRNA (si*NC*). **(B)** Immunoblot analysis of SLC3A2, NEDD4L, and actin in HCT116 cells transfected Flag-tagged null (NT), Flag-tagged NEDD4L, and Flag-tagged NEDD4L C942A treated with DMSO, 20μM MG-132, or 0.2 μM bafilomycin A1 (Baf A1) for 6 hr. **(C)** Immunoblot analysis of SLC3A2, NEDD4L, and actin in HCT116 cells transfected with plasmids expressing Flag-tagged null (CTRL), Flag-tagged NEDD4L, and Flag-tagged NEDD4L C942A followed by being treated with CHX (50 μg/ ml) for the indicated time. **(D)**Immunoblot analysis of the interaction of Flag-tagged NEDD4L, SLC3A2 with Myc-tagged GPX4 which was co-immunoprecipitated by Flag-tagged antibody from lysates of HEK293T cells pre-treated with 20μM MG-132 for 6 hr. **(E)**Immunoblot analysis of total ubiquitination of Myc-tagged GPX4 followed by being immunoprecipitated Myc-tagged GPX4 with anti-Myc specific antibody from lysates of HEK293T cells co-transfected with plasmids expressing HA-tagged Ub, and Flag-tagged wild-type NEDD4L or NEDD4L-C942A(CA). Results represent at least two independent experiments.

Supplemental Figure 9. NEDD4L regulates DSS-induced colitis in an IL-17R

signaling- or a Syk signaling-independent manner.

Nedd4l$^{f/f}$*Villin*^{Cre} and *Nedd4l*$^{f/f}$ mice were administered 2 % DSS for 5 days to induce colitis. On the 7th day mice were sacrificed for collecting colonic tissues which were subjected to an RNA-sequencing analysis. **(A)** KEGG analysis and **(B)** GO analysis were shown. **(C-J)** *Nedd4l*$^{f/f}$*Villin*^{Cre} and *Nedd4l*$^{f/f}$ mice were administered 2 % DSS for 5 days to induce colitis, during the induction of colitis mice were intraperitoneally injected with anti-isotype antibody or anti-IL-17A antibody on day 0, day 2, day 4, and day 6 **(C-F)** (*Nedd4l*$^{f/f}$ +isotype n=5, *Nedd4l*$^{f/f}$*Villin*^{Cre}+isotype n=5, *Nedd4l*$^{f/f}$ +anti-IL-17A n=6, *Nedd4l*$^{f/f}$*Villin*^{Cre}+ anti-IL-17A n=4), or a Syk specific inhibitor BAY 61-3066**(G-J)** (*Nedd4l*$^{f/f}$ +BAY 61-3066 n=3, *Nedd4l*$^{f/f}$*Villin*^{Cre}+ BAY 61-3066 n=4), respectively. **(G, K)** Body weight change, **(H, L)** colon length, **(I, M)** gross morphology images, and **(F, J)** H&E staining of the colons from *Nedd4l*$^{f/f}$*Villin*^{Cre} and *Nedd4l*$^{f/f}$ mice on day 8 or 9 were measured. Scale bar, 100 μm or 50 μm (amplified sections). **(K-N)** *Nedd4l*$^{f/f}$*Villin*^{Cre} and *Nedd4l*$^{f/f}$ mice were administered 2 % DSS for 5 days to induce colitis, during the induction of colitis mice were orally administrated with NAC or water daily (*Nedd4l*$^{f/f}$*Villin*^{Cre}+ddH_2O n=4, *Nedd4l*$^{f/f}$ +NAC n=5, *Nedd4l*$^{f/f}$*Villin*^{Cre}+ NAC n=3). **(K)**Body weight change, **(L)** colon length, **(M)** gross morphology images, and **(N)** H&E staining of the colons from *Nedd4l*$^{f/f}$*Villin*^{Cre} and *Nedd4l*$^{f/f}$ mice on day 8 were shown. Scale bar, 100 μm.

Data represent mean ± SEM from at least two independent experiments. Each dot means independent samples. ns, no significant difference. ****, P<0.0001; ***, P<0.001; **, P<0.01; *, P<0.05. Statistical analysis was performed using a two-way ANOVA test in **C**, **G**, and **K,** 1-way ANOVA multiple comparisons in **D** and **L**, and a 2-tailed Student's t-test in **H**.

Supplemental Figure 10. NEDD4L regulates DSS-induced colitis through ferroptosis.

(A-B) *Nedd4l*$^{f/f}$*Villin*^{Cre} and *Nedd4l*$^{f/f}$ mice were treated according to **Figure 8A**, and on the 7th day mice were sacrificed for isolating IECs for qPCR analysis of tight junction genes (*Tjp1, Cldn1, Cldn2*, and *Ocln*) and collecting colonic tissues for qPCR analysis of inflammation-related genes (*Il6, Il1β, Mcp1*, and *Mip2*). *Nedd4l*$^{f/f}$+DMSO n=3, *Nedd4l*$^{f/f}$*Villin*^{Cre}+DMSO n=3, *Nedd4l*$^{f/f}$ +Fer-1 n=5-6, *Nedd4l*$^{f/f}$*Villin*^{Cre}+Fer-1 n=5, as indicated in the Figure. **(C-K)** *Nedd4l*$^{f/f}$*Villin*^{Cre} and *Nedd4l*$^{f/f}$ mice were administered 2 %

DSS pre-treated with ferroptosis inhibitor deferoxamine mesylate (DFOM,200mg/kg) or ddH_2O to induce colitis, and on the 7th day mice were sacrificed for collecting colonic tissues and the IECs. *Nedd4l*$^{f/f}$+ ddH_2O n=3-7, *Nedd4l*$^{f/f}$*Villin*^{Cre}+ ddH_2O n=3-5, *Nedd4l*$^{f/f}$ +DFOM n=4-6, *Nedd4l*$^{f/f}$*Villin*^{Cre}+DFOM n=4-6, as indicated in the Figure. **(C)** Body weight change, **(D)** colon length, and **(E)** gross morphology images of colons from the mice. **(F-I)** In a separate experiment, the IECs and colon tissues from mice treated as in **(C)** were subjected to flow cytometer analysis after being stained with anti-EpCAM, anti-CD45, and PI in HBSS **(F, G),** 4-HNE IHC staining **(H),** ZO-1 IF staining **(I),** western blotting analysis**(J)**, and protein intensity analysis of GPX4, TFRC, and SLC3A2 according to **(J)**.

Data represent mean ± SEM from at least two independent experiments. Each dot means independent samples. ns, no significant difference. ****, $P<0.0001$; ***, $P<0.001$; **, $P<0.01$; *, $P<0.05$. Statistical analysis was performed using 1-way ANOVA multiple comparisons in **A**, **B**, **D**, **F**, **G**, and **K**, and a two-way ANOVA test in **C**.

Supplemental Figure 11. The gut microbiota is involved in NEDD4L-regulated colitis. **(A-F)** *Nedd4l*$^{+/-}$mice or *Nedd4l*$^{f/f}$*Villin*^{Cre} and control littermates (*Nedd4l*$^{+/-}$ *or Nedd4l*$^{f/f}$) were co-housed or single-housed inculcation for 2 weeks followed by administration of 3% or 2.5 % DSS for 5 days to induce acute colitis. Mice were monitored until day 8. **(A, D)** Body weight change, **(B, E)** colon length, and **(C, F)** gross morphology images of the colons from separately housed (single-housed) or co-housed mice were measured on day 8. *Nedd4l*$^{+/+}$ single-housed n=5, *Nedd4l*$^{+/-}$ single-housed n=7, *Nedd4l*$^{+/+}$ co-housed n=6, *Nedd4l*$^{+/-}$ single-housed n=6; *Nedd4l*$^{f/f}$ +single-housed n=9, *Nedd4l*$^{f/f}$*Villin*^{Cre}+ single-housed n=7, *Nedd4l*$^{f/f}$ +co-housed n=6, *Nedd4l*$^{f/f}$*Villin*^{Cre}+ single-housed n=6. **(G)** Heatmap based on 16S rDNA sequencing of feces from *Nedd4l*$^{f/f}$*Villin*^{Cre} or *Nedd4l*$^{f/f}$ mice with or without DSS treatment on day 5. **(H-J)** AB-PAS staining of the colon sections and qPCR analysis of antimicrobial peptide-related genes (*Lysozyme, Ang4, Defa-rs1*, and *Defa20*) in small intestines **(I, J)** from *Nedd4l*$^{f/f}$*Villin*^{Cre} or *Nedd4l*$^{f/f}$ mice with or without DSS treatment on day 7. *Nedd4l*$^{f/f}$ n=4, *Nedd4l*$^{f/f}$*Villin*^{Cre} n=4, *Nedd4l*$^{f/f}$ +DSS n=7, *Nedd4l*$^{f/f}$*Villin*^{Cre}+DSS n=6. Scale bar, 50 μm. **(K-N)** *Nedd4l*$^{f/f}$*Villin*^{Cre} mice and control *Nedd4l*$^{f/f}$ mice were gavage the mixture of *Bifidobacterium* and *Lactobacillus (Bif & Lac)* for 1 week followed by being

administrated 2% DSS for 5 days to induce colitis. **(K)** Body weight change, **(L)** colon length, **(M)** gross morphology images, **(N)** H&E, and PAS staining of the colon sections from *Nedd4l*$^{f/f}$*Villin*^{Cre} and *Nedd4l*$^{f/f}$ mice treated with DSS were measured on day 7. *Nedd4l*$^{f/f}$ n=3, *Nedd4l*$^{f/f}$*Villin*^{Cre} n=4, *Nedd4l*$^{f/f}$ +*Bif&Lac* n=5, *Nedd4l*$^{f/f}$*Villin*^{Cre}+ *Bif&Lac* n=3. **(O-P)** In a separate experiment, the IECs from mice treated as in **(K)** were subjected to western blotting analysis **(O)**, and protein intensity analysis of GPX4, TFRC, and SLC3A2 **(P)** according to **(O)**. Scale bar, 100 μm.

Data represent mean ± SEM from at least two independent experiments. Each dot means independent samples. ns, no significant difference. ****, P<0.0001; ***, P<0.001; **, P<0.01; *, P<0.05. Statistical analysis was performed using a two-way ANOVA test in **A, D,** and **K**, 1-way ANOVA multiple comparisons in **B**, **E**, **L**, and **P**, and a 2-tailed Student's t-test in **I** and **J**.

Supplemental Figure 12. *Nedd4l* deficiency in mice promotes AOM/DSS-induced colorectal cancer.

(A-E) The *Nedd4l*$^{+/-}$ (n=23) and *Nedd4l*$^{+/+}$ (n=24) mice were subjected to AOM/DSS treatment to set up a mouse colorectal cancer model. **(A)** Representative morphology image of tumor in colons, **(B)** tumor incidence**, (C)** tumor size analyzed from the AOM/DSS-treated mice on day 90. **(D, E)** Statistical analysis of Ki67 positive cells **(D)** and representative immunohistochemical staining of sections from the tumor, adjacent tumor, and distal normal tissue of AOM/DSS treated *Nedd4l*$^{+/-}$ and *Nedd4l*$^{+/+}$ mice with anti-Ki67antibody**(E)**. n=5. Scale bars, 50 μm.

Data represent mean ± SEM from at least two independent experiments. Each dot means independent samples. ns, no significant difference. **, P<0.01; *, P<0.05. Statistical analysis was performed using a 2-tailed Student's t-test in **C** and **D**.

Supplemental Figure 13. Expression of NEDD4L is significantly down-regulated in IECs of patients and mice with colorectal cancer.

(A) *NEDD4L* gene expression in multi-types of cancer in the TIMER2.0 database was shown. **(B)** The gene expression value of *NEDD4L* in colonic tissues from AOM/DSS-treated mice of the GEO dataset was analyzed. n=3/group. **(C-D)** Representative H&E **(C)**

and anti-NEDD4L immunohistochemical staining **(D)** of colon sections from wild-type mice in **Figure 10A**. Scale bars, 100 μm. **(E-G)** Representative IHC staining of sections from the tumor, adjacent tumor, and distal normal tissues of AOM/DSS treated wild-type mice with anti-NEDD4L antibody, **(F)** statistical analysis of NEDD4L IHC staining intensity (n=5) according to **(E)**, and **(G)** mRNA expression of *NEDD4L* in distal normal colon tissues and tumor tissues (n=10) from AOM/DSS-treated mice on day 90. Scale bars, 50 μm. **(H)** Kaplan–Meier curves of overall survival in the set of patients with rectal cancer (READ) based on *NEDD4L* gene expression level detected in tumor tissues from The Human Protein Atlas database. The median value of *NEDD4L* gene expression in the TCGA data-set was 3.94 (FPKM). The expression value of the $NEDD4L^{high}$ group (n=53) was 3.94-15.8 (FPKM) and the $NEDD4L^{low}$ group (n=86) was 0-3.93 (FPKM). **(I-K)** *NEDD4L* gene expression based on the TCGA database **(I)**, and correlative analysis between SLC3A2, GPX4, and NEDD4L gene expression levels detected in the set of patients with READ based on GIPEA2 database **(J, K)**.

Data represent mean ± SEM from at least two independent experiments. Each dot means independent samples. ns, no significant difference. ***, P<0.001; **, P<0.01; *, P<0.05. Statistical analysis was performed using 1-way ANOVA multiple comparisons in **B**, **F**, and **I**, a log-rank test in **H**, a 2-tailed Student's t-test in **G**, and a Pearson correlation test in **J** and **K**.

Supplemental Table 1. Basic information of normal control and UC patients from Xijing Hospital

	Normal control<br>n=40	UC<br>n=83
**Age(years)**		
**<=30**	**0**	**18**
**30-50**	**13**	**31**
**=>50**	**27**	**34**
**Gender**		
**Male**	**18**	**45**
**Female**	**22**	**38**
**Location**		
**Colon**	**40**	**83**

Supplemental Table 1. Basic information of normal control and UC patients from Xijing Hospital. Human samples were obtained from Xijing Hospital, including 40 non-IBD normal control human colon sections from the resection edges of tumor biopsies that appeared healthy at the histological level and 83 human UC colon sections from screening colonoscopies. The diagnosis of UC was based on a standard combination of clinical, endoscopic, histological, and radiological criteria. The severity of macroscopic inflammation of the colon mucosa at colonoscopy was graded by a professional pathologist.

Supplemental Table 2. Basic information of normal control, UC, and CD patients from FAZHU

	Normal control	UC	CD
	n=31	n=36	n=41
Age(years)			
<=30	0	9	13
30-50	9	11	11
=>50	22	16	17
Gender			
Male	20	17	22
Female	12	19	19
Location			
Colon	31	32	13
Terminal ileum	0	0	12
Rectum	0	4	4
Ileocecal	0	0	12

Supplemental Table 2. Basic information of non-IBD normal control, UC, and CD patients from FAZHU. Human samples were obtained from FAZHU, including 31 non-IBD normal control human colon sections from the resection edges of tumor biopsies that appeared to be healthy at the histological level, and 36 human UC colon sections and 41 human CD colon sections from screening colonoscopies. The diagnosis of CD or UC was based on a standard combination of clinical, endoscopic, histological, and radiological

criteria. The severity of macroscopic inflammation of the colon mucosa at colonoscopy was graded by a professional pathologist.

Supplemental Table 3. Basic information of CD patients from FAZHU

	CD n=17
**Age(years)**	
**<=30**	**5**
**30-50**	**6**
**=>50**	**6**
**Gender**	
**Male**	**6**
**Female**	**11**
**Location**	
**Colon**	**5**
**Terminal ileum**	**4**
**Rectum**	**0**
**Ileocecal**	**8**

Supplemental Table 3. Basic information of CD patients from FAZHU. 17 human CD colon sections were obtained from FAZHU. The diagnosis of CD was based on a standard combination of clinical, endoscopic, histological, and radiological criteria. The severity of macroscopic inflammation of the colon mucosa at colonoscopy was graded by a professional pathologist.

Supplemental Table 4. List of gRNAs for CRISPR-cas9

Sequences of sgRNA for NEDD4L	Sequence (5'-3')
1# Forward	ACCGATCAGTTCCGTGGACTGTC
1# Reverse	AACGACAGTCCACGGAACTGATC
2# Forward	ACCGGGATTTTTGCGATTGAAAA
2# Reverse	AACTTTTCAATCGCAAAAATCCC
NEDD4L identification primer Forward	GTGTGGATAGTGACATCTAGTGG
NEDD4L identification primer Reversed	CTCCACGTACCTCCATGTCAT

Supplemental Table 5. List of primers for real-time PCR

mSlc3a2-F	GAGGACAGGCTTTTGATTGC
mSlc3a2-R	ATTCAGTACGCTCCCCAGTG
mPtgs2-F	TGAGCAACTATTCCAAACCAGC
mPtgs2-R	CACGTAGTCTTCGATCACTATC
mGpx4-F	GCCTGGATAAGTACAGGGGTT
mGpx4-R	CATGCAGATCGACTAGCTGAG
mNcoa4-F	GAACCATCAGGACACATGGAAA
mNcoa4-R	AGGAGCCATAGCCTTGGGT
mAcsf2-F	CTTCGGGAGGCTGTGTATCG
mAcsf2-R	CACCATTCCAGAACTGAGAGC
mAcsl4-F	CTCACCATTATATTGCTGCCTGT
mAcsl4-R	TCTCTTTGCCATAGCGTTTTTCT
18s-F	AAGTCCCTGCCCTTTGTACACA
18s-R	GCCTCACTAAACCATCCAATCG
mLcn2-F	ATGTCACCTCCATCCTGGTC
mLcn2-R	CACACTCACCACCCATTCAG
mCyclin D1-F	CAGACGTTCAGAACCAGATTC
mCyclin D1-R	CCCTCCAATAGCAGCGAAAAC
mNedd4l-F	CACGGGTGGTGAGGAATCC
mNedd4l-R	GCCGAGTCCAAGTTGTGGT
mDefa-F	CACCACCCAAGCTCCAAATACACAG
mDefa-R	ATCGTGAGGACCAAAAGCAAAT
mLyz1-F	GAGACCGAAGCACCGACTATG
mLyz1-R	CGGTTTTGACATTGTGTTCGC
mReg4-F	GGCGTGCGGCTACTCTTAC
mReg4-R	GAAGTACCCATAGCAGTGGGA
mChgA-F	CCAAGGTGATGAAGTGCGTC
mChgA-R	GGTGTCGCAGGATAGAGAGGA
mAnpep-F	ACGCTCAGGAGAAGAATAGGAA
mAnpep-R	CTTAGGCAAGCGATACTGGTTC
mFabp2-F	GTGGAAAGTAGACCGGAACGA
mFabp2-R	CCATCCTGTGTGATTGTCAGTT

mOlfm4-F	CAGCCACTTTCCAATTTCACTG
mOlfm4-R	GCTGGACATACTCCTTCACCTTA
mAscl2-F	AAGCACACCTTGACTGGTACG
mAscl2-R	AAGTGGACGTTTGCACCTTCA
mlysozyme 1-F	GTCACTGCCCAGGCCAAGGT
mlysozyme 1-R	CGGTGCTTCGGTCTCCACGG
mDefa-rs1-F	TGCCCTCGTTCTGCTGGCCT
mDefa-rs1-R	AGCAGAGCCTTCTGTGCCTCCA
mDefa20-F	TGGCCTTCCAGGTCCAGGCT
mDefa20-R	CCTGGTCCTCCTCCCCTGGC
mAng4-F	GCCAAATGGCCGGGACGACA
mAng4-R	GGCCTGGGAGACGCTCCTGA
mTRFC1-F	GTTTCTGCCAGCCCCTTATTAT
mTRFC1-R	GCAAGGAAAGGATATGCAGCA
hNEDD4-F:	GACATGGAGCATGGATGGGAA
hNEDD4-R	GTTCGGCCTAAATTGTCCACT
mMIP-2-F	CACTCTCAAGGGCGGTCAAA
mMIP-2-R	TACGATCCAGGCTTCCCGGGT
mIL-1β-F	TCGCTCAGGGTCACAAGAAA
mIL-1β-R	CATCAGAGGCAAGGAGGAAAAC
mIL-6F	ACAAGTCGGAGGCTTAATTACACAT
mIL-6R	TTGCCATTGCACAACTCTTTT C
mMCP-1-F	ACTGAAGCCAGCTCTCTCTTCCTC
mMCP-1-R	TTCCTTCTTGGGGTCAGCACAGAC
mTjp1-F	GCCGCTAAGAGCACAGCAA
mTjp1-R	GCCCTCCTTTTAACACATCAGA
mCldn1-F	TGCCCCAGTGGAAGATTTACT
mCldn1-R	CTTTGCGAAACGCAGGACAT
mCldn2-F	AGTACCCTTTTAGGACTTCCTGC
mCldn2-R	CCCACCACAGAGATAATACAAGC
mOcln-F	CTGGATCTATGTACGGCTCACA
mOcln-R	TCCACGTAGAGACCAGTACCT

Supplemental Figure 1

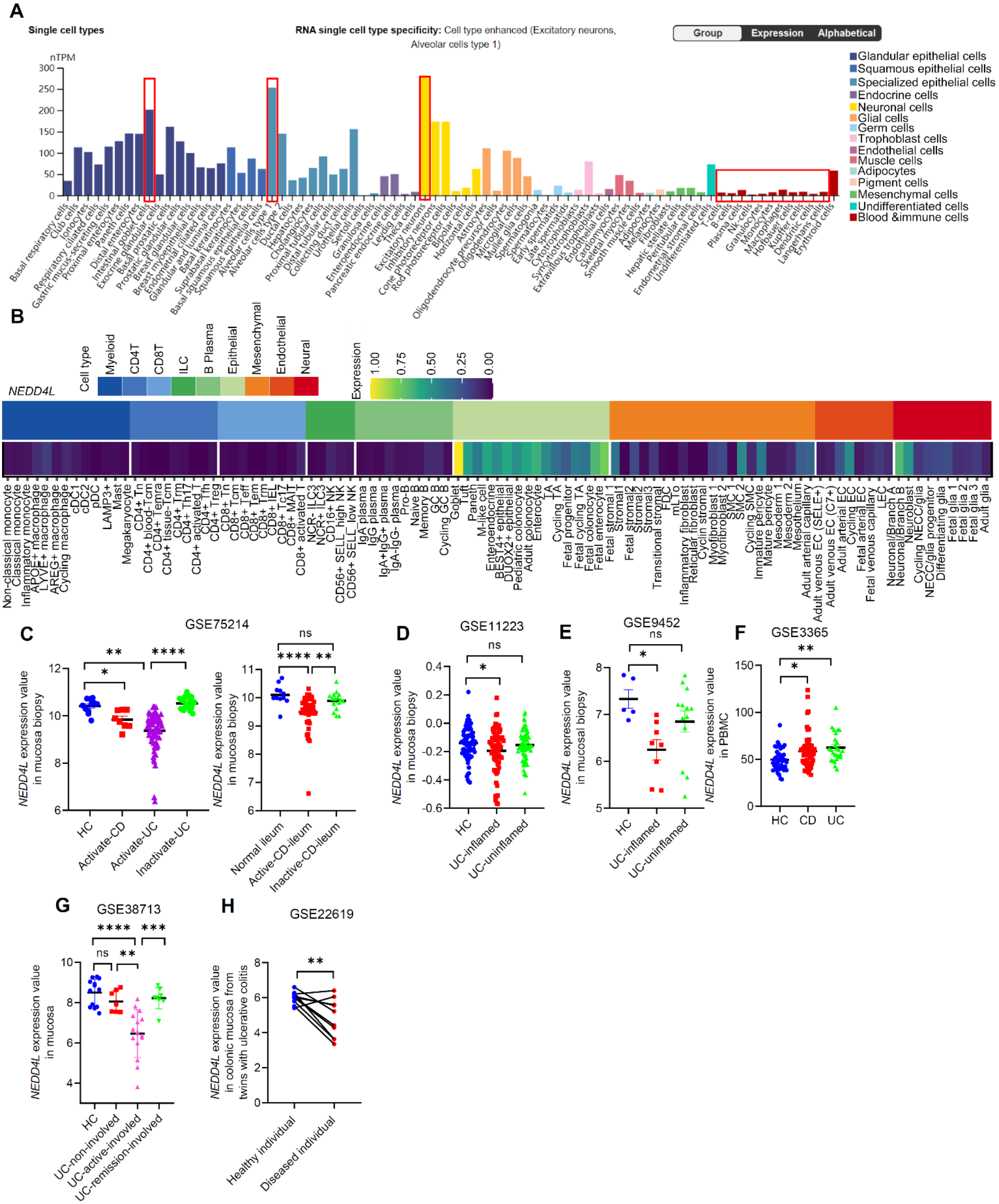

Supplemental Figure 1

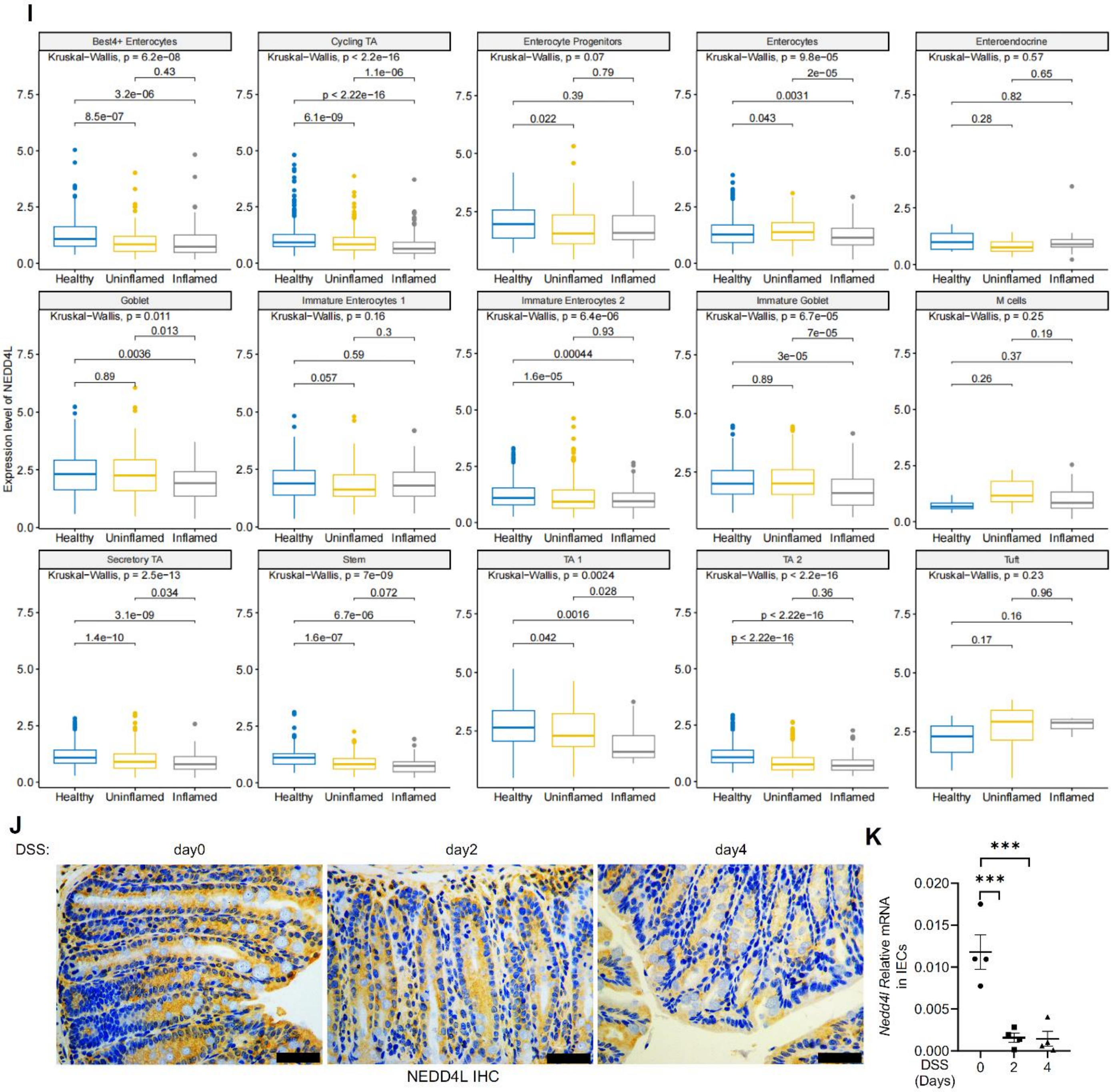

Supplemental Figure 2

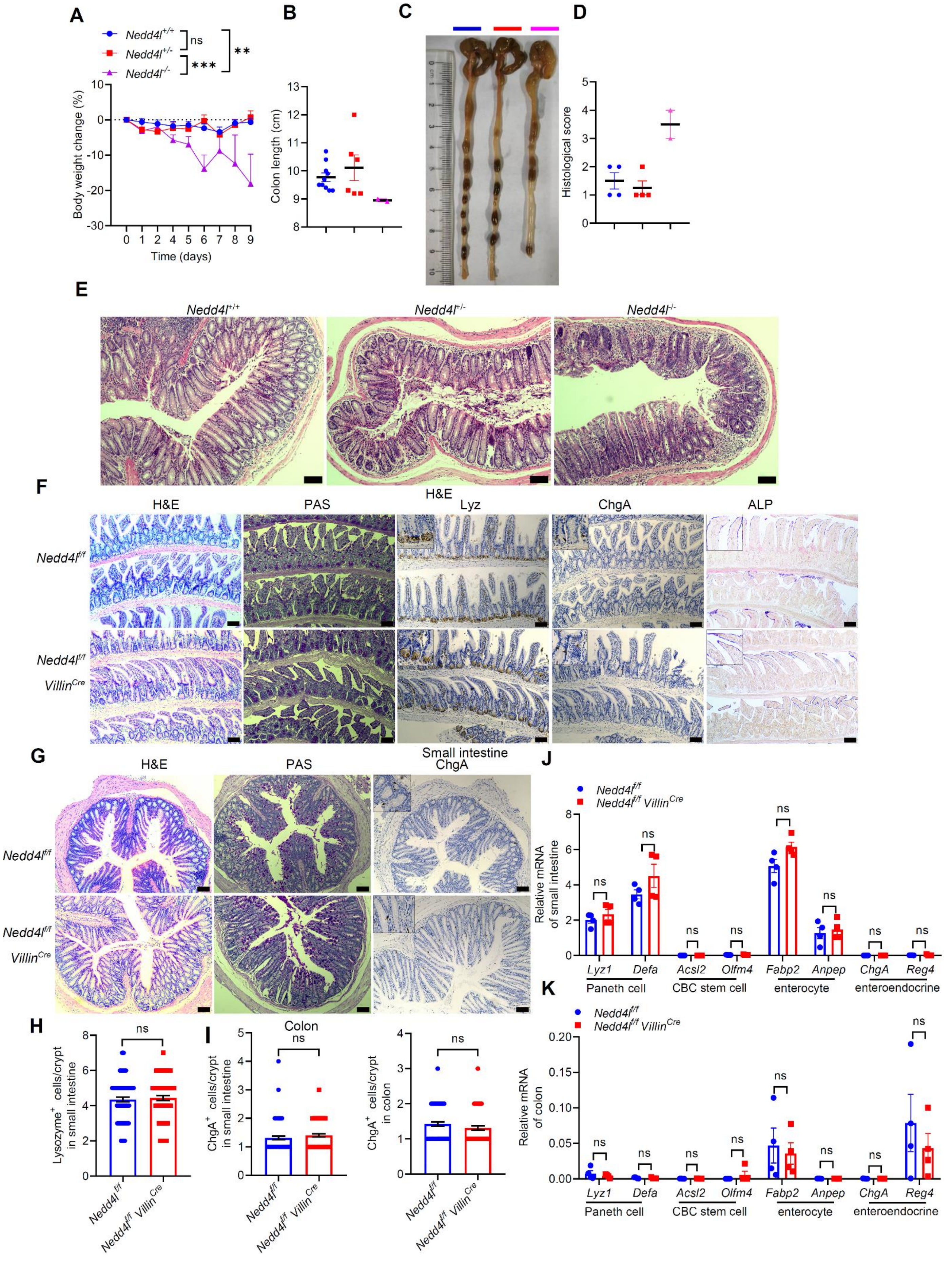

Supplemental Figure 3

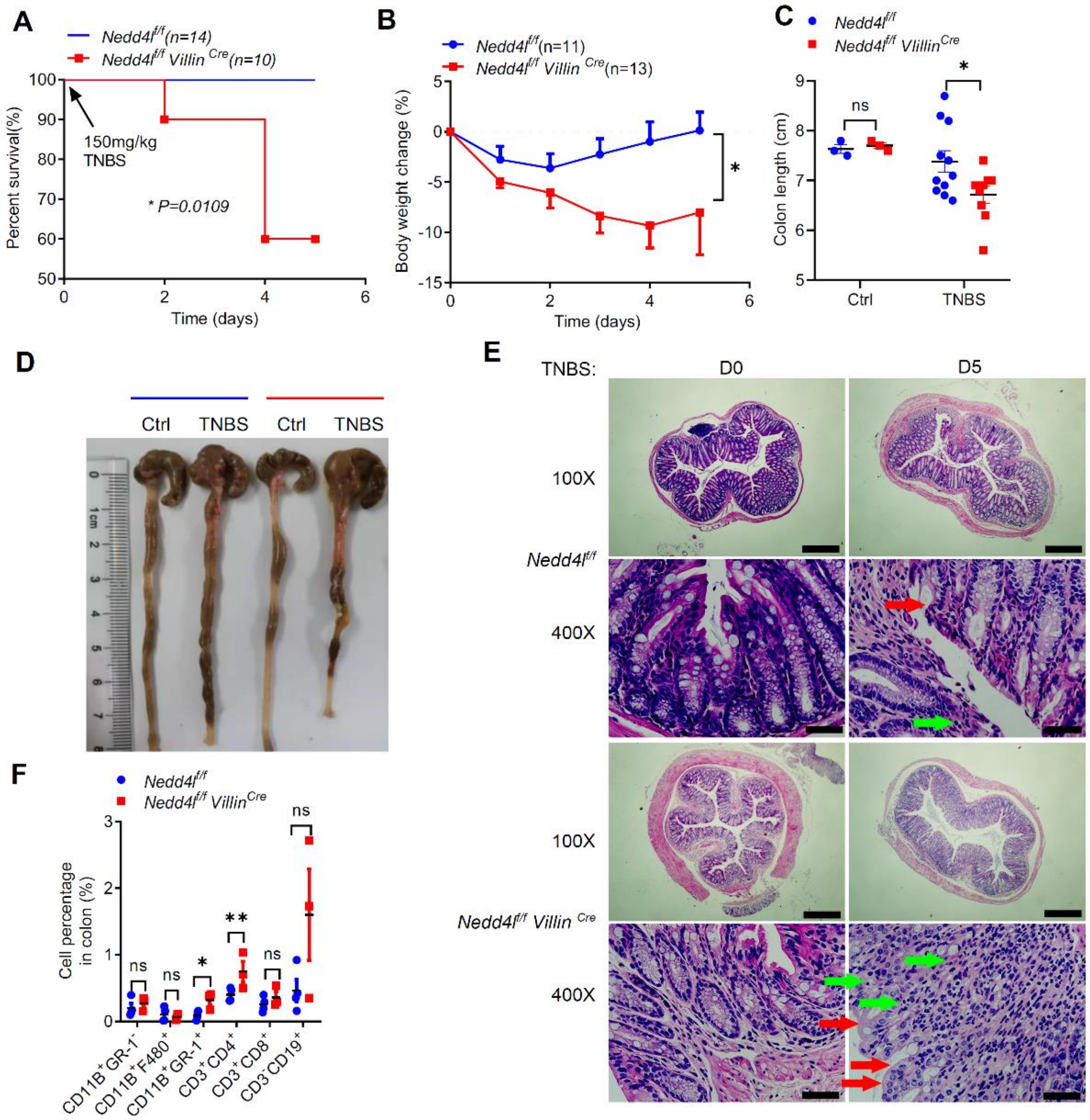

Supplemental Figure 4

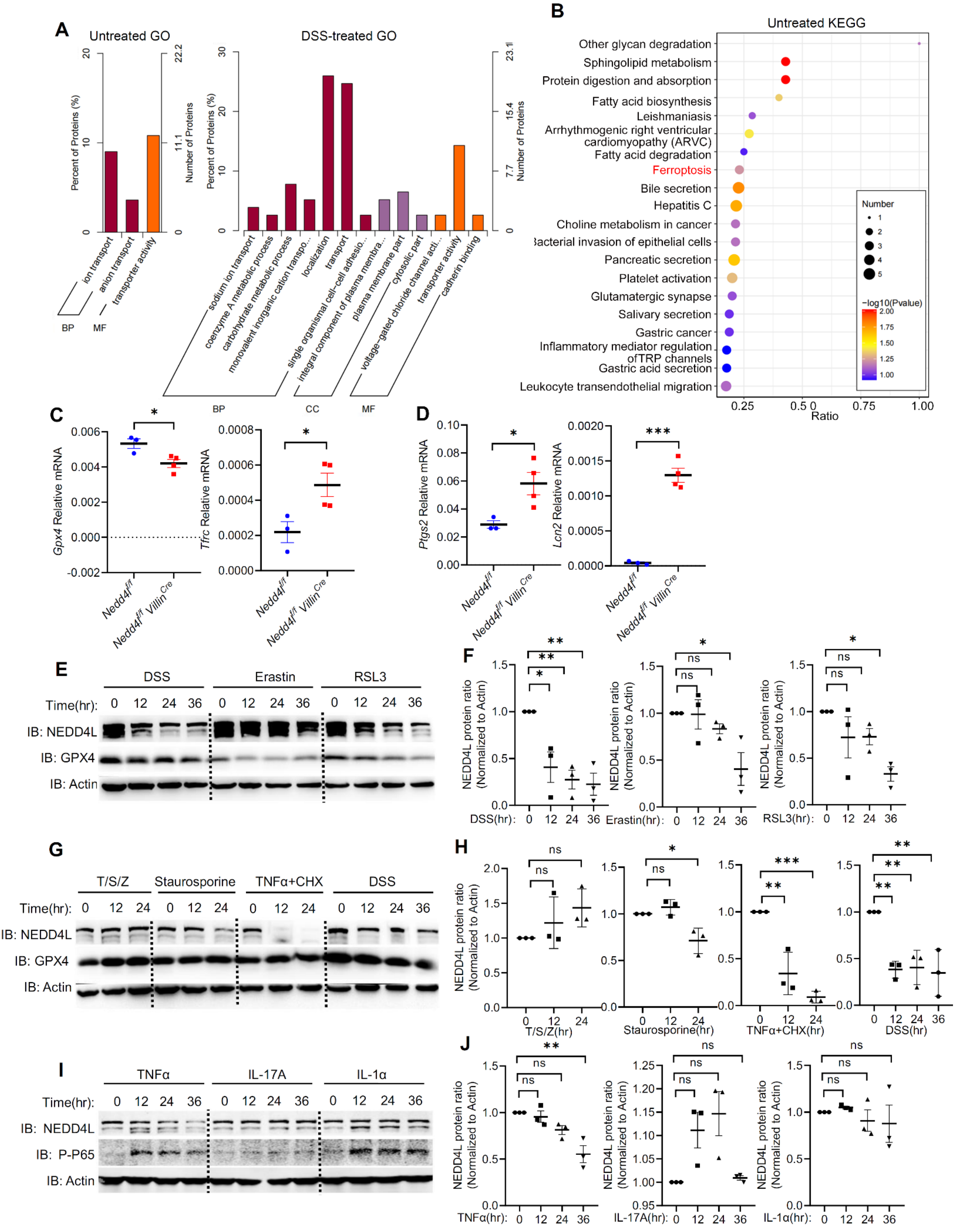

Supplemental Figure 5

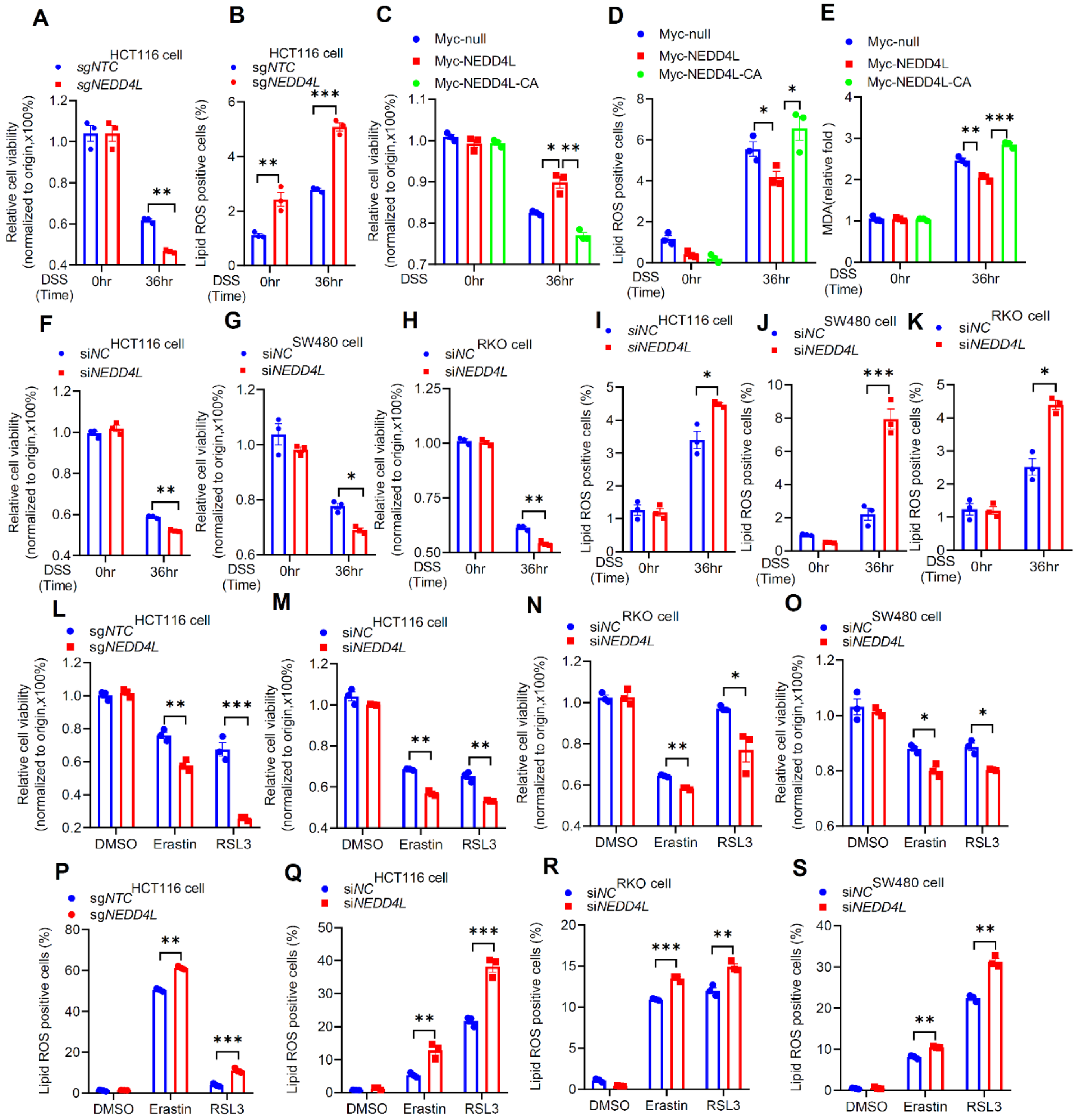

Supplemental Figure 6

A

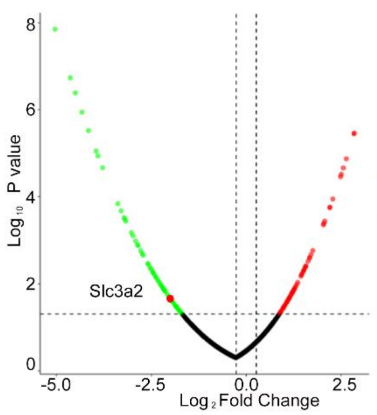

B

Gene	log2FC	Pvalue
Ddb1	-0.3424991	0.4679997
Cct3	0.43382955	0.15369378
Ctnnd1	-1.3378389	0.10744529
Slc3a2	-2.0162554	0.02113614

C

Protein name	Normalized unique peptides Flag-NEDD4L IP # PSMs	Vector IP # PSMs	Difference # PSMs	Rank in MS
Nedd4l	431	0	431	1
Rps27a	9	2	7	22
Ywhaz	6	3	3	81
Slc3a2	2	0	2	156
Cct3	17	16	1	167
Psmc4	7	6	1	179
Ctnnd1	1	0	1	255
Ddb1	1	1	0	441
Tardbp	1	1	0	492

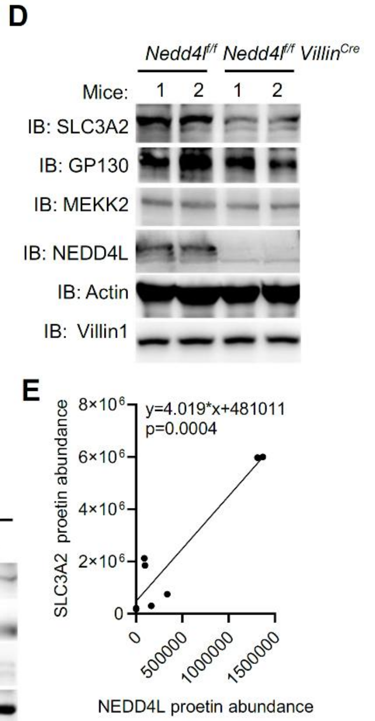

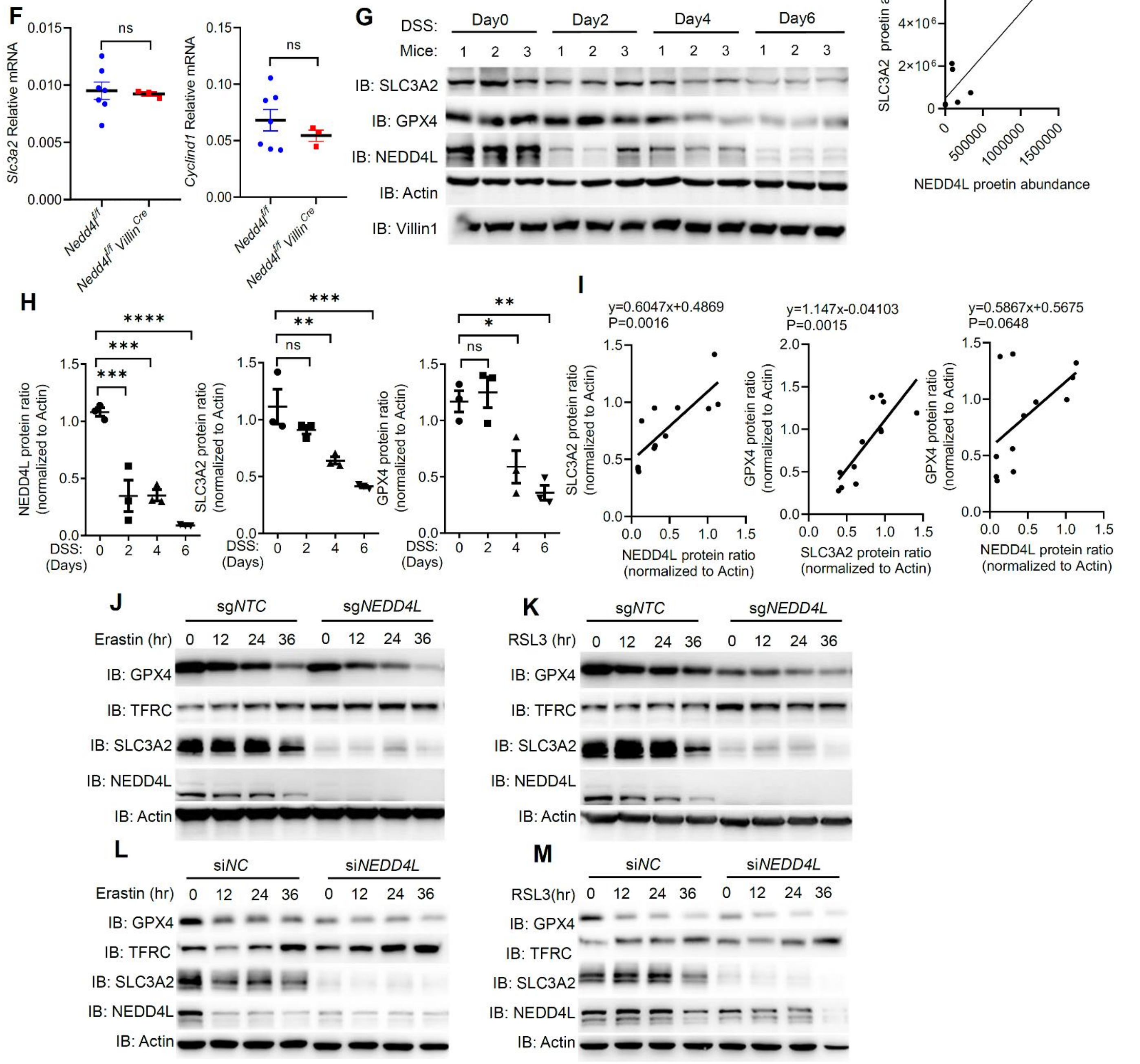

Supplemental Figure 7

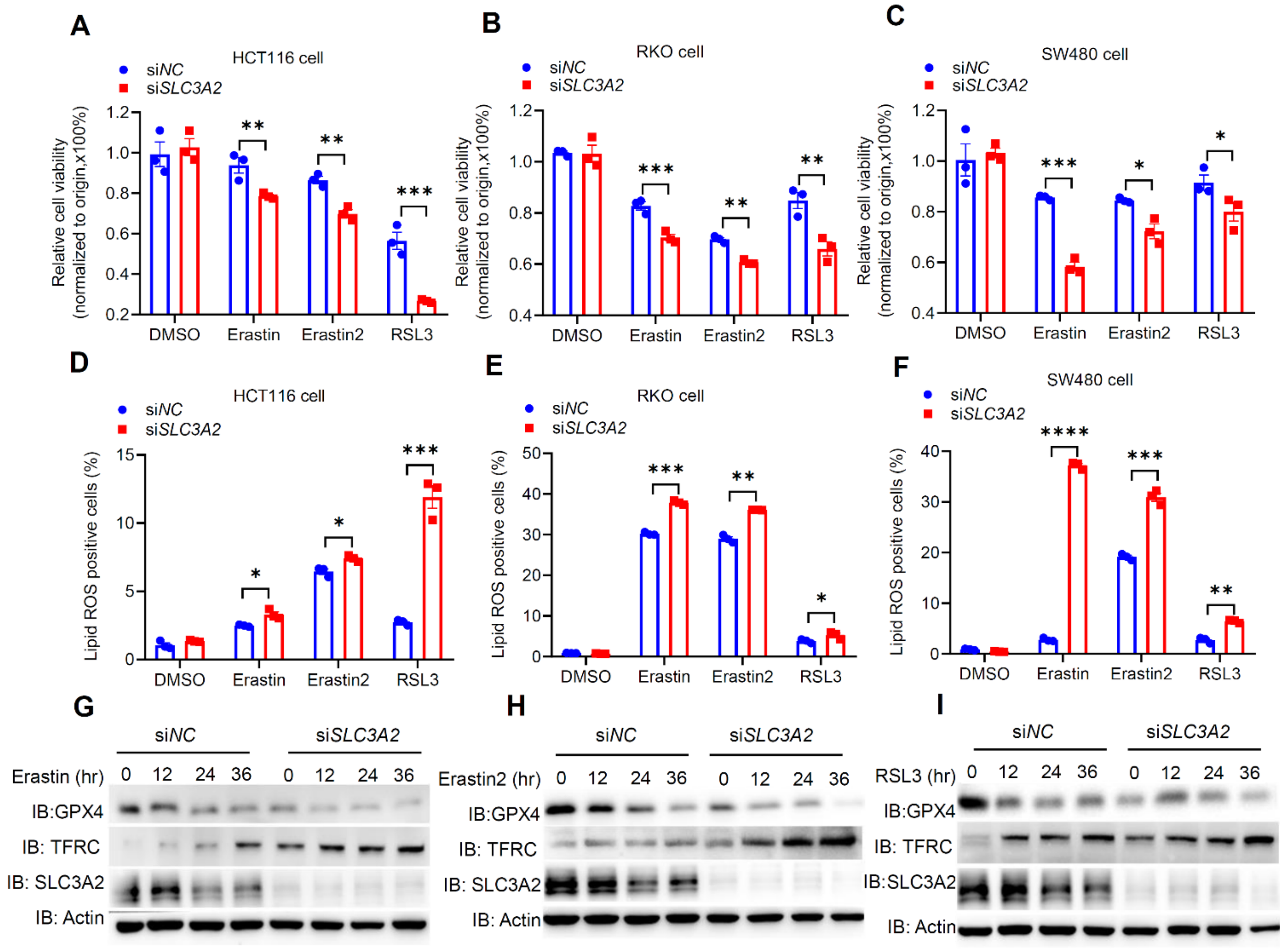

Supplemental Figure 8

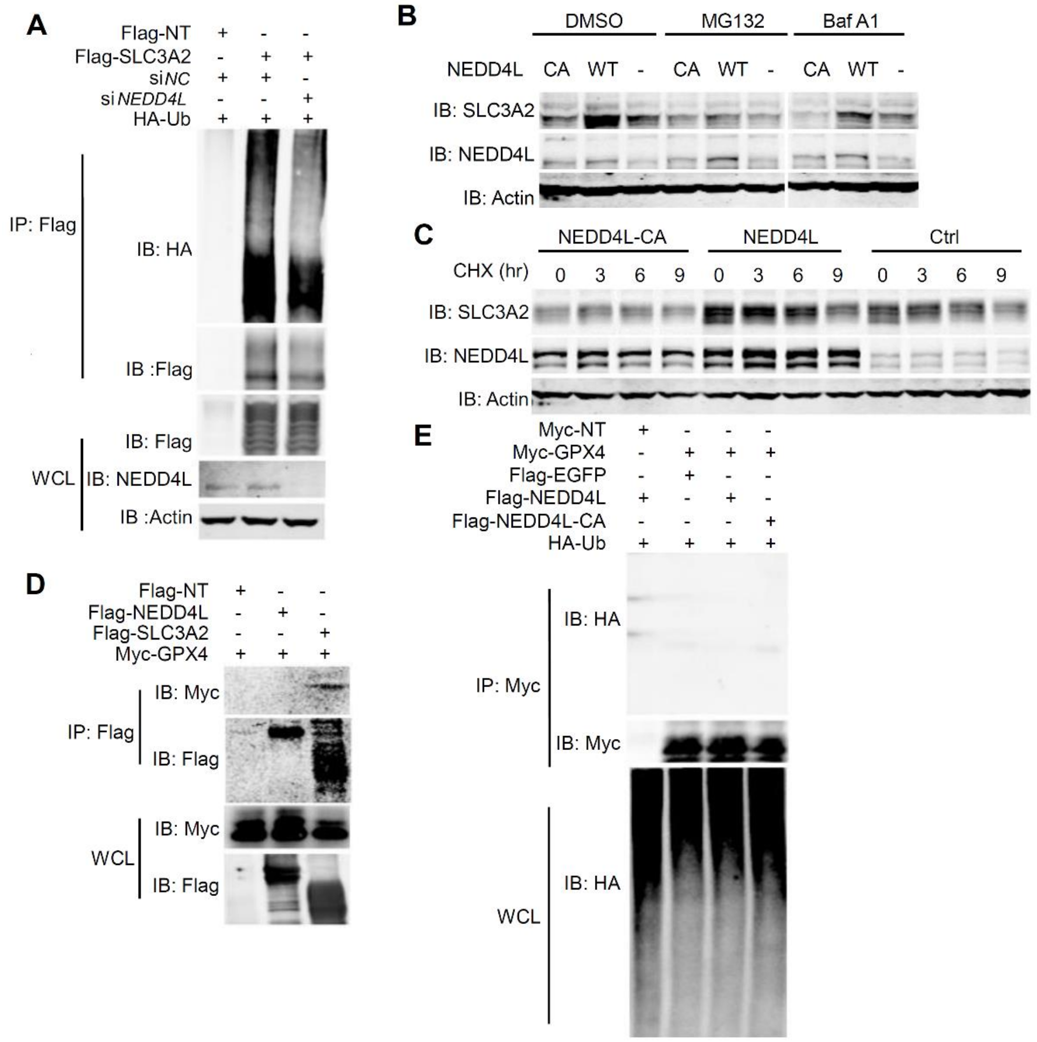

Supplemental Figure 9

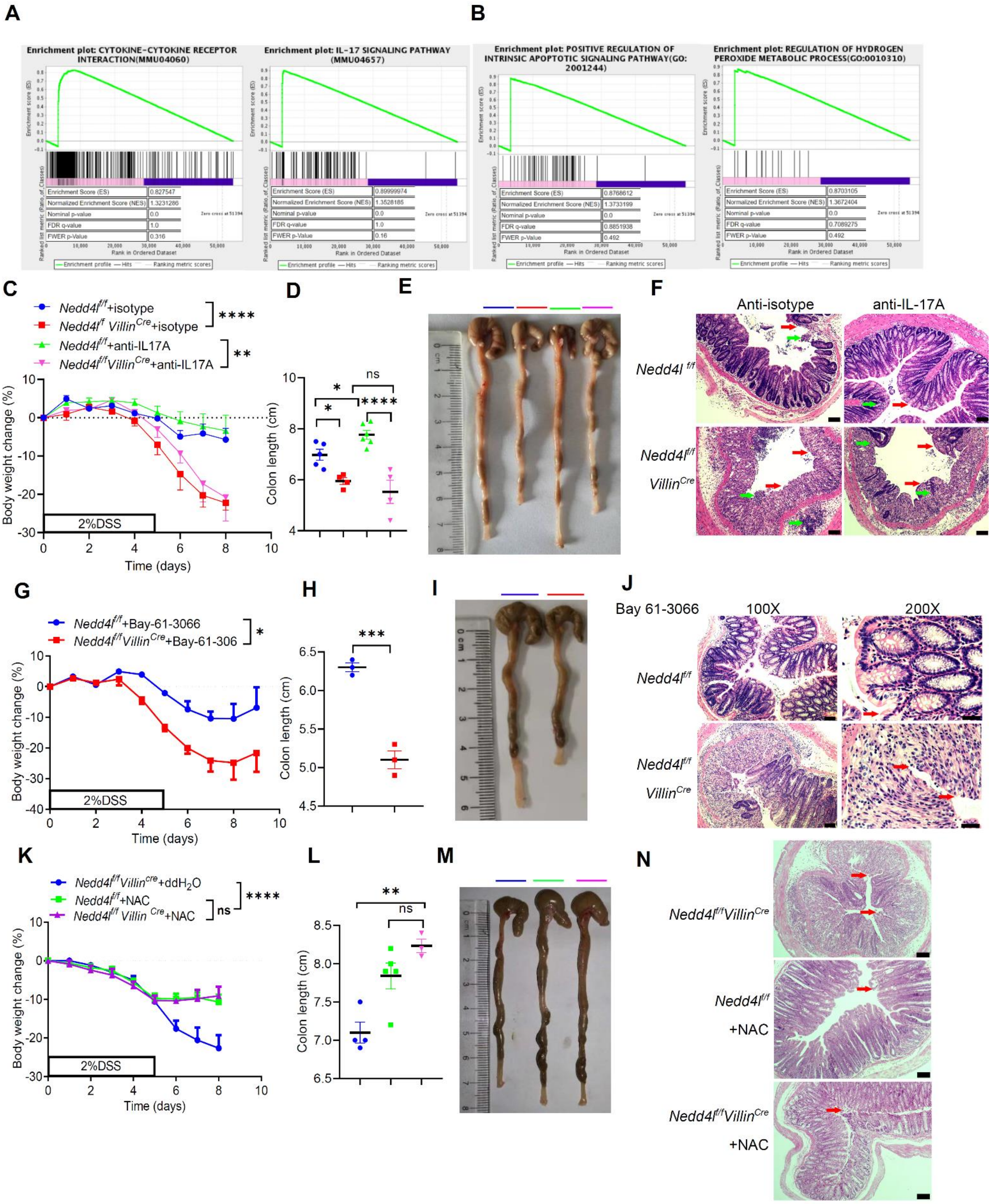

Supplemental Figure 10

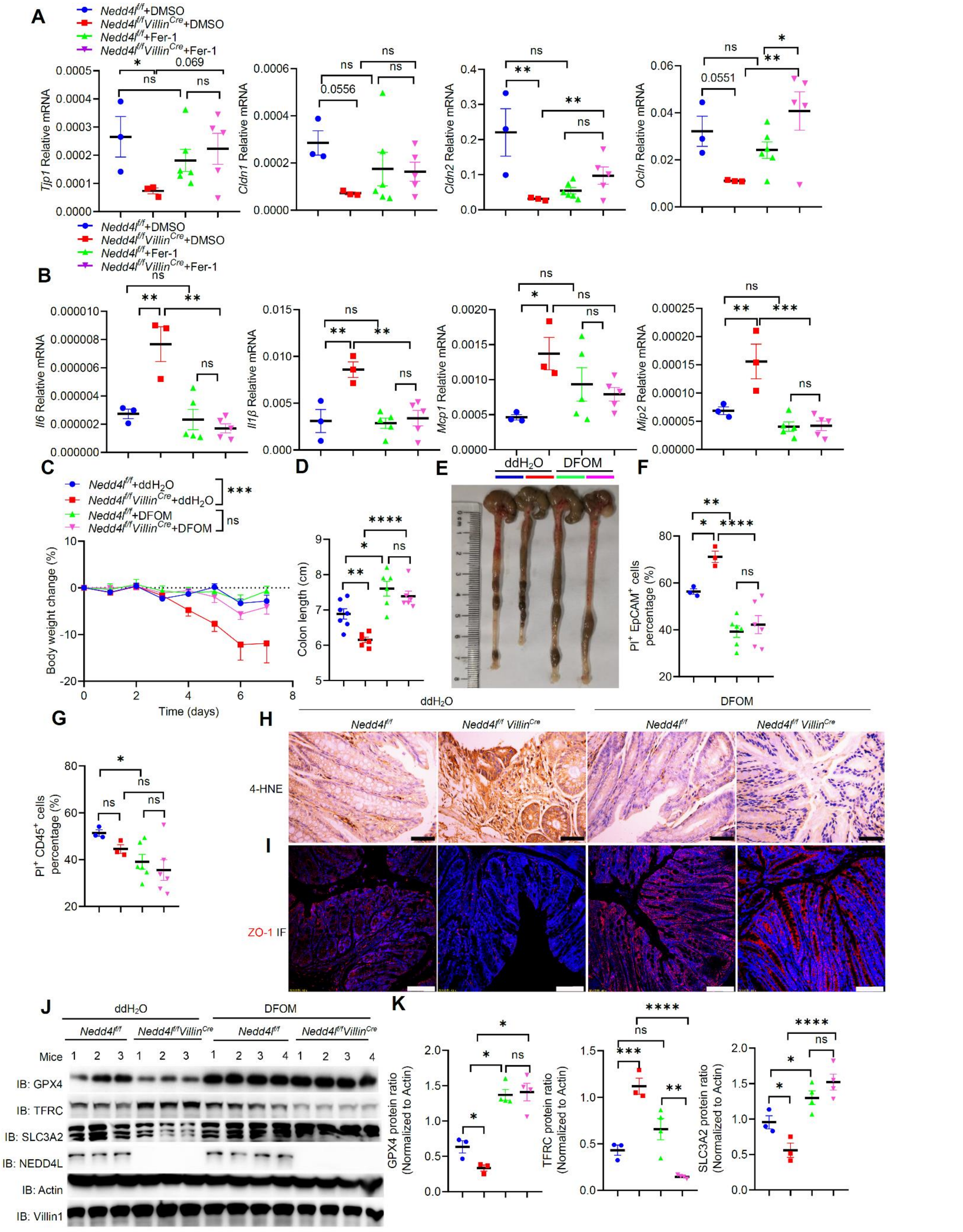

Supplemental Figure 11

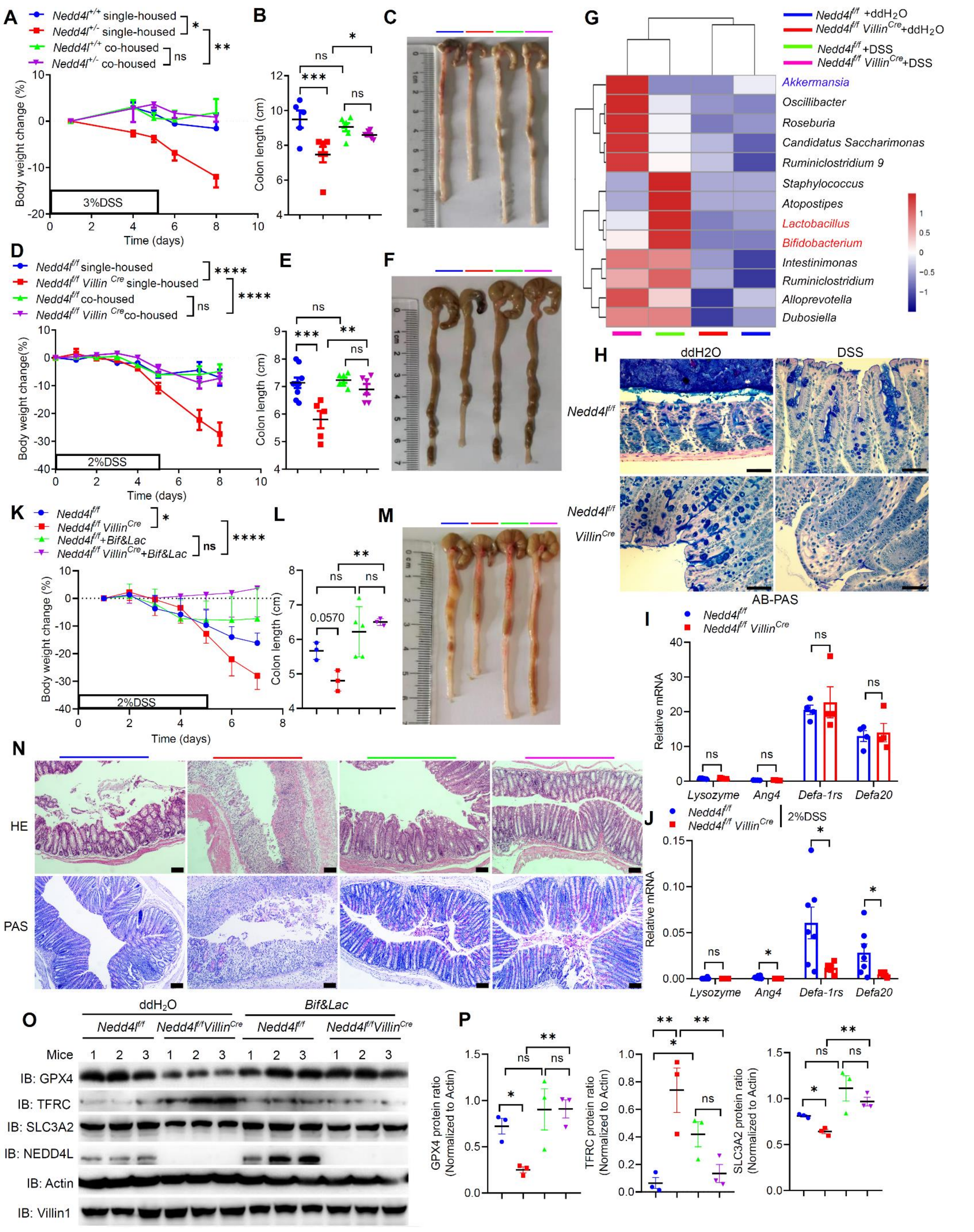

Supplemental Figure 12

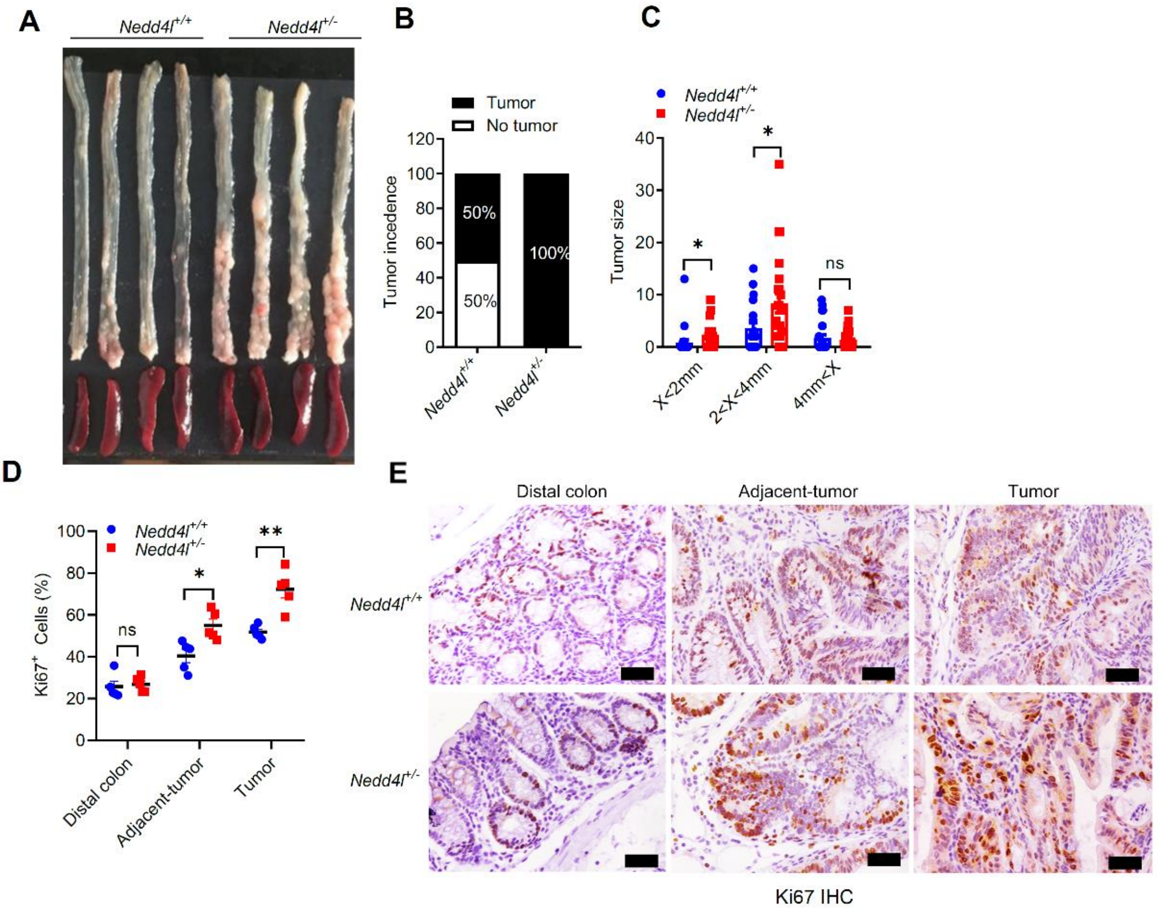

Supplemental Figure 13

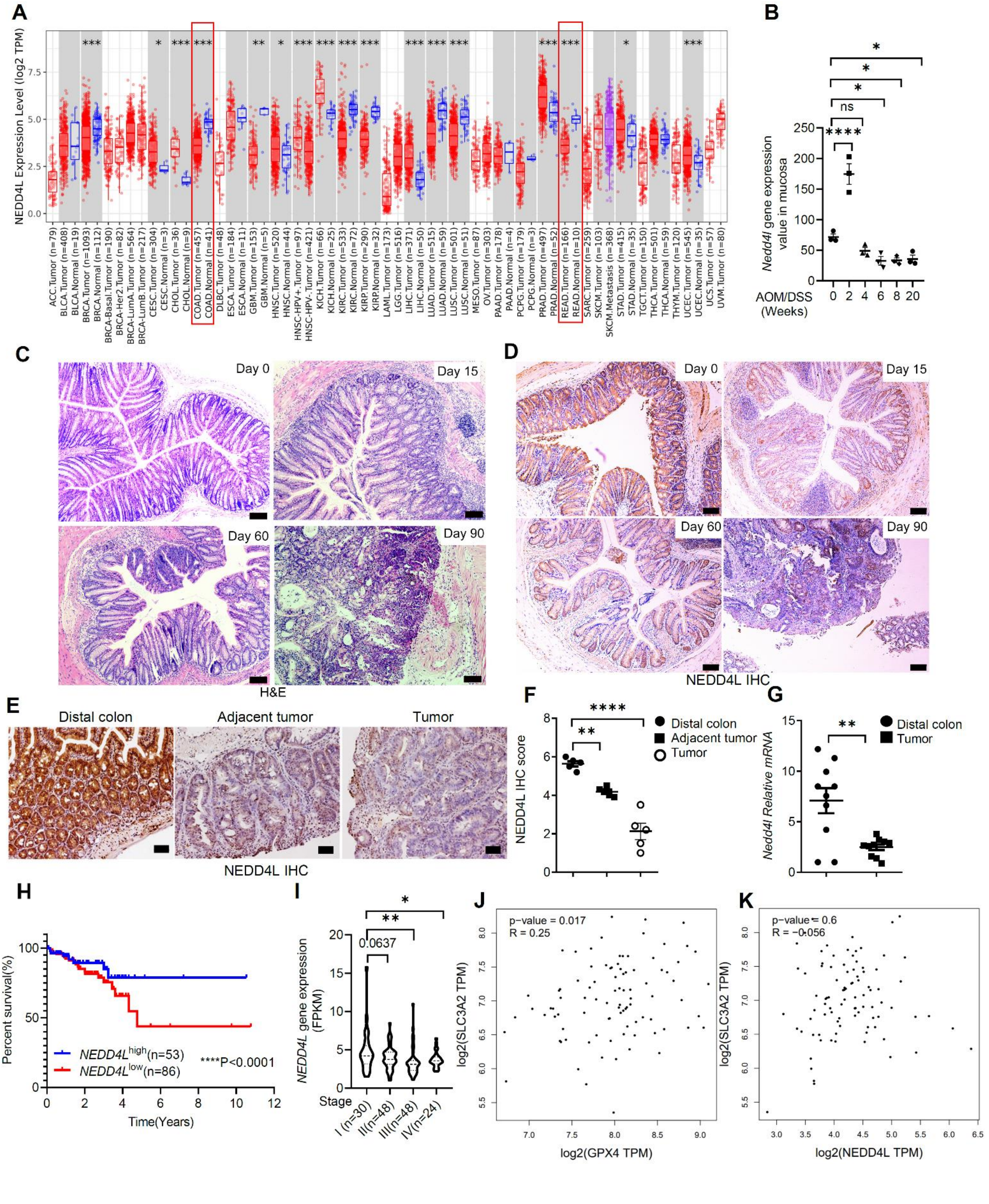

Supplement: Supplemental data [file jci-135-173994-s152.pdf]
