# Supplementary material for: NEDD4L mediates intestinal epithelial cell ferroptosis to restrict inflammatory bowel diseases and colorectal tumorigenesis
Source: J Clin Invest. 2024 Dec 17;135(3):e173994. doi: 10.1172/JCI173994 (PMC11785928; doi:10.1172/JCI173994)

Figure 1H

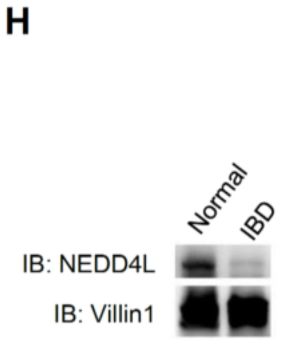

Full unedited gel for Figure 1H

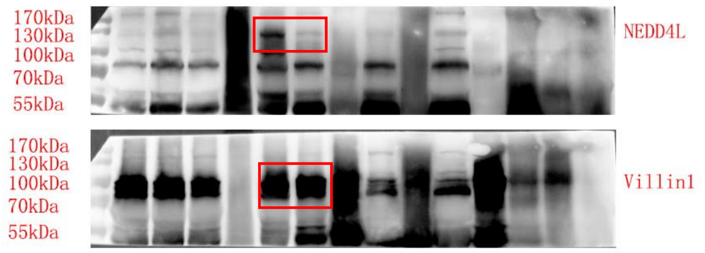

Figure 1I

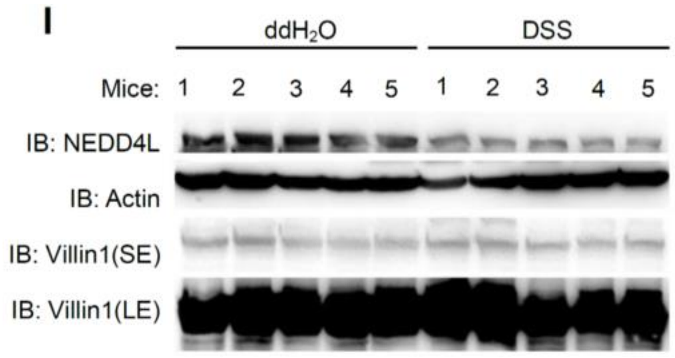

Full unedited gel for Figure 1I

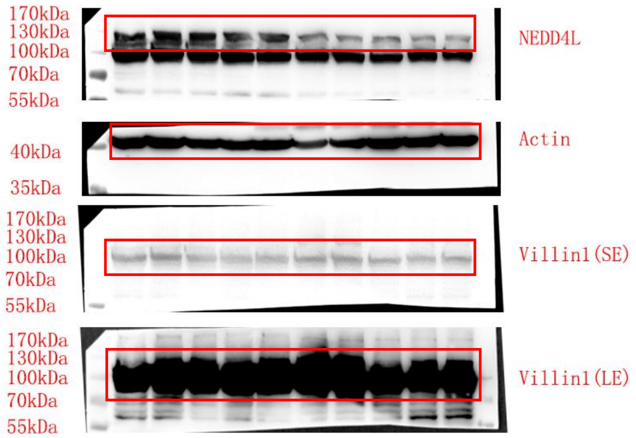

Figure 5D

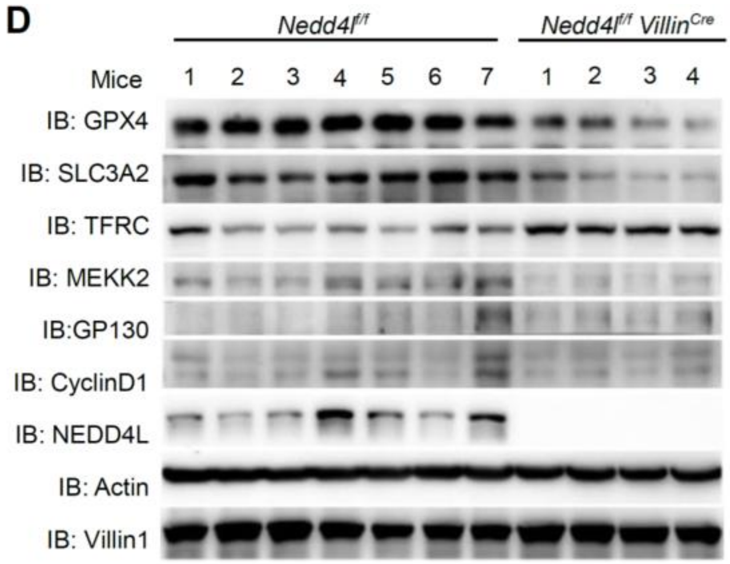

Full unedited gel for Figure 5D

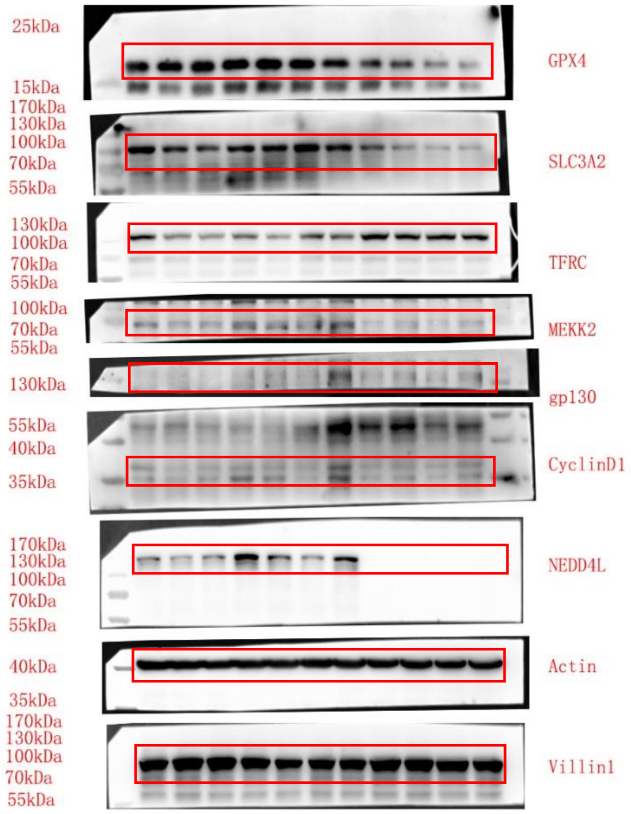

Figure 5H

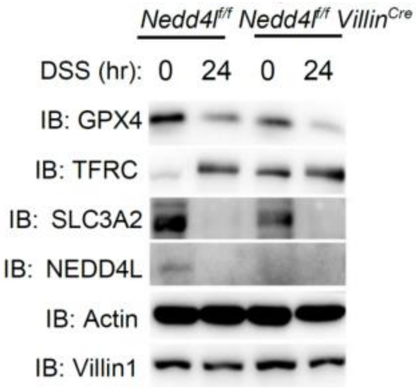

Full unedited gel for Figure 5H

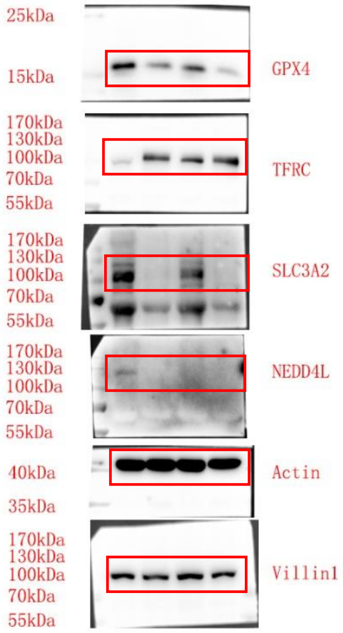

Figure 5I

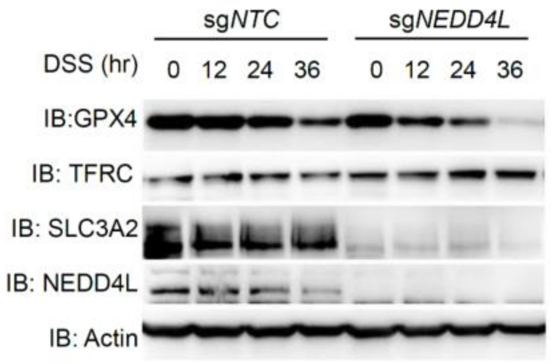

Full unedited gel for Figure 5I

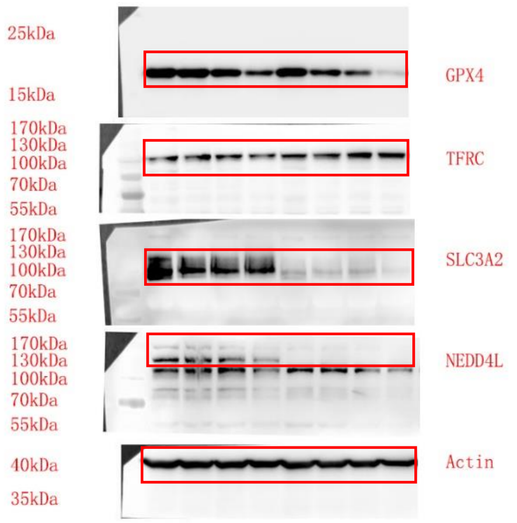

Figure 5J

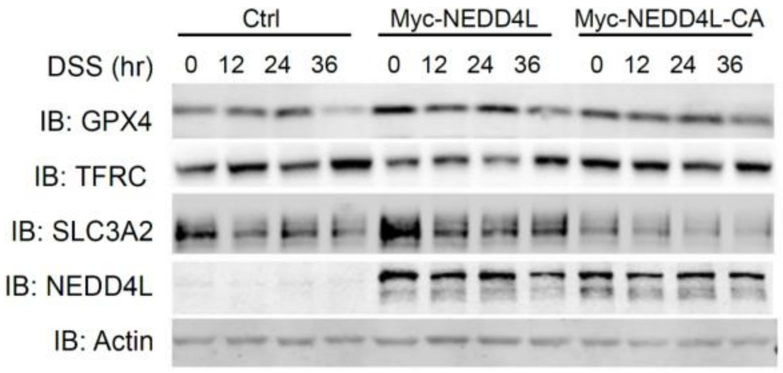

Full unedited gel for Figure 5J

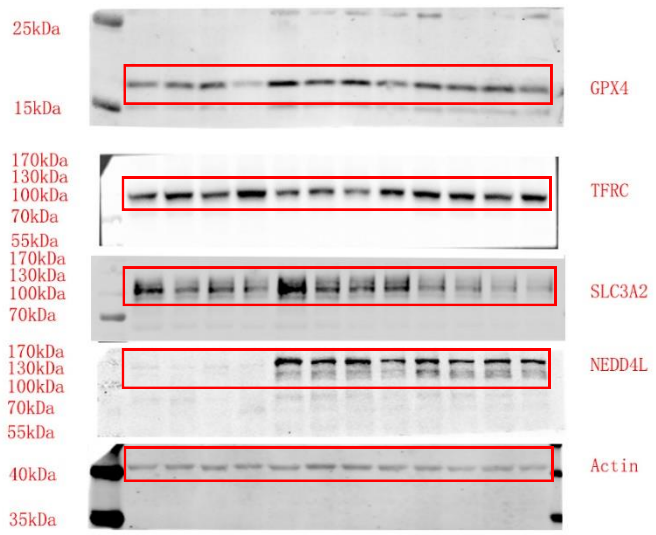

Figure 5K

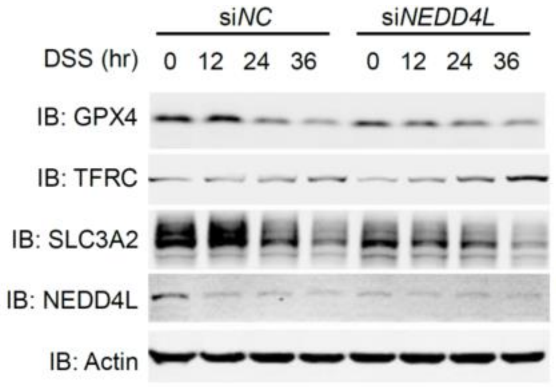

Full unedited gel for Figure 5K

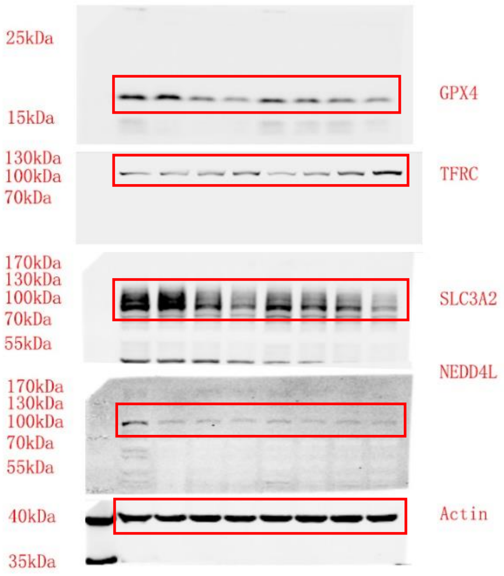

Figure 5L

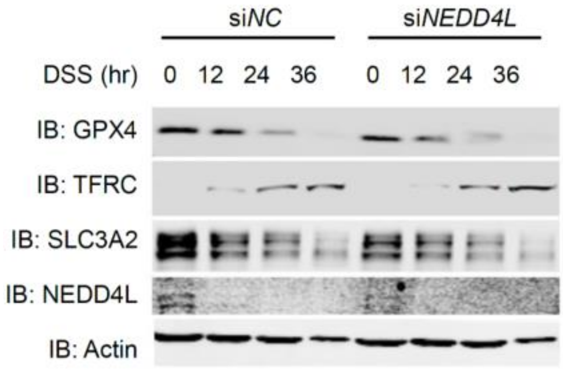

Full unedited gel for Figure 5L

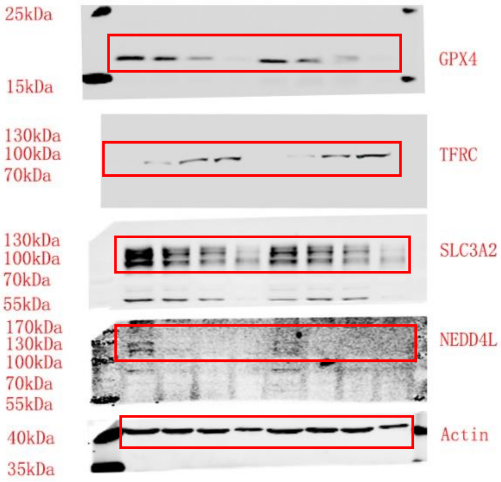

Figure 5M

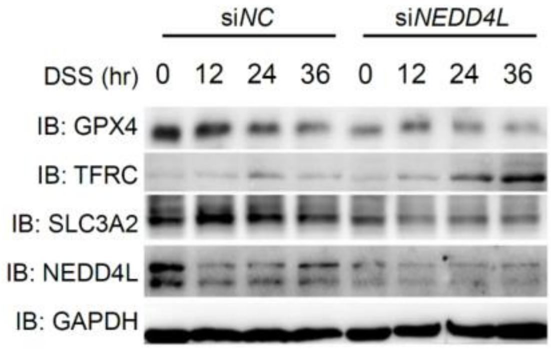

Full unedited gel for Figure 5M

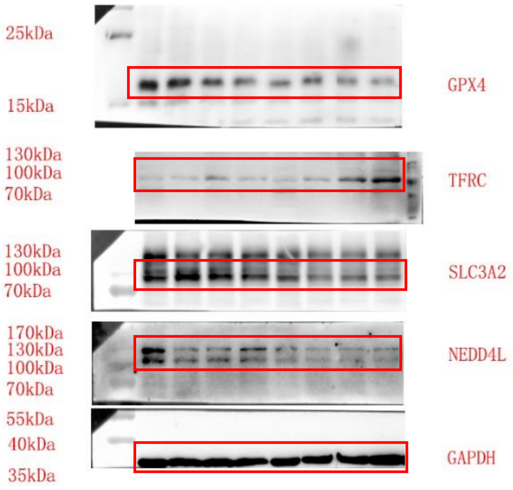

Figure 6G

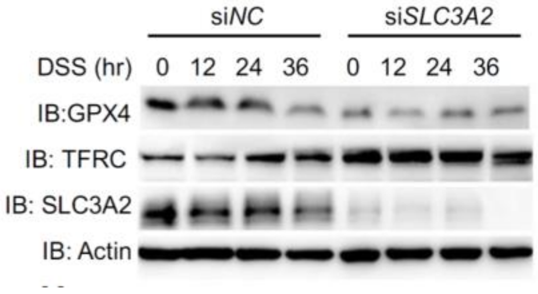

Full unedited gel for Figure 6G

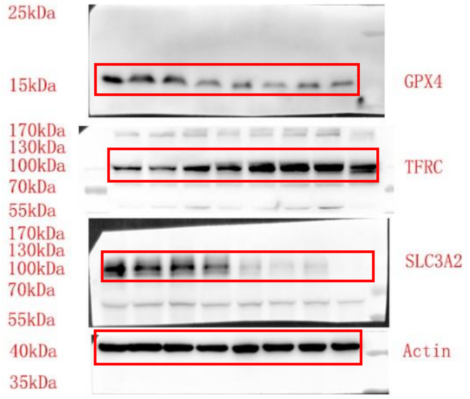

Figure 6H

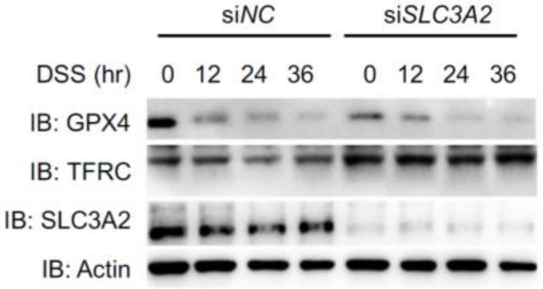

Full unedited gel for Figure 6H

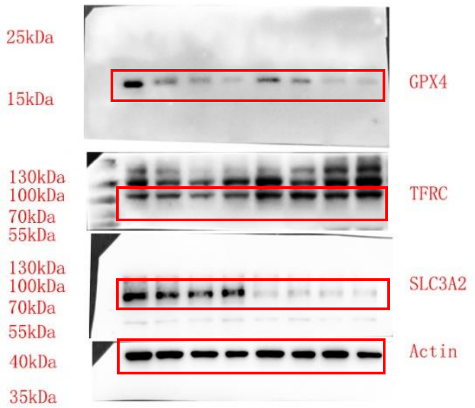

Figure 6I

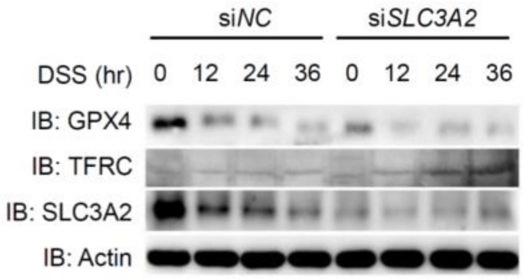

Full unedited gel for Figure 6I

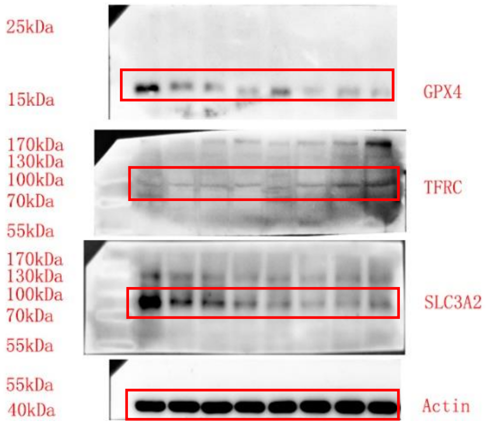

Figure 6M

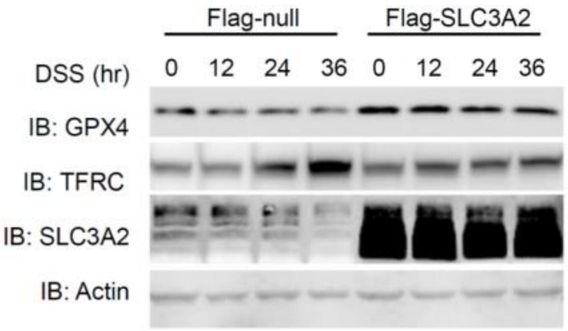

Full unedited gel for Figure 6M

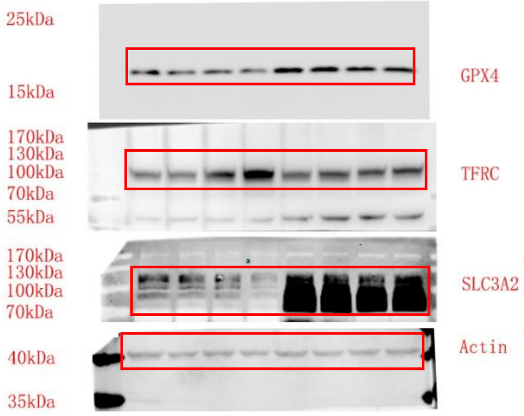

Figure 6P

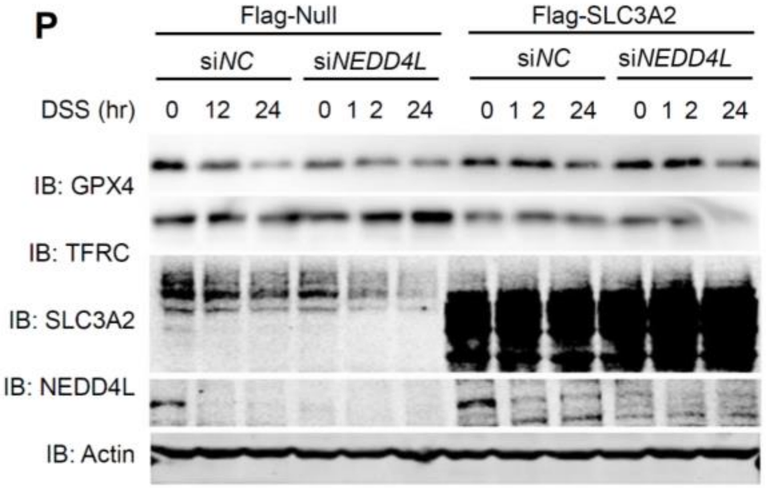

Full unedited gel for Figure 6P

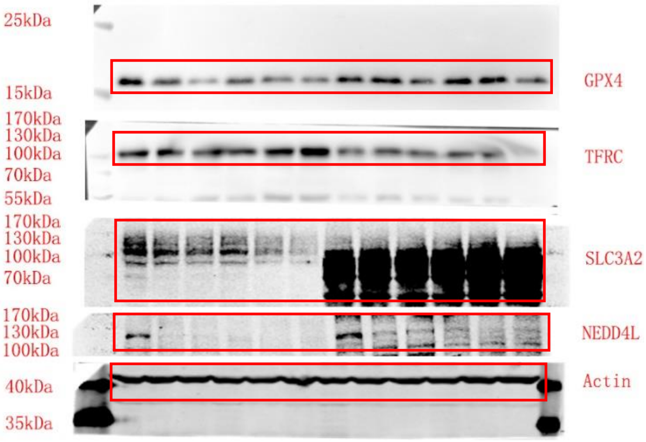

Figure 7A

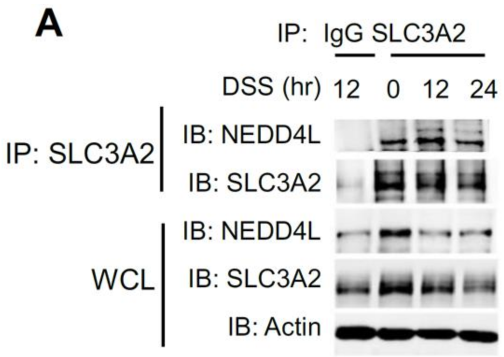

Full unedited gel for Figure 7A

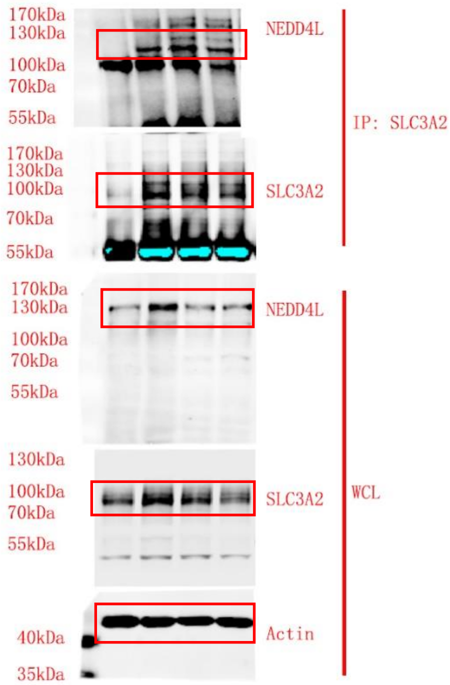

Figure 7B

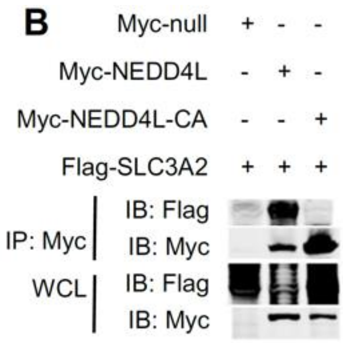

Full unedited gel for Figure 7B

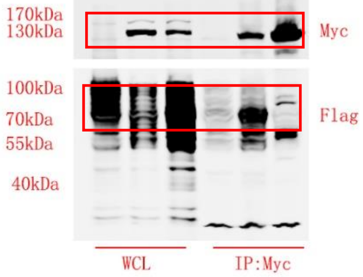

Figure 7C

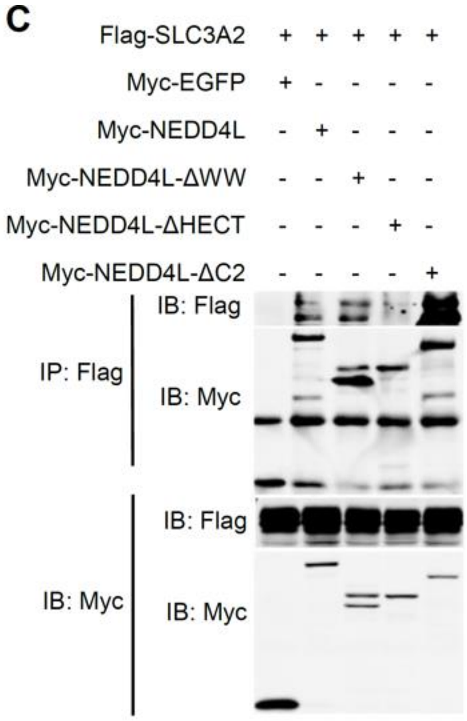

Full unedited gel for Figure 7C

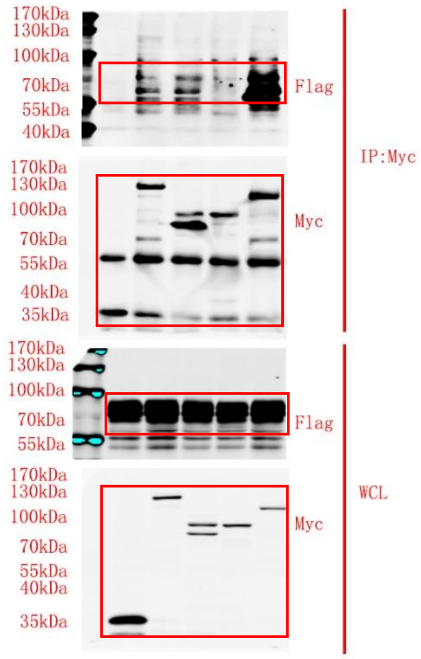

Figure 7D

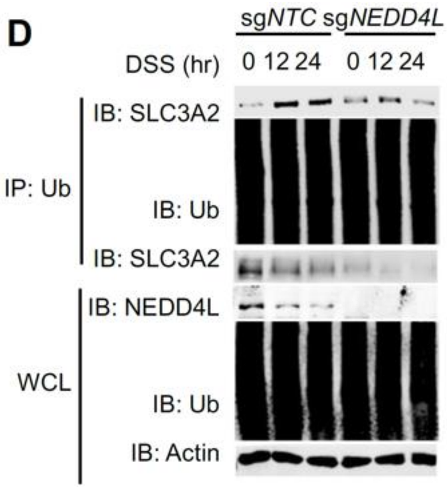

Full unedited gel for Figure 7D

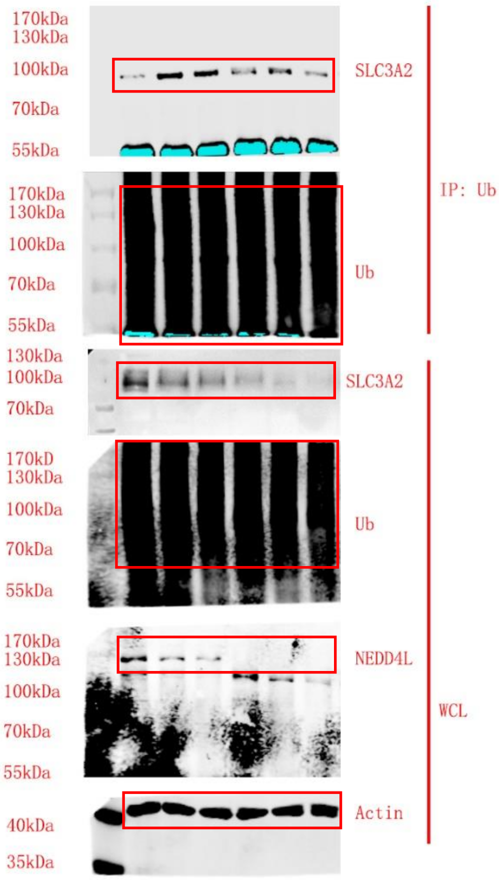

Figure 7E

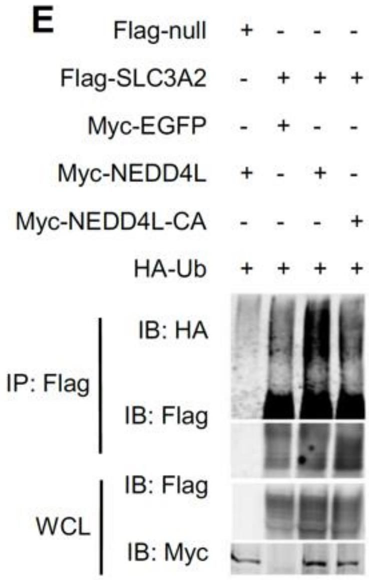

Full unedited gel for Figure 7E

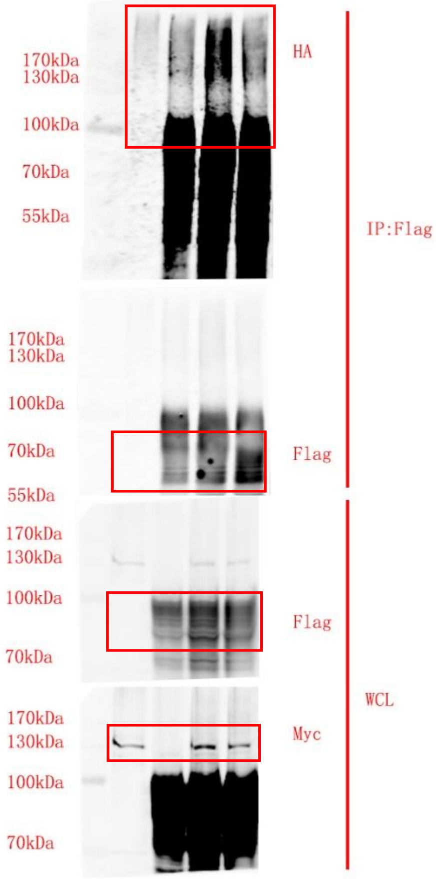

Figure 7F

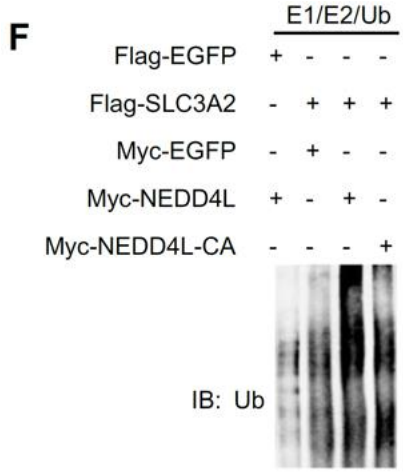

Full unedited gel for Figure 7F

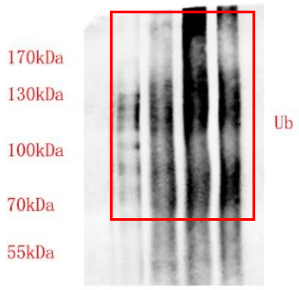

Figure 7G

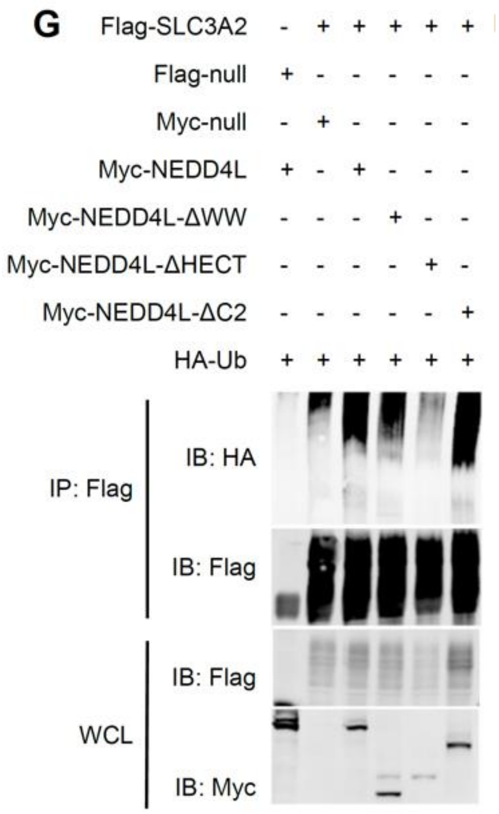

Full unedited gel for Figure 7G

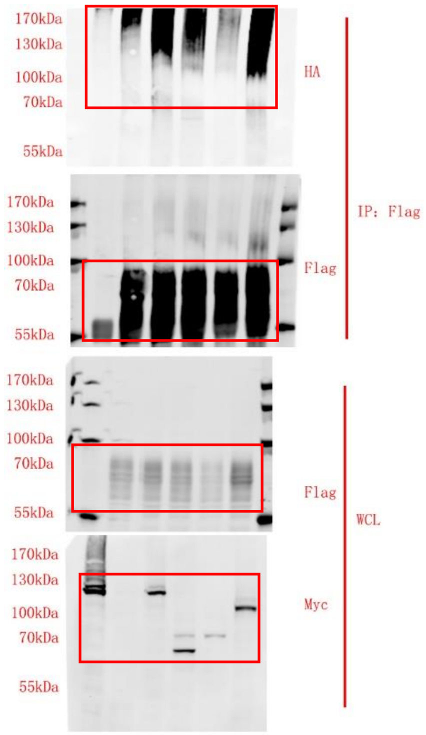

Figure 7H

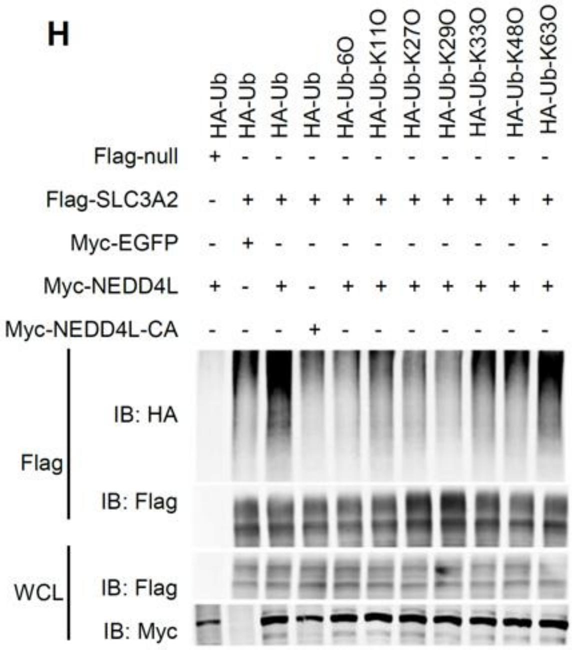

Full unedited gel for Figure 7H

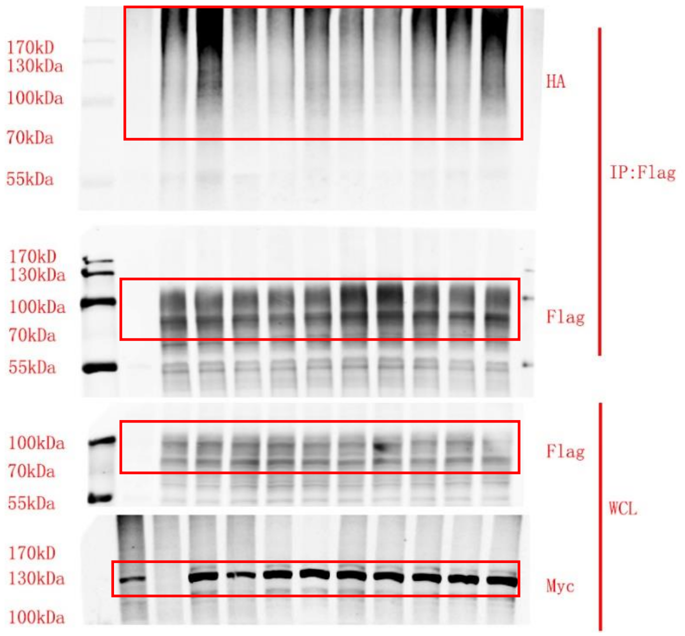

Figure 7I

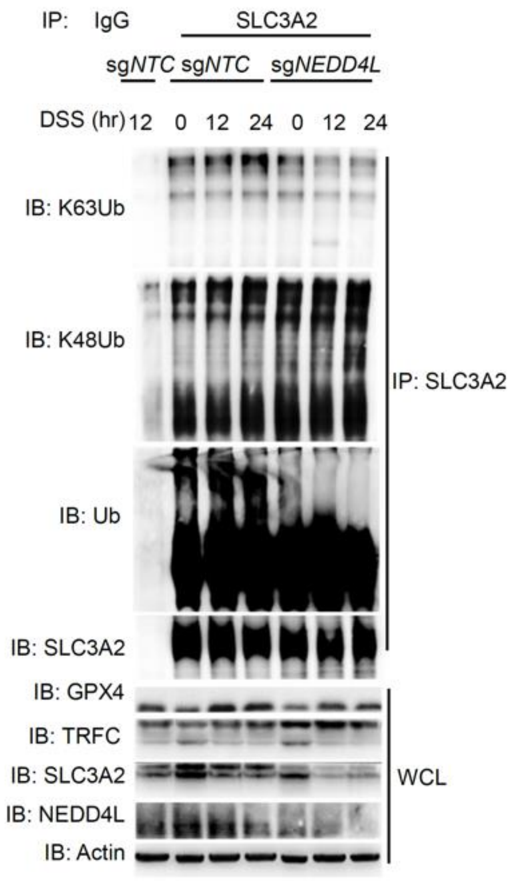

Full unedited gel for Figure 7I

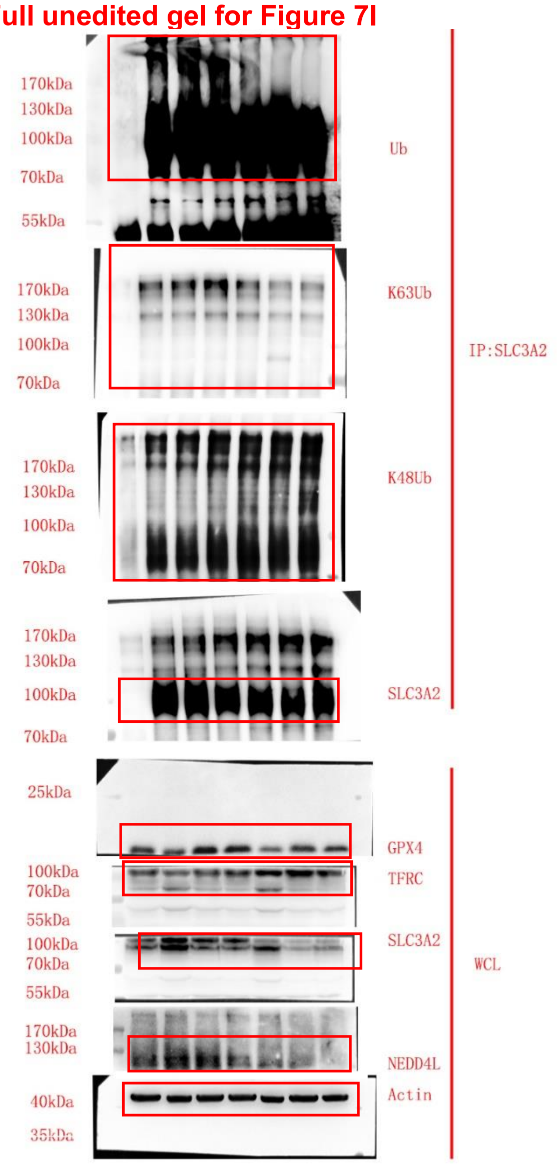

Figure 7J

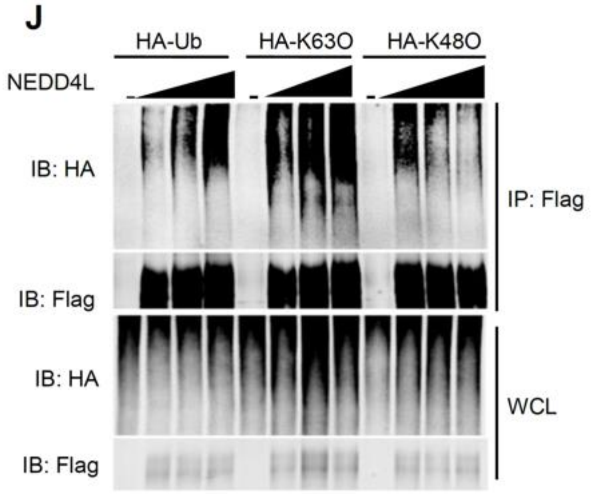

Full unedited gel for Figure 7J

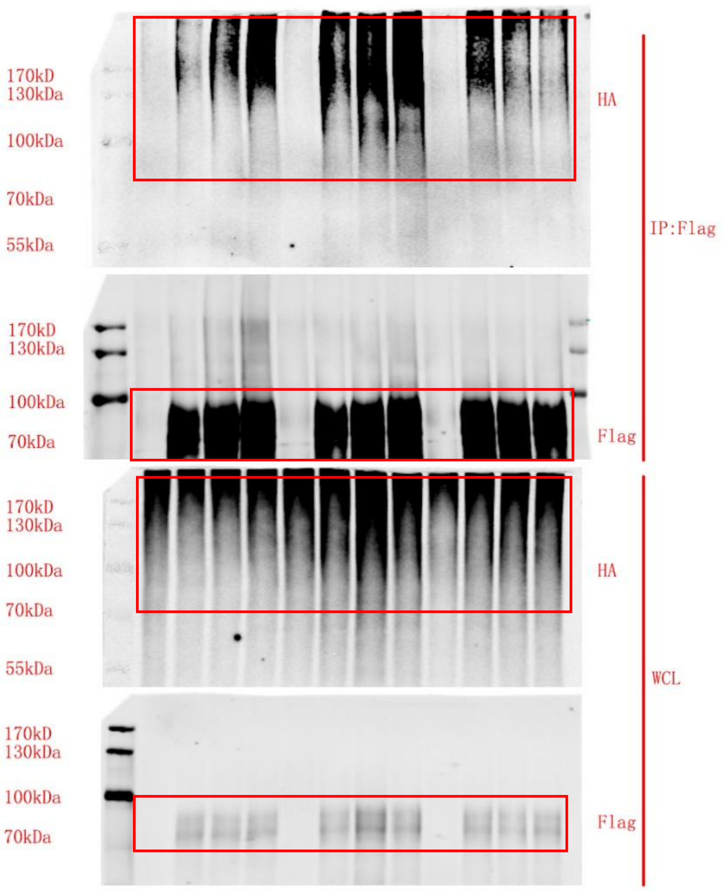

Figure 8L

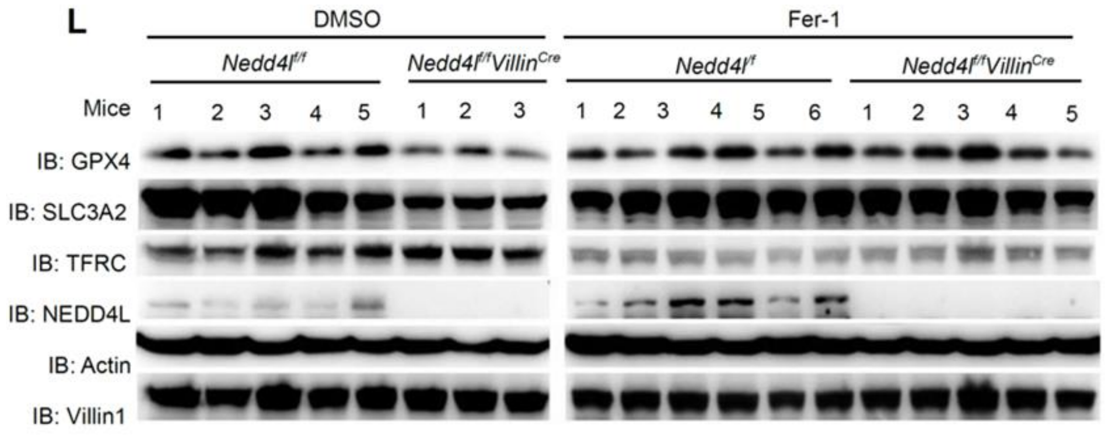

Full unedited gel for Figure 8L

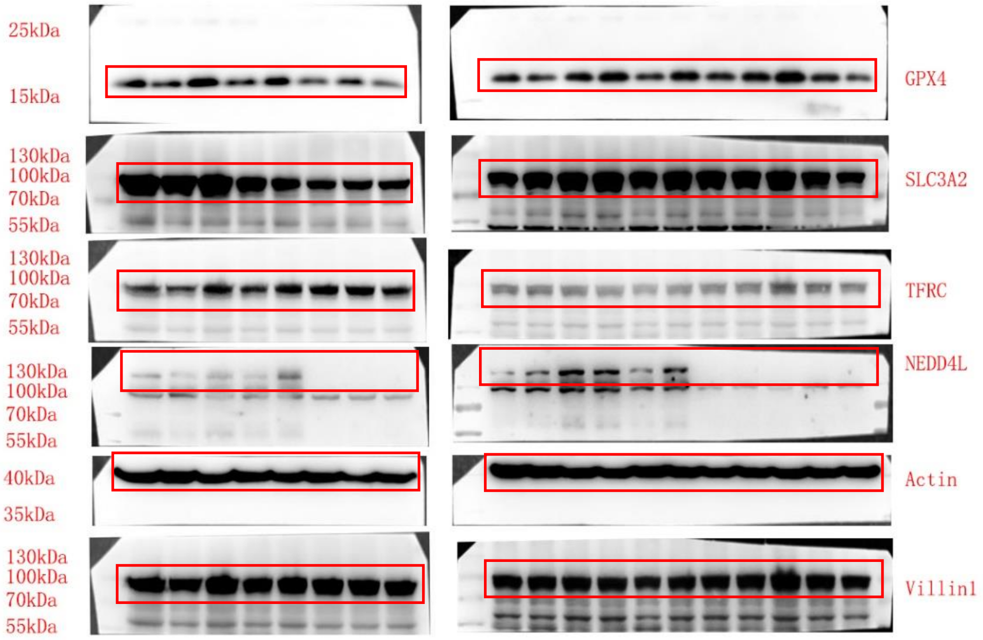

Figure 10A

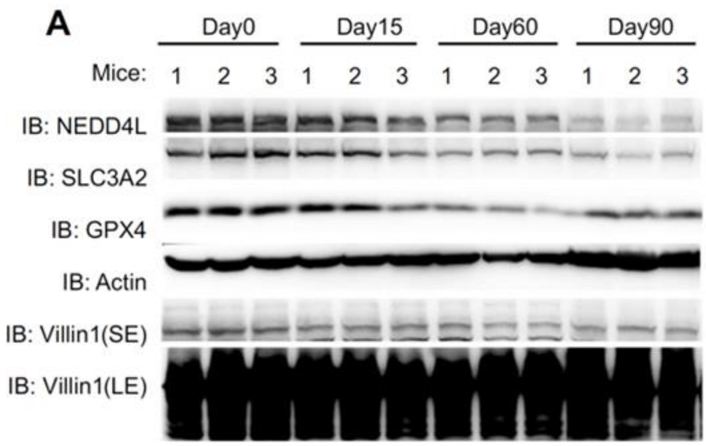

Full unedited gel for Figure 10A

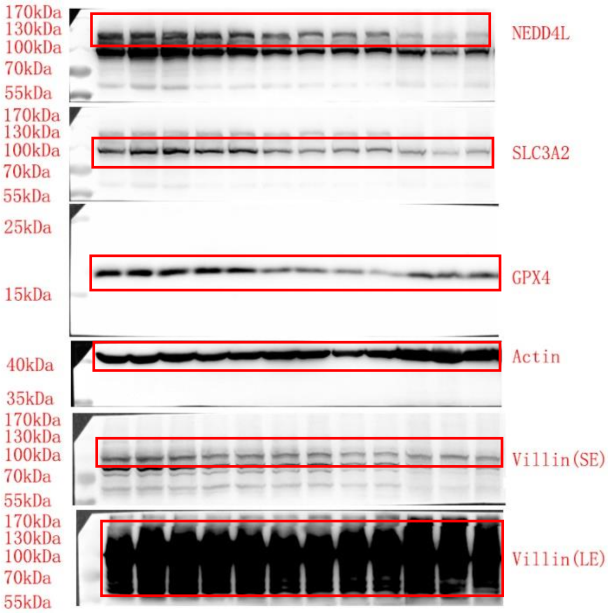

Supplemental Figure 4E

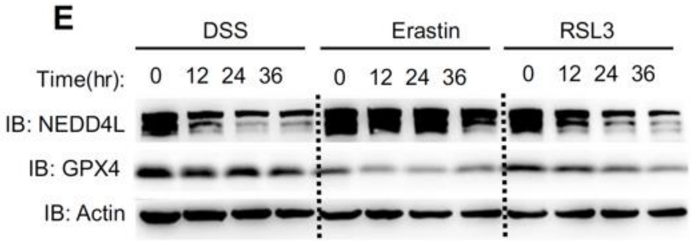

Full unedited gel for Supplemental Figure 4E

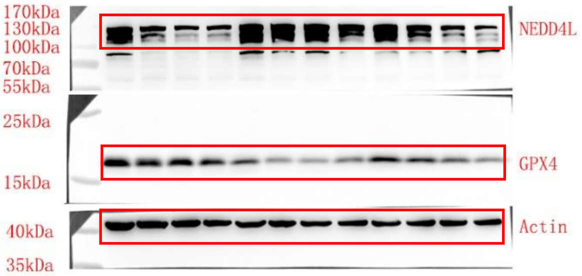

Supplemental Figure 4G

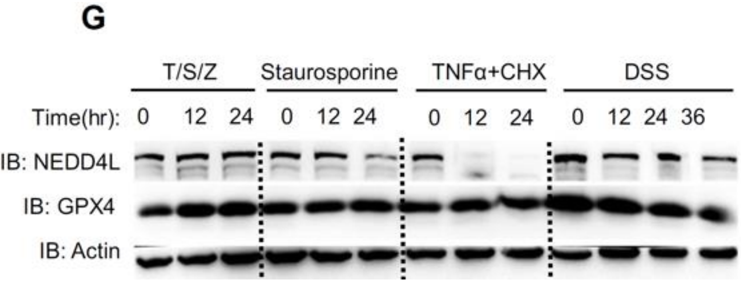

Full unedited gel for Supplemental Figure 4G

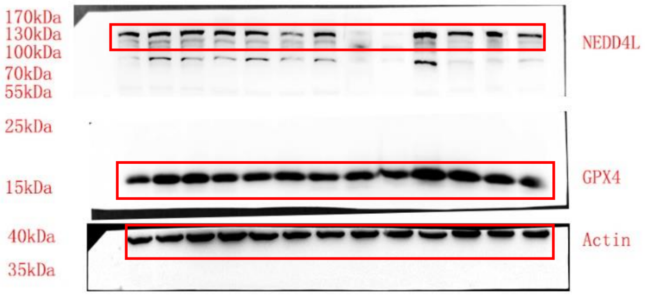

Supplemental Figure 4I

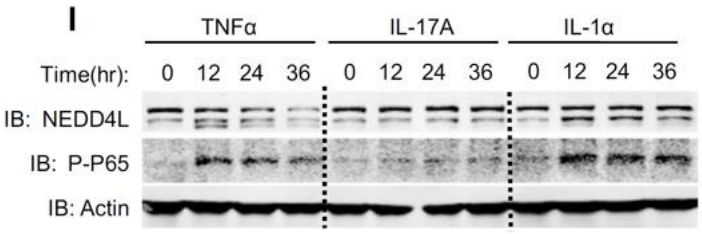

Full unedited gel for Supplemental Figure 4I

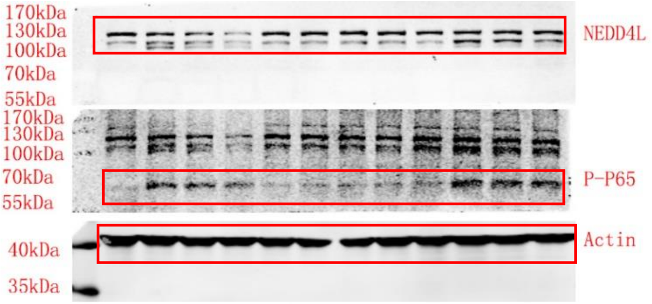

Supplemental Figure 6D

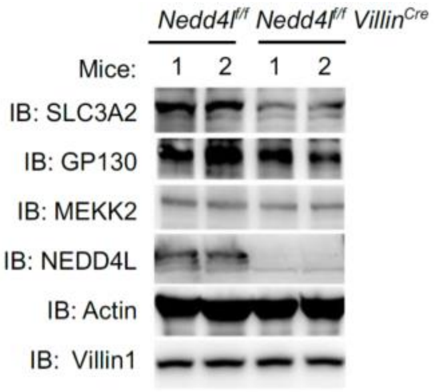

Full unedited gel for Supplemental Figure 6D

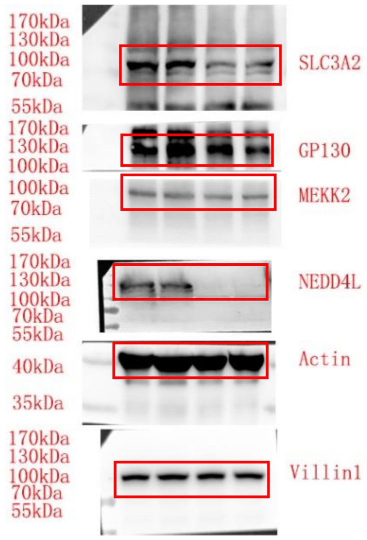

Supplemental Figure 6G

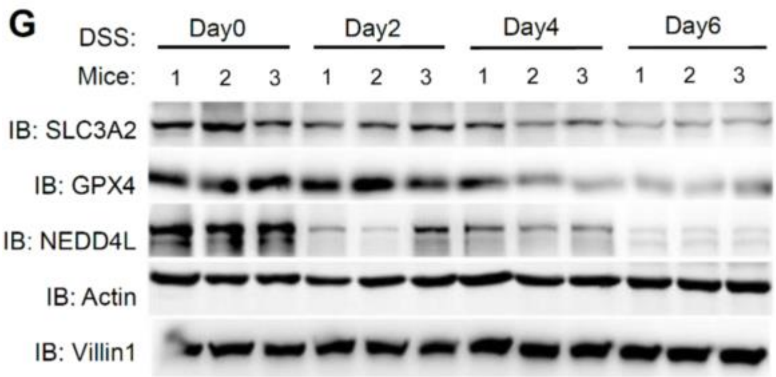

Full unedited gel for Supplemental Figure 6G

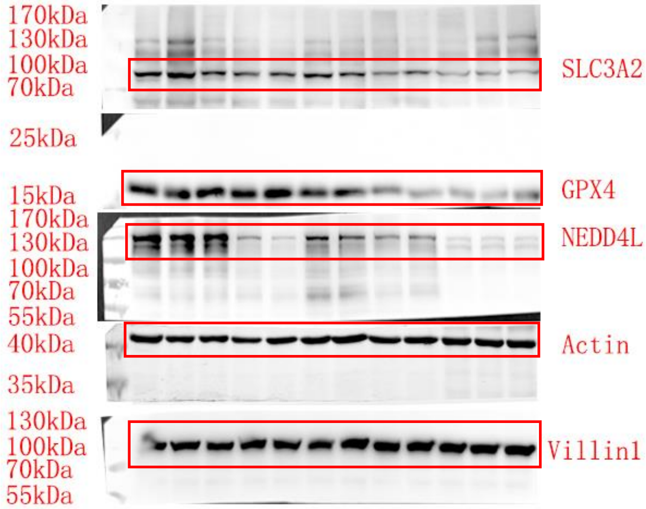

Supplemental Figure 6J

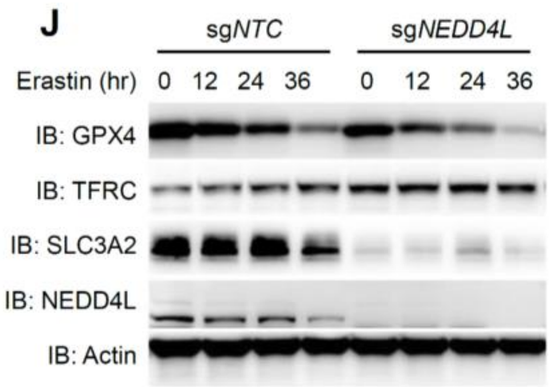

Full unedited gel for Supplemental Figure 6J

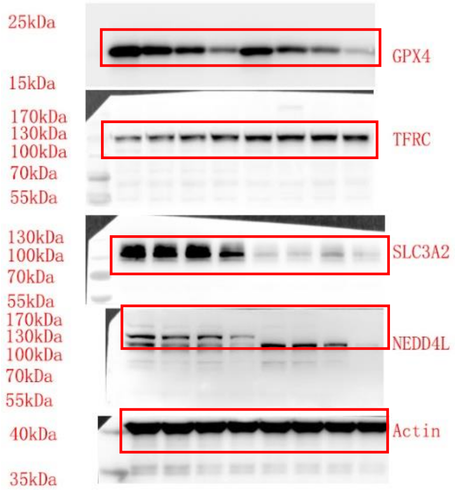

Supplemental Figure 6K

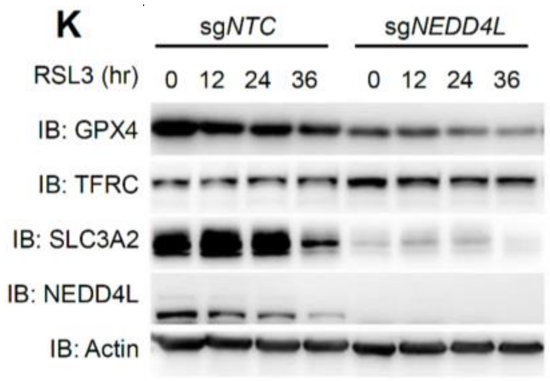

Full unedited gel for Supplemental Figure 6K

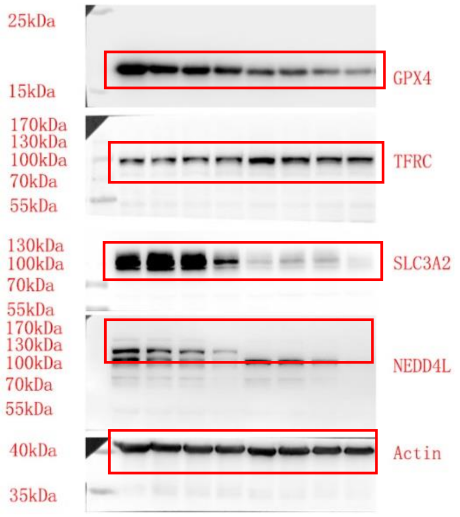

Supplemental Figure 6L

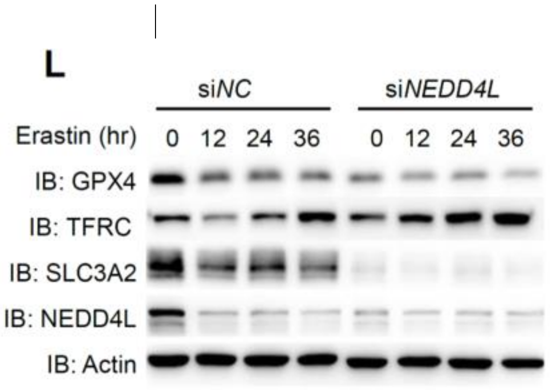

Full unedited gel for Supplemental Figure 6L

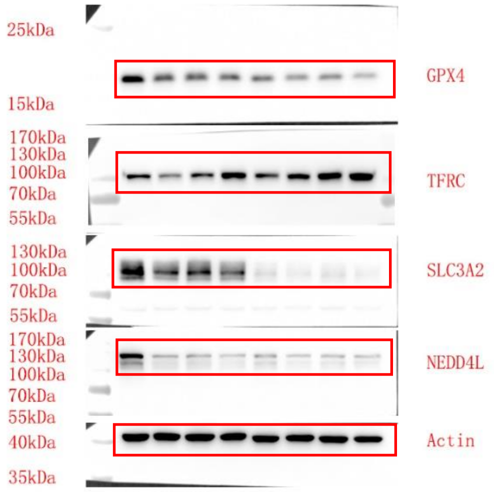

Supplemental Figure 6M

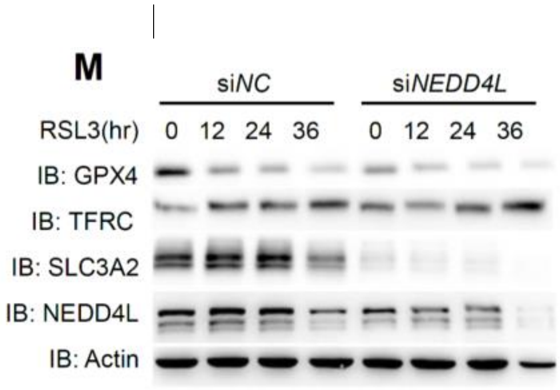

Full unedited gel for Supplemental Figure 6M

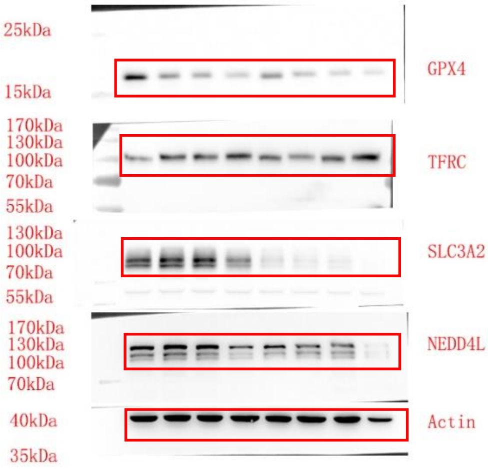

Supplemental Figure 7G

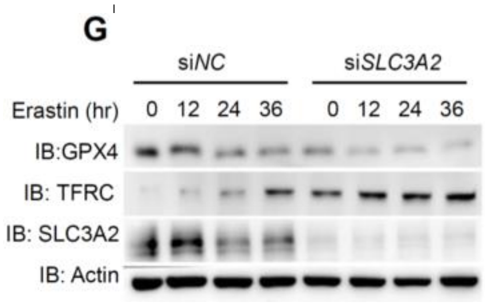

Full unedited gel for Supplemental Figure 7G

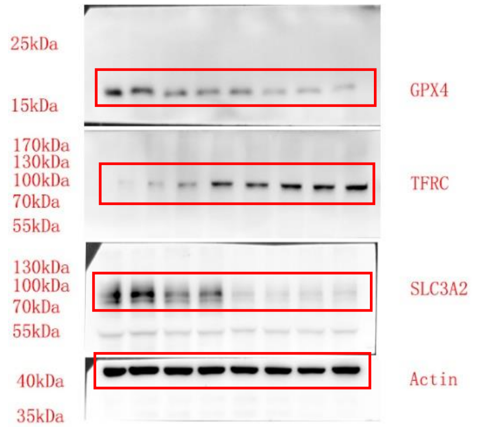

Supplemental Figure 7H

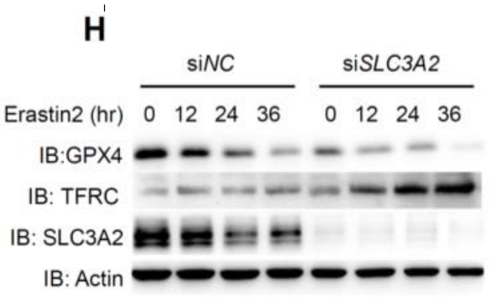

Full unedited gel for Supplemental Figure 7H

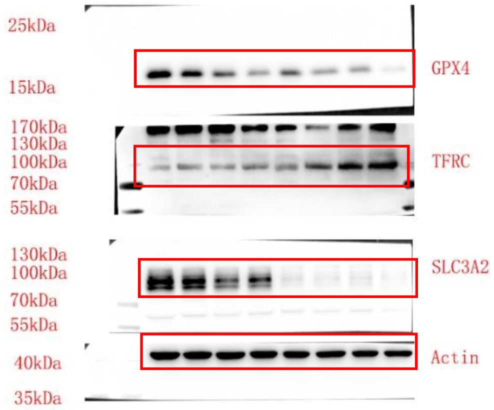

Supplemental Figure 7I

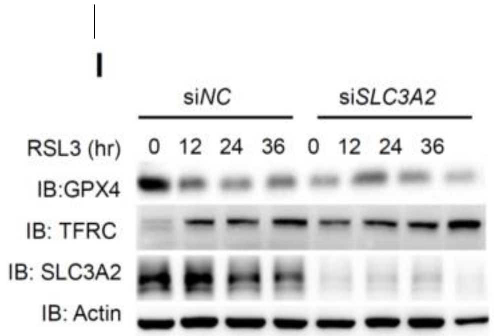

Full unedited gel for Supplemental Figure 7I

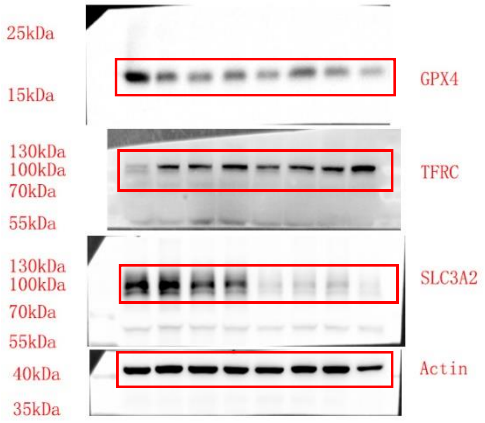

Supplemental Figure 8A

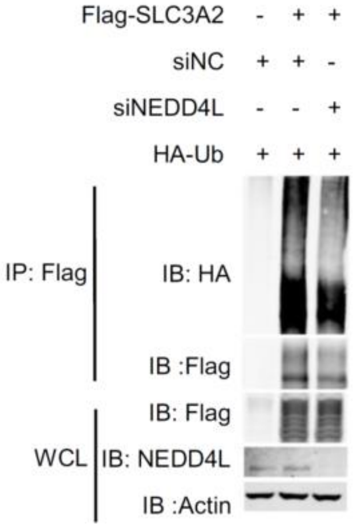

Full unedited gel for Supplemental Figure 8A

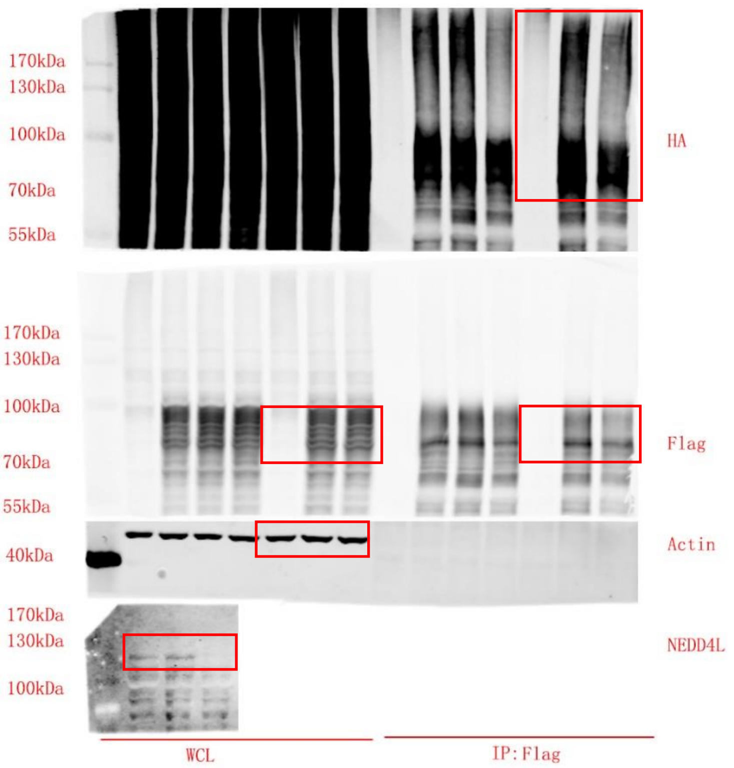

Supplemental Figure 8B

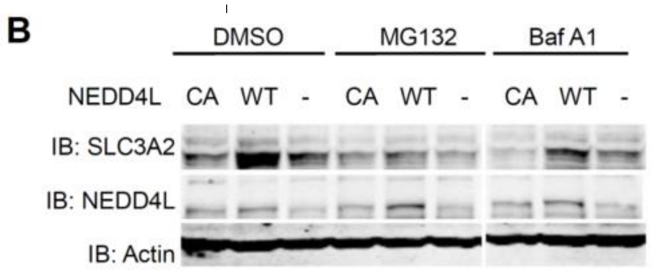

Full unedited gel for Supplemental Figure 8B

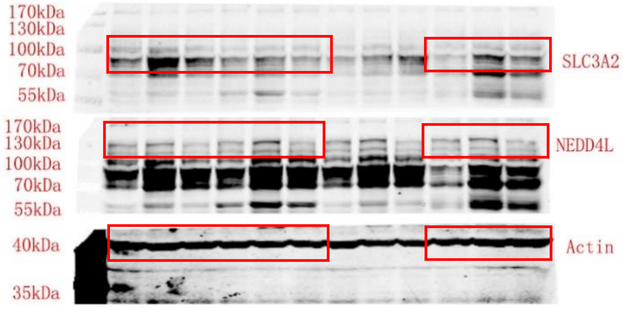

Supplemental Figure 8C

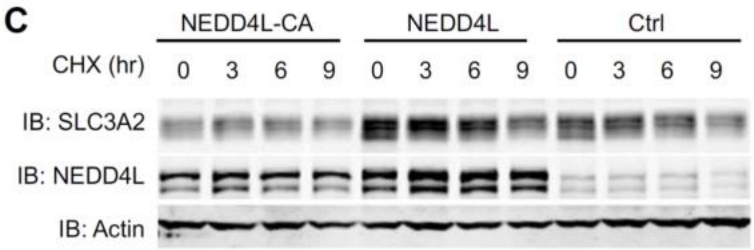

Full unedited gel for Supplemental Figure 8C

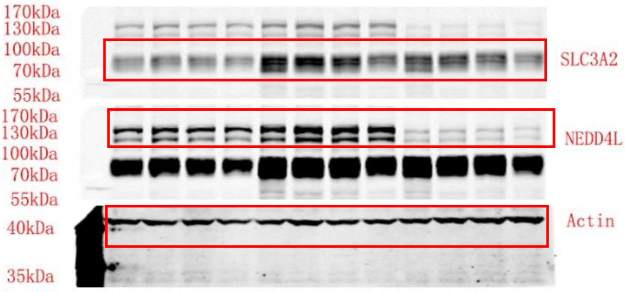

Supplemental Figure 8D

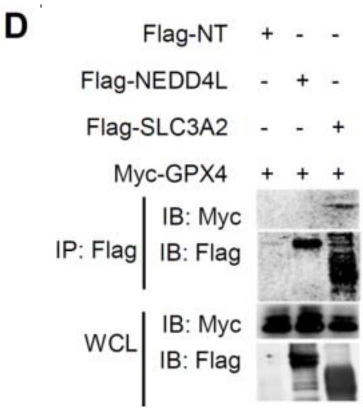

Full unedited gel for Supplemental Figure 8D

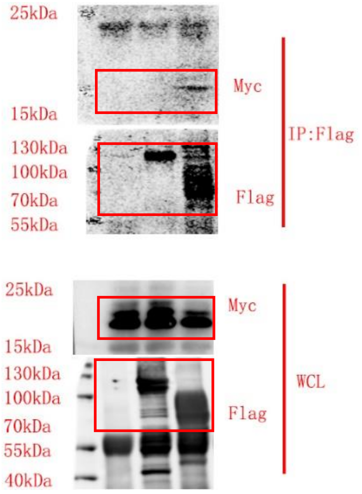

Supplemental Figure 8E

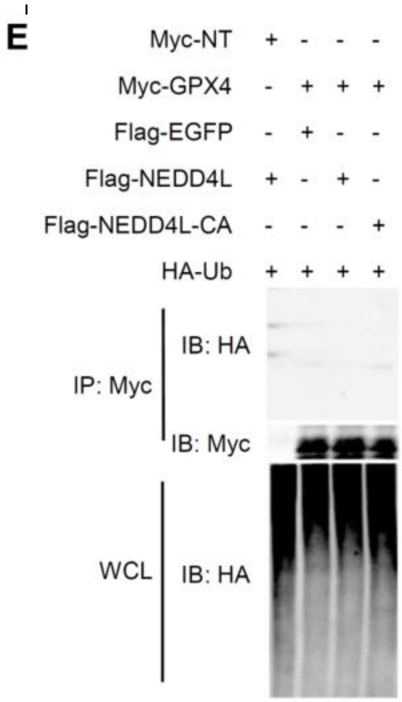

Full unedited gel for Supplemental Figure 8E

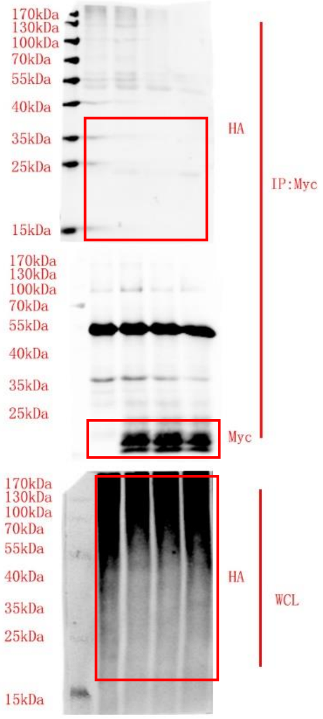

Supplemental Figure 10J

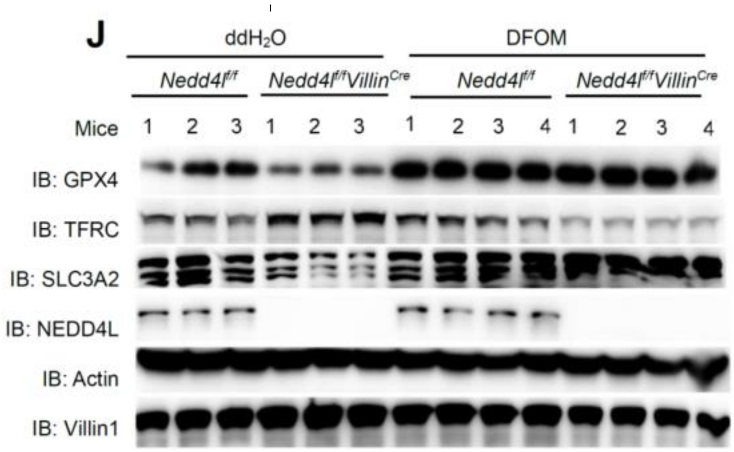

Full unedited gel for Supplemental Figure 10J

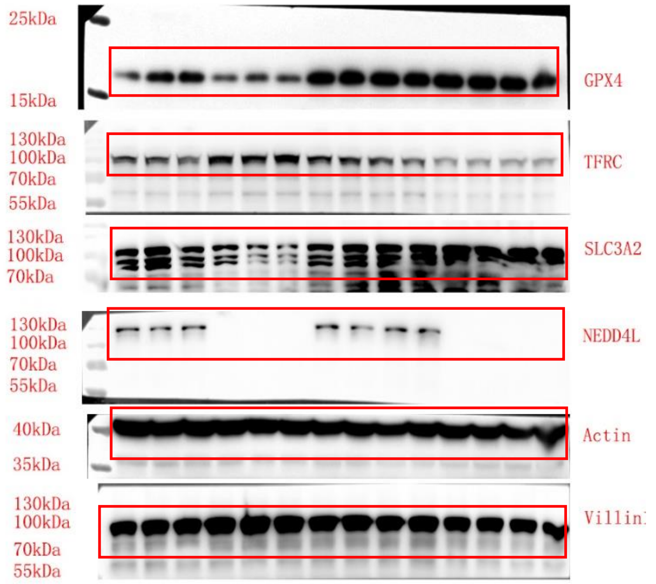

Supplemental Figure 11M

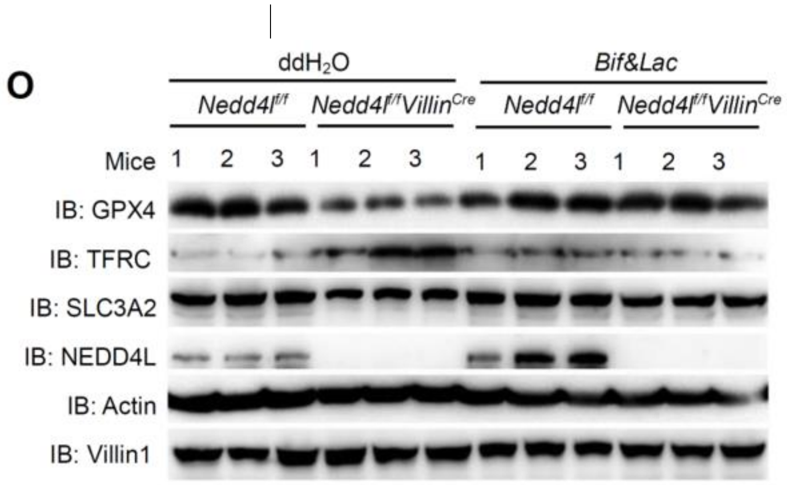

Full unedited gel for Supplemental Figure 11M

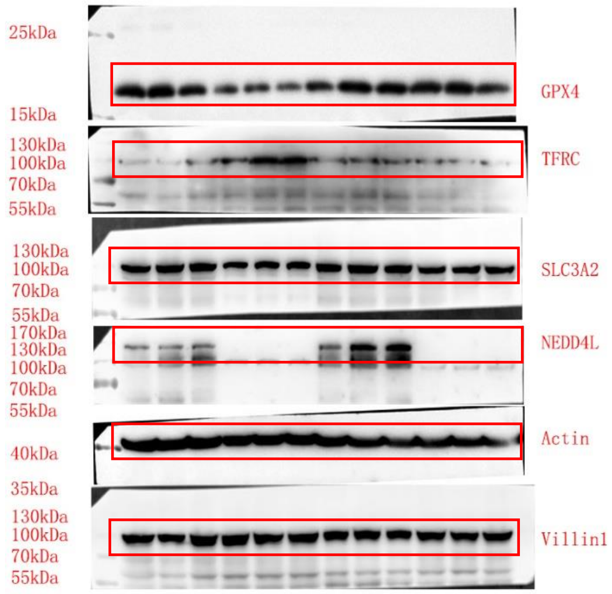

Supplement: Unedited blot and gel images [file jci-135-173994-s153.pdf]
